# AI-assisted forecasting in microsurgery: A dual-component framework for global publication trends

**DOI:** 10.1371/journal.pone.0357186

**Published:** 2026-08-28

**Authors:** Georgios Bouloukakis, Georgios Karamitros, Gregory A. Lamaris, Wesley P. Thayer, Galen Perdikis, Feng Zhang, William C. Lineaweaver

**Affiliations:** 1 Department of Surgery, SUNY Downstate Health Sciences University, Brooklyn, New York, United States of America; 2 Department of Plastic Surgery, Vanderbilt University Medical Center, Nashville, Tennessee, United States of America; 3 Division of Plastic and Reconstructive Surgery, R. Adams Cowley Shock Trauma Center, University of Maryland Medical Center, Baltimore, Maryland, United States of America; Shuguang Hospital Affiliated to Shanghai University of Traditional Chinese Medicine, CHINA

## Abstract

**Background:**

Artificial intelligence (AI)-assisted approaches may allow surgical research trends to be analyzed at scale and projected over time. However, their use in forecasting the evolution of microsurgical scholarship remains limited. This study developed an AI-assisted bibliometric framework to characterize and project global clinical and experimental microsurgery publication trends.

**Methods:**

PubMed metadata from 20 microsurgery-relevant surgical journals were extracted for 2010–2024 using an automated Python-based retrieval algorithm. A rule-based contextual key-word classifier using a predefined microsurgery keyword taxonomy was applied to identify relevant publications. Candidate forecasting models included linear regression, quadratic regression, autoregressive integrated moving average, and Holt’s exponential smoothing. Model performance was compared using *R*^2^, root mean square error, mean absolute error, and Akaike information criterion. Forecasts were generated through 2030 and reported with 95% confidence intervals.

**Results:**

The framework processed 90,902 records, of which 83,133 underwent contextual text classification after exclusion of incomplete metadata. A final analytic dataset of 11,561 microsurgery publications with verifiable first-author country attribution was identified. Classification validation using a stratified sample of 4,441 records demonstrated 95.2% agreement with the human-reviewed reference standard (95% CI, 94.5%–95.8%; Cohen’s *κ* = 0*.*826). Annual microsurgery publications increased from 611 in 2010–973 in 2024, representing a 59.3% increase. Temporal validation supported short-horizon stability of the linear projection model. In fixed temporal holdout testing, the linear model achieved RMSE 40.5, MAE 36.2, MAPE 4.0%, and 95% prediction-interval coverage of 100%. Through 2030, publication activity is projected to increase, with lymphatic microsurgery showing the greatest relative thematic growth (+36.0%), followed by technological and operative innovation (+21.7%).

**Conclusion:**

This study presents an AI-assisted bibliometric workflow for characterizing and projecting microsurgical publication trends. By integrating automated PubMed metadata extraction, contextual keyword-based classification, human-reviewed validation, and conventional statistical forecasting, the workflow enables reproducible assessment of publication activity across time, geography, authorship, and thematic domains. The resulting estimates should be interpreted as conditional projections under observed historical trends rather than deterministic predictions of future scientific activity, innovation, or leadership. This approach may assist surgeons, clinician-scientists, and interdisciplinary research teams in summarizing research patterns, identifying areas of increasing scholarly activity, and informing future collaborative planning.

## 1. Introduction

The next frontier in surgical science lies not only in measuring what has already been achieved, but also in understanding where scientific activity is likely to move next [1]. In parallel with its expanding role in diagnostics, operative planning, imaging, and clinical decision support, ar-tificial intelligence (AI) is increasingly being applied to the study of biomedical knowledge itself [1–3]. Within academic surgery, this creates an opportunity for bibliometric analysis to evolve from retrospective mapping toward more dynamic, forward-looking models of scholarly activity [4,5]. For surgeons, clinician-scientists, and interdisciplinary medical teams, such models may help identify which areas of investigation are expanding, which fields are stabilizing, and where future collaboration, training, and research investment may be most strategically directed.

Traditional bibliometric studies have provided valuable insight into publication volume, authorship patterns, citation activity, and geographic productivity. However, most remain inherently retrospective. They describe what has already occurred, but they rarely provide a reproducible framework for projecting how research activity may evolve over time. This limitation is particularly relevant in microsurgery, where scientific output is distributed across reconstructive surgery, hand surgery, peripheral nerve surgery, lymphatic reconstruction, transplantation, trauma, oncology, vascular surgery, and operative technology. Because microsurgical research is not confined to a single specialty or journal family, its global trajectory can be difficult to characterize using conventional manual review or narrow database searches alone.

Accurate analysis of this literature requires both scale and contextual precision. Large datasets must be extracted efficiently while preserving the semantic meaning that distinguishes microsurgery-relevant publications from adjacent surgical literature. This distinction is clinically important: articles involving lymphatic reconstruction, free-flap surgery, replantation, peripheral nerve reconstruction, perforator flaps, supermicrosurgery, surgical imaging, and operative innovation may all belong to the broader microsurgical ecosystem, even when they are indexed under different spe-cialties. Automated extraction can improve scale and reproducibility, but simple retrieval alone is insufficient unless paired with transparent contextual classification. Text-mining and semantic modeling approaches provide a practical method for organizing large biomedical corpora while retaining domain-specific meaning [6–9].

To address this gap, we developed an AI-assisted bibliometric forecasting framework that integrates automated PubMed metadata extraction with contextual text interpretation and statistical forecasting. The framework was designed for an interdisciplinary medical and surgical readership rather than as a purely technical machine-learning paper. Accordingly, AI is used here as a scalable analytic aid for biomedical literature processing, not as an autonomous clinical decision-making system. The extraction component allows rapid retrieval and structuring of large publication datasets [4], while the contextual classification component identifies microsurgery-relevant articles using a predefined thematic taxonomy. The resulting dataset is then analyzed using comparative forecasting models to generate projections of publication activity through 2030. These projections should be interpreted as trend-based estimates under observed historical assumptions, rather than deterministic predictions of future scientific impact.

Microsurgery was selected as the exemplar field because it represents a technologically complex and clinically interdisciplinary domain, encompassing reconstructive, lymphatic, peripheral nerve, traumatic, oncologic, and innovation-driven research [10]. The field is also highly responsive to institutional expertise, technological diffusion, training infrastructure, and cross-specialty collaboration. These characteristics make microsurgery a useful model for studying how surgical sub-specialties evolve over time. While prior bibliometric approaches have mapped past productivity, there remains a need for methods that can characterize temporal growth, geographic redistribution, and thematic expansion in a reproducible and clinically interpretable manner.

The objective of this study was to develop and apply an AI-assisted framework to characterize global microsurgery-related publication activity from 2010 to 2024 and generate projections through 2030. Specifically, we sought to determine how global microsurgical publication output has changed over time, which countries and income groups contribute most substantially to cur-rent and projected research activity, which thematic domains demonstrate the strongest projected growth, and whether an automated literature-processing workflow can provide clinically meaningful forecasts for surgeons and research teams. By applying this framework to more than 90,000 PubMed records across 20 microsurgery-relevant journals, this study aims to move beyond static bibliometric description toward a reproducible model for projecting the direction of microsurgical scholarship. More broadly, it proposes a practical paradigm for using AI-assisted literature analytics to support strategic research planning, collaboration, and academic development across reconstructive and allied surgical disciplines.

The main contribution of this study is the development and application of a reproducible, AI-assisted bibliometric workflow for microsurgery research assessment. First, we constructed a large PubMed-derived dataset of microsurgery-related publications across 20 journals using auto-mated metadata extraction and rule-based contextual keyword classification. Second, we validated the classification process against a human-reviewed reference standard and characterized classification performance using agreement, Cohen’s *κ*, sensitivity, specificity, predictive values, and false-positive and false-negative rates. Third, we integrated comparative statistical forecasting with temporal validation to generate exploratory projections of microsurgical publication activity through 2030. Fourth, we examined these trends across geographic, economic, authorship, and thematic dimensions, allowing the framework to identify areas of increasing scholarly activity and persistent disparities within the global microsurgery literature. Together, these contributions ex-tend prior retrospective microsurgery bibliometric studies by providing a transparent and clinically interpretable workflow for summarizing historical patterns and generating cautious, trend-based projections.

The remainder of the paper is organized as follows. The Literature Review and Related Work section summarizes prior work in surgical bibliometrics, automated metadata extraction, text mining, and forecasting-based research assessment. The Methods section describes the journal sampling frame, PubMed metadata extraction, rule-based contextual classification strategy, validation process, thematic-domain construction, authorship-based human-capital definition, and statistical forecasting approach. The Results section presents the dataset construction process, classification performance, temporal publication trends, geographic and income-group distributions, model-validation results, authorship patterns, and projected thematic growth. The Discussion section interprets these findings in relation to prior microsurgery and surgical scientometric literature, outlines the clinical and research-planning implications of the workflow, and discusses the assumptions and limitations of exploratory publication forecasting. The Conclusion summarizes the main findings and the potential role of AI-assisted bibliometric workflows in supporting future microsurgery research assessment.

## 2. Literature review and related work

Bibliometric analysis has become an important method for evaluating the evolution of medical and surgical research. In surgery, prior studies have used bibliometric and web-based metadata extraction approaches to quantify publication output, authorship patterns, international collaboration, research productivity, and disparities in academic representation [4]. In particular, large-scale surgical metadata extraction using web scraping was previously introduced by this group as a scalable method for collecting and structuring PubMed-indexed surgical publications, demonstrating that automated extraction can transform months of manual curation into reproducible datasets generated within hours [4]. This methodological foundation has since been applied to evaluate re-search productivity, human capital, collaboration, and academic output across surgical disciplines [4,11–14].

More recently, this line of work has been extended from retrospective surgical bibliometrics toward forecasting models in the aesthetic surgery literature [15]. These studies illustrate the potential value of using large-scale publication metadata not only to describe what has already occurred, but also to estimate how academic fields may evolve under observed historical trends. However, most existing bibliometric studies remain primarily descriptive, focusing on publication counts, citations, authorship, journals, institutions, or countries. Similarly, prior microsurgery-related bibliometric analyses have largely mapped historical productivity rather than projecting future geographic or thematic trajectories [16]. As a result, the literature provides useful insight into where microsurgical research has been, but less guidance regarding where the field may be moving.

AI-assisted text mining and natural language processing provide a practical approach for organizing large biomedical corpora while preserving domain-specific meaning [6–9]. In the present study, these methods are used as an analytic aid for surgeons and interdisciplinary research teams, not as an autonomous clinical or technical AI system. The goal is to improve the scale, reproducibility, and thematic resolution of literature-based research assessment while maintaining clinical interpretability.

The novelty of the present work lies in applying this framework specifically to microsurgery, a field that spans reconstructive, lymphatic, peripheral nerve, traumatic, oncologic, and technology-driven research. By integrating automated PubMed metadata extraction, contextual classification of microsurgery-relevant articles, and comparative forecasting models, this study extends prior ret-rospective bibliometric work into a clinically interpretable projection framework. This approach is intended to help surgeons, clinician-scientists, and interdisciplinary medical teams identify emerg-ing research domains, recognize persistent geographic disparities, and support strategic planning across reconstructive and allied surgical fields.

## 3. Methods

### 3.1 Study design and conceptual framework

This study developed an AI-assisted forecasting model to analyze and predict the growth of clinical and experimental microsurgical publications from 2010 to 2024, with projections through 2030. The analytic framework integrates automated data extraction, contextual classification, and pre-dictive modeling to generate a temporally dynamic and geographically resolved map of global publication activity [4,17]. The objective was not merely to describe existing research patterns but to anticipate future trajectories in scientific output and authorship, transforming bibliometrics into a predictive science.

### 3.2 AI-assisted dual-component framework

In this study, the term “dual-component framework” refers to an AI-assisted literature analytics workflow rather than a fully autonomous deep-learning system. The first component consisted of automated PubMed metadata extraction using a Python-based web-scraping algorithm. This approach has previously been introduced by our group in the surgical literature as a scalable method for extracting and structuring large volumes of PubMed-indexed surgical publication data (4). The algorithm retrieved article-level metadata, including title, abstract, authorship, journal, publication year, and affiliation fields, and converted these records into a structured analytic dataset. This component allowed high-throughput data collection across 20 microsurgery-relevant journals from 2010 to 2024.

The second component consisted of contextual text-mining classification and forecasting. Article classification was performed using a predefined microsurgery keyword taxonomy applied to titles and abstracts. The taxonomy included core microsurgical terms and stems such as micro-surg*, free flap*, perforator*, replantation*, and lymphatic*. Records meeting thematic criteria were classified as microsurgery-relevant, whereas articles without contextual relevance to reconstructive microsurgery, lymphatic surgery, peripheral nerve reconstruction, replantation, or related operative innovation were excluded.

No supervised neural network or large language model was trained de novo for article classification. Instead, the classification strategy was intentionally designed to be transparent, reproducible, and interpretable for a surgical and interdisciplinary medical readership. This distinction is important: the purpose of the framework was not to introduce a novel technical AI architecture, but to apply AI-assisted automation and text-mining methods to a clinically meaningful research question in microsurgical science.

Following classification, validated annual publication counts were analyzed using comparative forecasting models, including linear regression, quadratic regression, autoregressive integrated moving average, and Holt’s exponential smoothing [18]. Model performance was evaluated using *R*^2^, root mean square error, mean absolute error, and Akaike information criterion [19]. Linear regression was selected as the primary model because it provided the most favorable balance of fit, parsimony, stability, and interpretability for the 15-year annual dataset. Forecasts were generated through 2030 and reported with 95% confidence intervals.

Thus, the framework is best understood as AI-assisted rather than AI-autonomous. Its AI-enabled contribution lies in the scalable extraction, structuring, and contextual classification of a large biomedical literature corpus, followed by transparent statistical forecasting. This design was selected because the intended users are surgeons, clinician-scientists, and interdisciplinary medical research teams who require reproducible and clinically interpretable projections rather than a black-box computational model.

**Fig 1:**
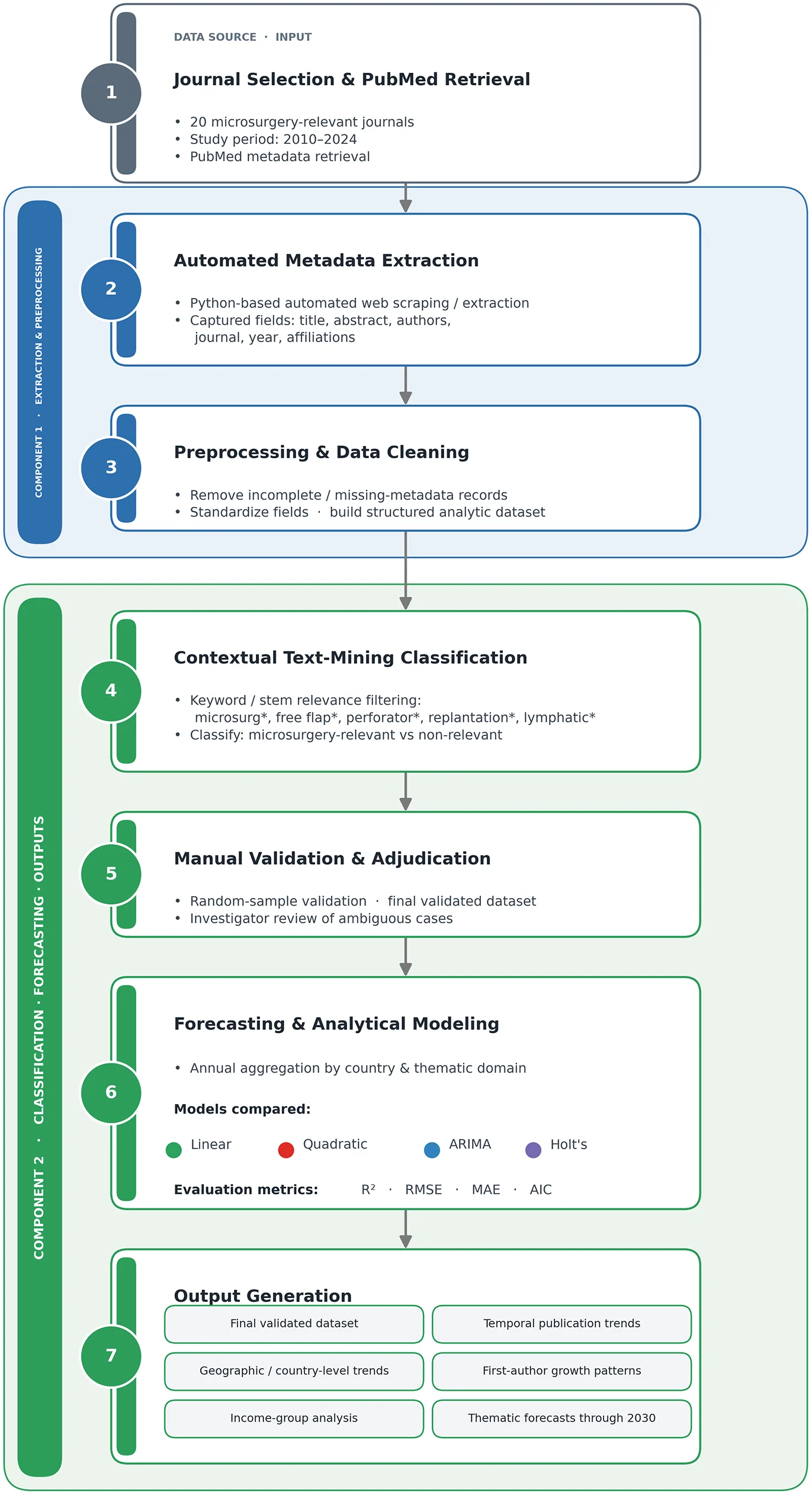
AI-enabled dual-component workflow for microsurgery publication forecasting. Notes: Component 1 includes journal selection, PubMed retrieval, automated metadata extraction, and preprocessing. Component 2 includes rule-based contextual keyword classification, human-reviewed validation, forecasting model comparison, and generation of temporal, geographic, authorship, income-group, and thematic projections through 2030.

The overall study architecture is presented in Fig 1. The framework comprised two sequential components. Component 1 included selection of the journal sampling frame, auto-mated PubMed metadata retrieval, extraction of article-level variables, and preprocessing of the resulting dataset. Component 2 included contextual text-mining classification, manual validation and adjudication, comparative forecasting, and generation of temporal, geographic, author-ship, income-group, and thematic projections. The framework was designed as an AI-enabled literature-processing workflow followed by transparent statistical forecasting, rather than as a fully autonomous machine-learning system.

### 3.3 Data source and journal selection

The PubMed database was selected for its comprehensive coverage and standardized metadata [20]. Twenty journals were included, representing the major specialties where microsurgical techniques are practiced—plastic and reconstructive surgery, hand surgery, orthopedic and trauma surgery, craniofacial reconstruction, otolaryngology, and vascular surgery. The complete journal list is provided in the Table S1 in S1 Appendix. This approach ensured inclusion of both core and peripheral research domains, capturing a broad cross-section of clinical and experimental microsurgical scholarship.

### 3.4 Data extraction and structuring

A custom Python-based algorithm was used to automatically retrieve publication metadata from PubMed for the years 2010–2024. Structured fields included title, abstract, authorship, institutional affiliation, journal, and publication year. The algorithm employed sustainable and ethical querying practices [21], processing more than 90,000 records in under four hours. This high-throughput extraction ensured scalability, reproducibility, and the statistical depth required for forecasting.

### 3.5 Contextual classification of microsurgery research

Article classification was performed using a rule-based contextual keyword classifier rather than a supervised machine-learning or deep-learning model. The classifier applied a predefined micro-surgery taxonomy to article titles and abstracts using case-insensitive matching with wildcard and stem expansion where appropriate. Articles with incidental keyword matches were classified according to primary study focus rather than keyword presence alone, and ambiguous records, records with limited abstracts, and records spanning multiple reconstructive domains were adjudicated using a title/abstract primary-focus rule [22].The keyword taxonomy contained 86 terms/stems across 9 thematic domains: general microsurgery terms, vascular/anastomotic keywords, neural microsurgery, replantation and limb salvage, lymphatic microsurgery, flap-based reconstruction, training/simulation/education, technological and instrumental innovation, and research methodology/outcomes. Terms were applied to article titles and abstracts using case-insensitive matching with wildcard/stem expansion where appropriate. Articles with only incidental keyword matches were classified according to primary study focus rather than keyword presence alone. Ambiguous records, records with limited abstracts, and records spanning multiple reconstructive domains were adjudicated using a title/abstract primary-focus rule.

Articles lacking thematic relevance were excluded. Classification performance was evaluated using a stratified validation sample drawn from all records that underwent relevance classification. Sampling was stratified by classifier label, publication year, and journal to represent both predicted relevant and predicted non-relevant records across the full study period. The final human-reviewed adjudicated label served as the reference standard for performance metrics. This ensured accurate differentiation of clinical and experimental microsurgical studies from unrelated surgical literature.

### 3.6 Construction of thematic domains

For thematic forecasting, individual keywords and related procedural terms were consolidated *a priori* into five clinically interpretable domains: (i) general free flap–based research, (ii) plantation and limb salvage, (iii) lymphatic microsurgery, (iv) technological and instrumental innovation, and (v) research methodology and outcomes. These domains were selected to represent the major recurring areas of microsurgical scholarship while maintaining sufficient annual publication counts for temporal modeling.

Conceptually related terms were grouped according to their dominant clinical or research application. Articles matching terms from more than one domain were reviewed on the basis of the principal study objective, operative procedure, and dominant emphasis of the title and abstract. Ambiguous records were adjudicated by the clinical investigators to assign the most appropriate primary thematic category and minimize double counting.

Categories were excluded from thematic forecasting when they were peripheral to the central scope of reconstructive microsurgery, had insufficient annual publication frequency for stable modeling, substantially overlapped with a broader retained domain, or lacked a consistent temporal signal across the study period.

### 3.7 Human capital in surgery: Operational definition

Human capital, broadly defined as the set of individual competencies contributing to knowledge production [23], was operationalized in this study using first authorship of scientific publications. This approach reflects established evidence that first authors typically represent the primary intellectual drivers of a project and are most likely to continue producing future research, thereby serving as a measurable proxy for a country’s capacity to generate new surgical knowledge [23–27].

The rationale for this proxy aligns with longstanding conventions in biomedical publishing, where first authorship is attributed to the contributor with the most substantial scientific input [28]. Empirical analyses further demonstrate that first authors—compared with middle authors—exhibit broader and more diverse contributions across study design, analysis, interpretation, and manuscript preparation, whereas intermediate authors more commonly participate in task-specific roles such as data collection [29].

This interpretation of first authorship as a proxy for national research-producing personnel is widely adopted in global surgical scholarship and has been used extensively to quantify research capacity across countries and subspecialties [25,30–32]. In this forecasting framework, first-author counts therefore serve as a standardized and reproducible indicator of human capital in microsurgery.

Importantly, this operational definition was intended to capture active research production rather than the full spectrum of academic leadership, mentorship, or collaboration. Senior authorship, corresponding authorship, total coauthorship, and coauthorship-network position reflect related but conceptually distinct dimensions of academic activity and are not directly interchange-able with first authorship. Senior authorship may more strongly represent supervision or laboratory leadership, while total authorship counts may combine substantive scientific leadership with more limited task-specific participation. Corresponding authorship is also inconsistently indexed across journals and databases, and shared first authorship is not uniformly represented in PubMed metadata.

For these reasons, alternative authorship measures were not treated as conventional sensitivity analyses of the same construct. Substituting senior, corresponding, or total authorship would evaluate different dimensions of academic contribution and could dilute the specific signal of research-producing personnel rather than test the robustness of the first-authorship proxy. Accordingly, first authorship was retained as the prespecified, literature-supported, and reproducible measure of national research-producing human capital, while its limitations are acknowledged explicitly.

### 3.8 Forecasting and predictive modeling

Validated publication data were aggregated annually by country and analyzed using time-series forecasting to model historical trends and project microsurgical research output through 2030 [33]. To determine the most appropriate forecasting strategy, four commonly used approaches were com-pared: (i) simple linear regression, (ii) quadratic regression, (iii) autoregressive integrated moving average (ARIMA), and (iv) Holt’s exponential smoothing. Model performance was evaluated using complementary measures of predictive accuracy and model fit, including Root Mean Square Error (RMSE), Mean Absolute Error (MAE), and Akaike Information Criterion (AIC). These metrics quantify how closely each model reproduces observed data while accounting for unnecessary complexity.

Because only 15 annual observations were available, forecasting results were interpreted as short-horizon projections under observed historical trends rather than deterministic predictions. Model validation therefore focused on temporal back-testing within the observed period and trans-parent reporting of uncertainty and model assumptions. Temporal out-of-sample validation was performed using two complementary approaches. First, rolling-origin expanding-window validation was used, in which models were fit on earlier years and then used to forecast one-, two-, and three-year horizons against held-out observations. Second, a fixed temporal holdout analysis trained models on 2010–2019 and evaluated predictions against observed counts from 2020–2024. Out-of-sample performance was summarized using RMSE, MAE, MAPE, and 95% prediction-interval coverage. Residual diagnostics were performed for the selected linear model. Autocorrelation was assessed using Durbin–Watson and Ljung–Box testing [34], residual normality using the Shapiro–Wilk test [35], and heteroscedasticity using the Breusch–Pagan test [36].

Linear regression was selected as the primary projection model based on temporal validation performance, parsimony, stability, and interpretability rather than in-sample fit alone (Table 1). Although information criteria did not decisively separate all candidate models, the linear model per-formed well in rolling-origin and temporal holdout validation, substantially outperformed ARIMA and quadratic regression in the 2020–2024 holdout, and provided a directly interpretable annual growth estimate. Holt’s exponential smoothing demonstrated similar short-horizon performance, but linear regression was retained because it offered comparable predictive stability with a simpler and more clinically interpretable model structure. Forecast uncertainty was reported using 95% confidence intervals derived from model-based error estimates under standard regression assumptions. Accordingly, projections through 2030 were interpreted as conditional estimates under continuation of observed 2010–2024 publication trends rather than deterministic predictions of future scientific activity.

**Table 1:** Forecasting model comparison and temporal validation.

	Model Fit			Rolling-Origin Validation^a^			Temporal Holdout^b^				
Method	_*R*_2	AICc	∆AICc	RMSE	MAE	MAPE (%)	RMSE	MAE	MAPE (%)	95% PI coverage	Decision
Linear regression	0.870	118.27	1.33	54.23	44.35	5.31	40.53	36.20	4.02	100%	Primary model
Quadratic polynomial	0.872	121.90	4.96	95.21	80.67	9.42	170.58	158.46	17.48	0%	Not selected
ARIMA	0.666	116.94	0.00	88.15	71.44	8.16	111.52	103.60	11.41	100%	Not selected
Holt linear exponential smoothing	0.870	126.75	9.81	73.35	59.46	7.05	40.53	36.20	4.02	100%	Not selected

Notes: Models were evaluated using annual global microsurgery publication counts from 2010–2024 (n = 15; 11,561 total publications). RMSE and MAE are expressed in annual publication counts; MAPE is expressed as a percentage. AICc values were calculated using a uniform Gaussian residual-sum-of-squares approach with explicit parameter counts.

^a^Rolling-origin validation values are pooled across expanding-window forecasts at one- to three-year horizons, with 18 forecasts per model and a minimum training window of eight years.

^b^Temporal holdout validation was performed by training models on 2010–2019 and testing on 2020–2024. Prediction-interval coverage is based on five holdout observations and should be interpreted cautiously. The 0% coverage for quadratic polynomial regression indicates that none of the five held-out observations fell within its 95% prediction intervals; this model extrapolated a declining trend after 2019 while observed publication counts remained higher.

ARIMA had the lowest AICc but higher rolling-origin and holdout errors than linear regression. Holt’s exponential smoothing matched linear regression in the fixed holdout but had higher pooled rolling-origin error. Linear regression was retained as the primary projection model based on temporal validation performance, parsimony, stability, and direct interpretability of the annual growth slope rather than AICc alone. Projections through 2030 should be interpreted as conditional extrapolations under continuation of observed historical trends.

For analytic clarity and clinical interpretability, individual microsurgery keyword categories were consolidated into five domains: (i) general free flap–based research, (ii) replantation and limb salvage, (iii) lymphatic microsurgery, (iv) technological and instrumental innovations, and (v) research methodology and outcomes. Domains peripheral to the core scope or without consistent signal across the study period were excluded by design.

All countries with verifiable first-author affiliation data were retained for descriptive global summaries and World Bank income-group analyses. This approach avoided excluding low-output regions from aggregate estimates of global microsurgical research activity. However, because annual country-level publication counts were sparse for several countries, individual national projections were interpreted only for countries with sufficient publication activity to support stable trend visualization. Countries with very low or intermittent publication counts contributed to aggregate regional and income-group analyses but were not emphasized as stand-alone national forecasting results.

To evaluate macro-level differences in research capacity and output, countries were stratified by World Bank income classification (high, upper-middle, lower-middle, and low income) [37]. National publication rates were additionally normalized by population to estimate per-capita productivity. For income-group convergence analyses, group-level productivity was defined as publications per first author and modeled annually using linear regression, with forecasts used to quantify projected gap trajectories between income strata through 2030. All analyses and visualizations were performed in Python 3.11 using pandas, scikit-learn, and statsmodels [38,39].

### 3.9 Validation and quality assurance

**Fig 2:**
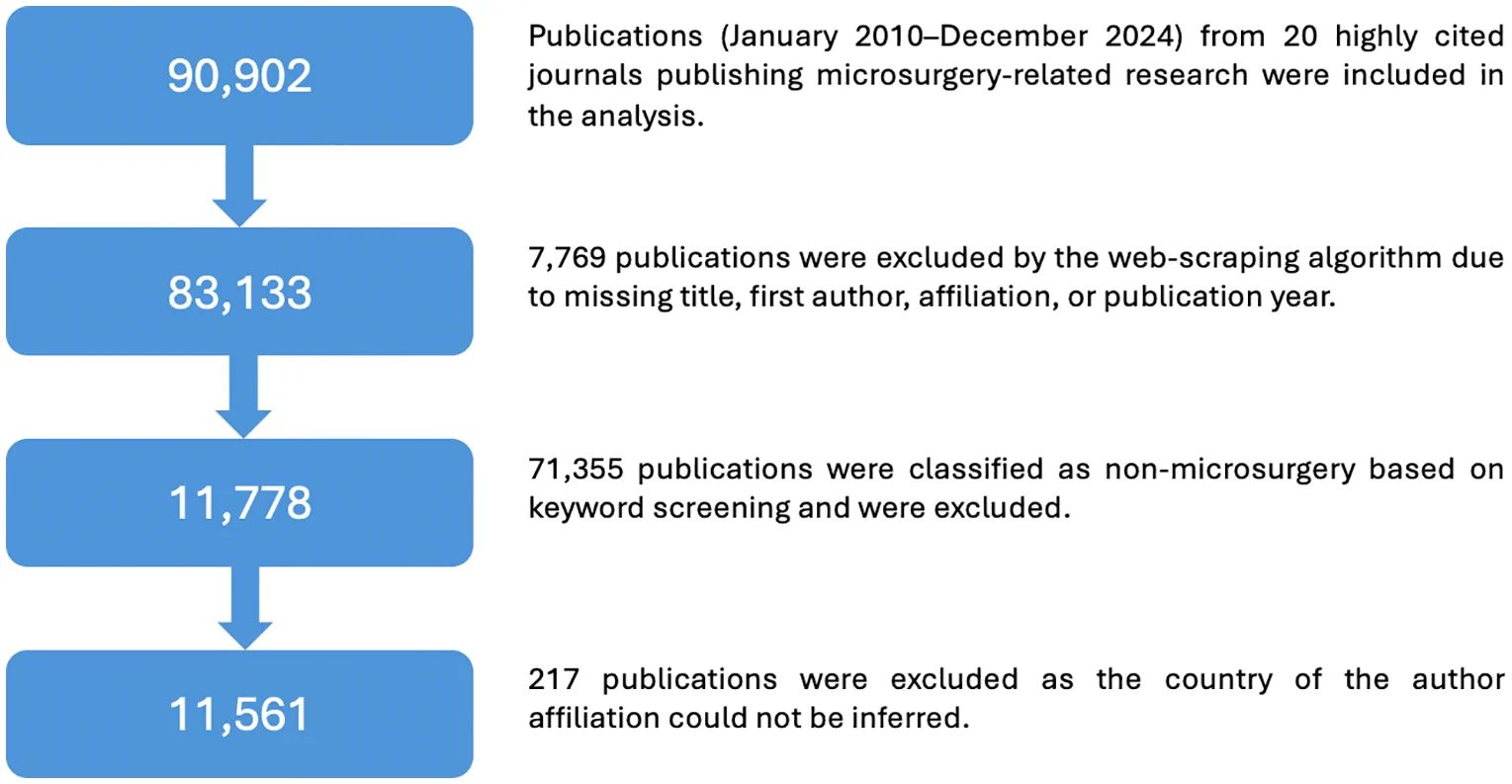
Flowchart of articles included in the study. Notes: A total of 90,902 records from 20 journals were retrieved for 2010–2024. After removal of 7,769 records with incomplete metadata, 83,133 records underwent contextual keyword classification. Of these, 71,355 were excluded as non-microsurgery records, leaving 11,778 microsurgery-related records. An additional 217 records without verifiable first-author country attribution were excluded, yielding a final analytic dataset of 11,561 publications.

Two complementary validation mechanisms ensured methodological fidelity [40]. Algorithmic cross-checks verified metadata completeness and prevented duplication during extraction. Ambiguous or borderline records were manually adjudicated by two investigators (G.K. and G.B.) to ensure thematic and geographic accuracy. To improve transparency and readability, the dataset construction process is summarized in Table 2. The complete article inclusion workflow is also illustrated in Fig 2. The final dataset formed the basis for the forecasting model, allowing accurate prediction of global trends in clinical and experimental microsurgical publications.

**Table 2:** Dataset construction summary.

Dataset element	Value
Study period	2010–2024
Number of included journals	20
Total PubMed records retrieved	90,902
Records removed due to missing metadata or affiliation	7,769
Records undergoing contextual text-mining classification	83,133
Records excluded as non-microsurgical	71,355
Microsurgery-related records after classification	11,778
Records excluded due to unverifiable first-author country	217
Final analytic dataset	11,561

### 3.10 Classification error analysis

To further evaluate the performance of the contextual classification framework, misclassified and borderline records identified during manual validation were reviewed qualitatively. The most common false-positive cases involved articles that contained microsurgery-related terms but were not primarily focused on reconstructive microsurgery. Examples included studies using terms such as “microvascular” in vascular, or basic science contexts, as well as articles mentioning flap terminology without reporting a microsurgical reconstructive procedure. These cases illustrate the challenge of distinguishing true microsurgical content from adjacent surgical or biomedical literature using terminology alone.

False-negative cases were less frequent but generally occurred when articles described microsurgical concepts without using the predefined keyword taxonomy in the title or abstract. Examples included studies of free-tissue transfer, lymphatic reconstruction, perforator-based reconstruction, or peripheral nerve repair that used indirect or nonstandard terminology. Additional challenging cases included articles spanning multiple reconstructive domains, abstracts with limited procedural detail, and studies in which the microsurgical component was present but not central to the article’s stated objective.

These errors were mitigated through iterative refinement of the keyword taxonomy and manual adjudication of ambiguous records by two investigators. The final validation sample demonstrated high agreement between the automated classification output and investigator consensus. Nevertheless, we recognize that a taxonomy-based contextual classifier may undercapture emerging terminology or overcapture adjacent literature when microsurgery-related terms are used non-specifically. For this reason, the classification framework should be interpreted as a transparent and reproducible screening method rather than a fully autonomous semantic model. Future versions of the framework may incorporate supervised machine-learning or embedding-based semantic classification to improve recall while preserving specificity.

## 4. Results

### 4.1 Dataset overview and AI system performance

The AI-assisted forecasting framework processed 90,902 records published between 2010 and 2024 across twenty microsurgery-related journals. After removal of 7,769 incomplete entries (missing metadata or affiliation), 83,133 articles underwent automated text-mining and contextual classification. The automated workflow excluded 71,355 non-microsurgical records, producing a validated dataset of 11,561 publications with verifiable first-author country attribution. The complete in-clusion workflow is illustrated in Fig 2.

Classification accuracy was evaluated using a stratified validation sample of 4,441 records, representing 5.3% of the 83,133 classified source records. The sample was stratified by classifier label, publication year, and journal. Against the human-reviewed reference standard, the classifier achieved 95.2% agreement (95% CI, 94.5%–95.8%) and Cohen’s *κ* of 0.826. The confusion matrix included 627 true positives, 112 false positives, 101 false negatives, and 3,601 true negatives. Sensitivity was 86.1%, specificity 97.0%, positive predictive value 84.8%, negative predictive value 97.3%, false-positive rate 3.0%, false-negative rate 13.9%, and F1 score 0.855.

The 213 classifier-reference discordant records included 112 false positives and 101 false negatives. False-positive classifications most commonly reflected adjacent non-microsurgical uses of terms such as “anastomosis” or “microvascular.” False-negative classifications generally involved records using indirect or nonstandard terminology for free-flap reconstruction, perforator-flap surgery, lymphatic microsurgery, or replantation. These findings were used to characterize the limitations of the taxonomy-based classifier and to guide interpretation of the final dataset.

### 4.2 Temporal dynamics in global microsurgery research

**Fig 3:**
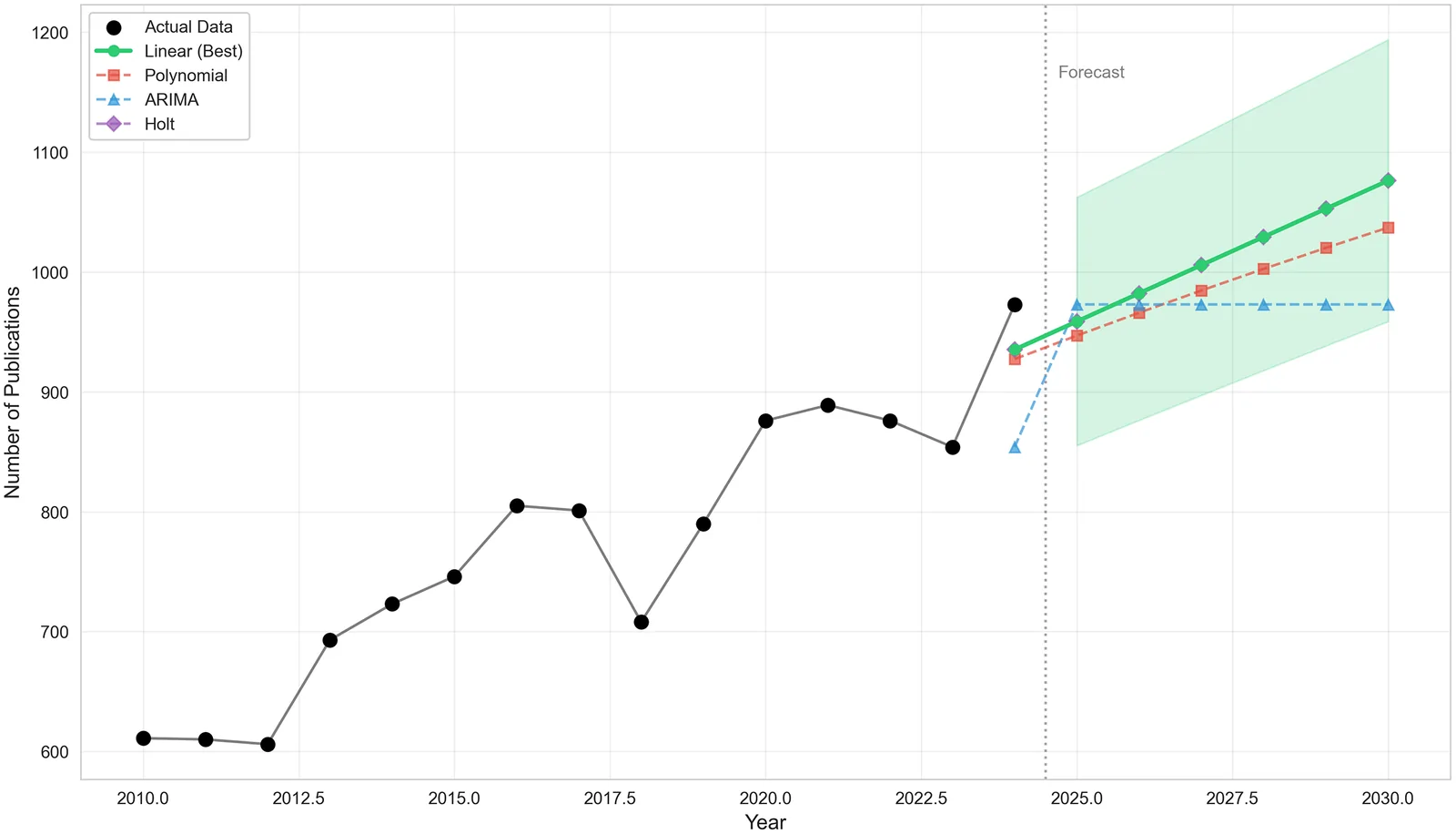
Yearly microsurgery-related publications trends. Notes: A total of 90,902 records from 20 journals were retrieved for 2010–2024. After removal of 7,769 records with incomplete metadata, 83,133 records underwent contextual keyword classification. Of these, 71,355 were excluded as non-microsurgery records, leaving 11,778 microsurgery-related records. An additional 217 records without verifiable first-author country attribution were excluded, yielding a final analytic dataset of 11,561 publications.

From 2010 to 2024, annual microsurgery publications rose from 611 to 973, a 59.3% increase. Growth accelerated sharply after 2019, coinciding with expanding interest in supermicrosurgery and lymphatic reconstruction. The temporal trajectory (Fig 3) reveals a steady upward slope with brief cyclical plateaus, reflecting sustained maturation rather than episodic surges. Forecast modeling anticipates continued expansion through 2030, with an estimated mean annual growth rate of 4.8% (95% CI, 4.2–5.3%).

### 4.3 Global distribution and research productivity

Eighty-six countries contributed to the global microsurgery literature. The United States accounted for one-third of publications (33.7%), followed by Japan (8.1%), China (7.7%), and the United Kingdom (6.2%). On a per-capita basis, Taiwan, Switzerland, and Australia exhibited the highest productivity, underscoring the efficiency of compact, well-resourced research systems. Across the dataset, 7,533 unique first authors were identified, yielding a mean productivity of 1.53 publications per author.

Forecast modeling projects that while North America and East Asia will retain leadership in absolute output, relative growth will accelerate in upper-middle-income economies—particularly China. By 2030, upper-middle-income economies are expected to contribute nearly one-quarter of global microsurgery publications. In contrast, sub-Saharan Africa and parts of South America are forecast to remain stagnant, revealing enduring disparities in research infrastructure and institutional capacity.

### 4.4 Economic stratification and research equity

**Fig 4:**
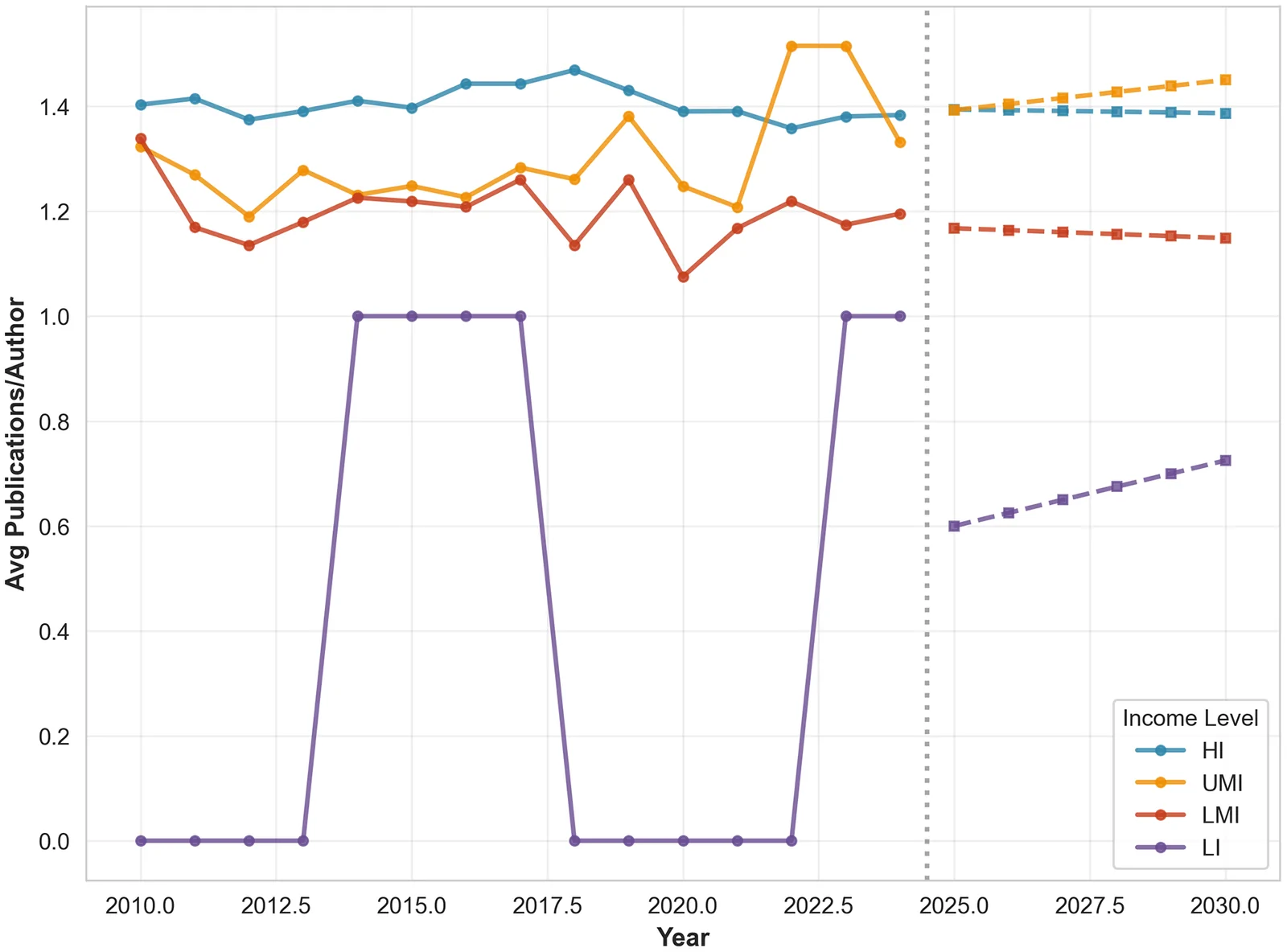
Productivity convergence analysis. Notes: Annual publication productivity is shown by World Bank income classification. Observed values are shown for 2010–2024, and model-based projections are shown for 2025–2030. The vertical dotted line marks the transition from observed data to projected estimates. Shaded bands represent 95% confidence intervals where applicable.

**Fig 5:**
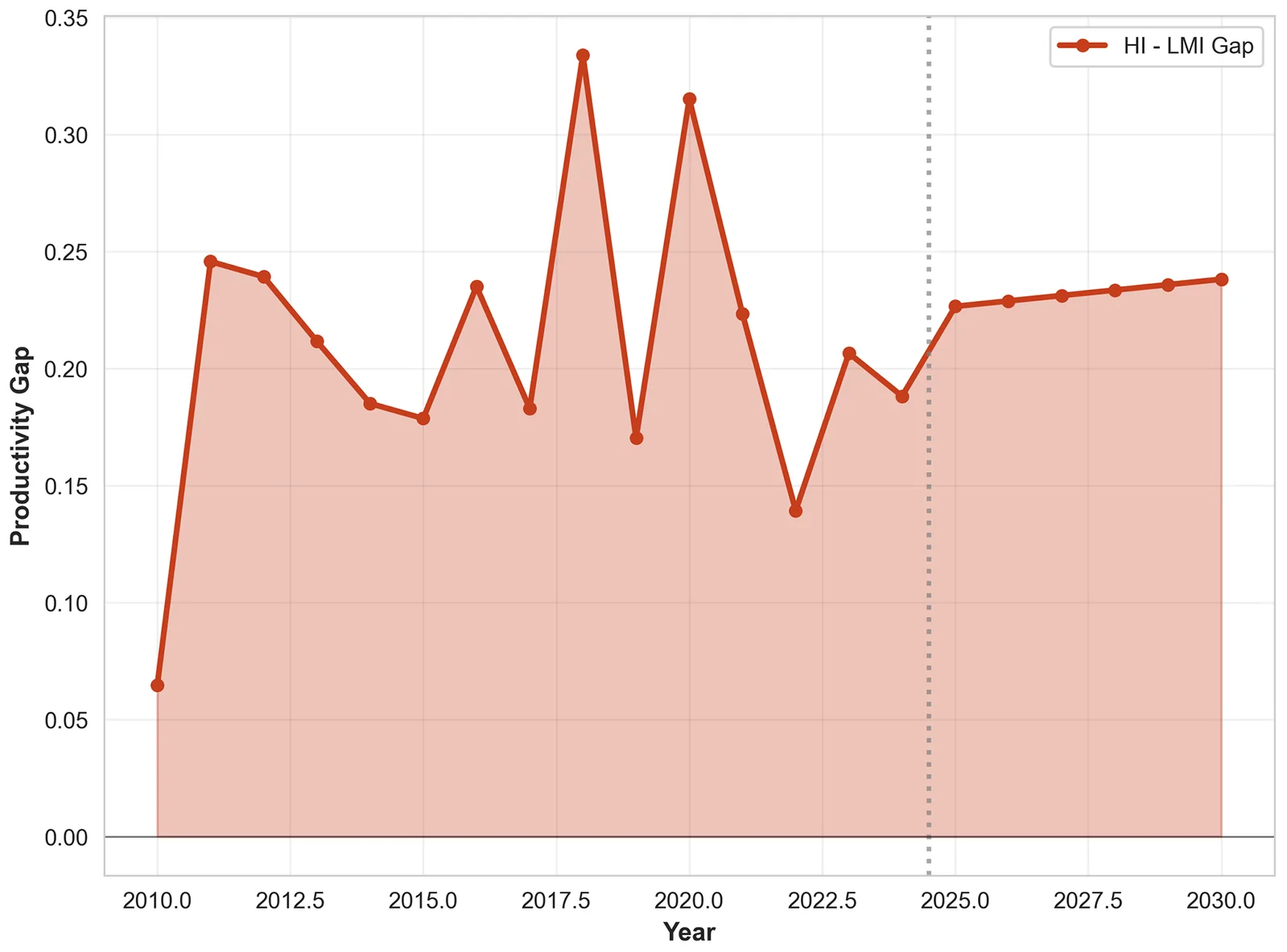
Productivity convergence analysis. Notes: The productivity gap is calculated as the difference in average annual publications per author between high-income and lower-middle-income countries. Observed values are shown for 2010–2024, and model-based projections are shown for 2025–2030. The vertical dotted line marks the transition from observed data to projected estimates.

When analyzed by World Bank income classification, high-income nations produced 8,033 publications (69.5%) and 5,210 first authors (69.2%), averaging 1.54 publications per author. Upper-middle-income countries accounted for 21.8% of global output, lower-middle-income countries 6.5%, and low-income countries 0.35%. Projections suggest a gradual but measurable rebalancing, with expanding contributions from upper-middle-income economies narrowing the global productivity gap. The distribution of research activity by income tier is shown in Figs 4 and 5.

### 4.5 Predictive model performance and future trajectories

**Fig 6:**
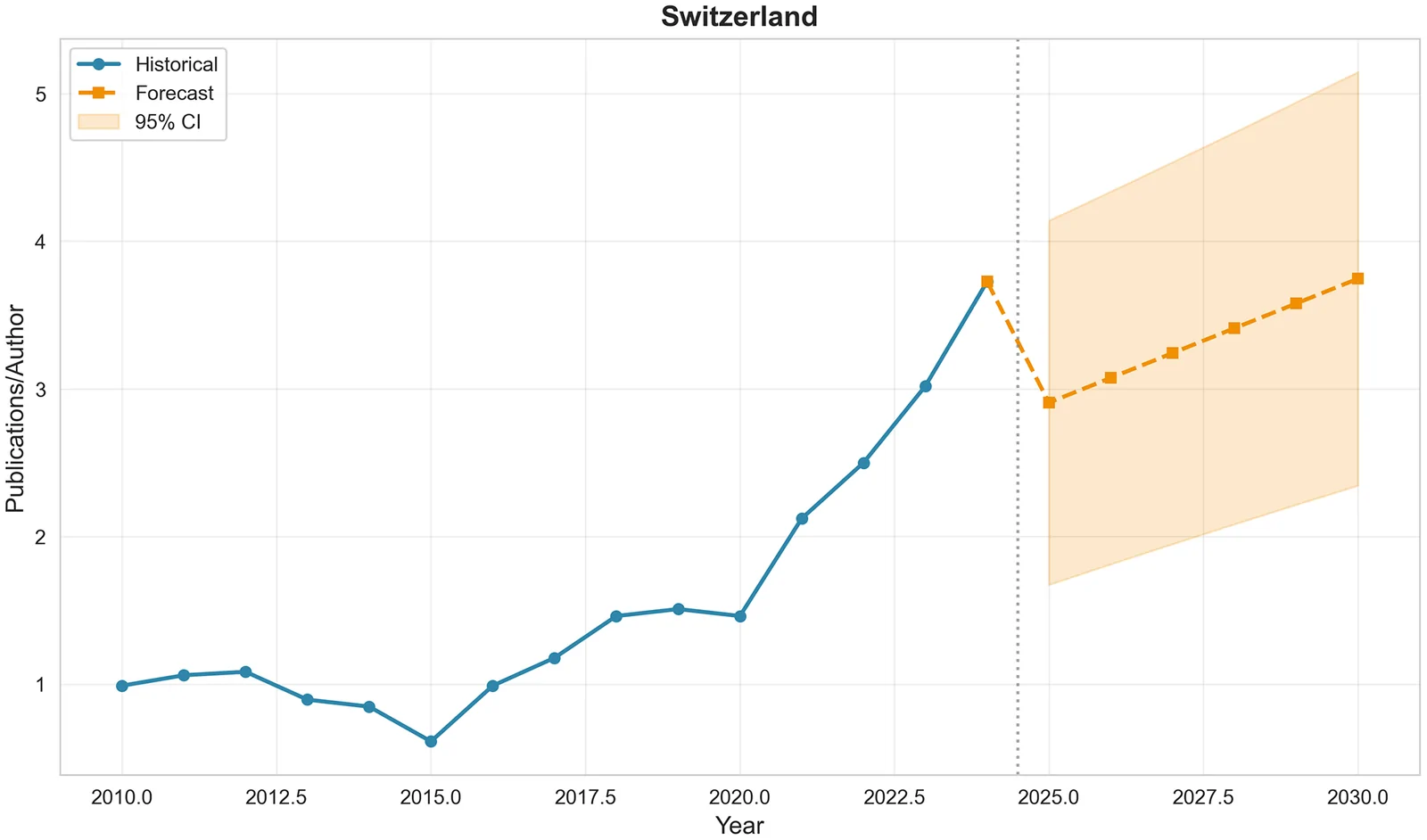
Productivity trend forecasting for Switzerland (2010–2030). Notes: Observed annual publications-per-author values for Switzerland are shown for 2010–2024, with model-based projections shown for 2025–2030. The vertical dashed line marks the transition from observed data to projected estimates. Shaded bands represent 95% confidence intervals.

**Fig 7:**
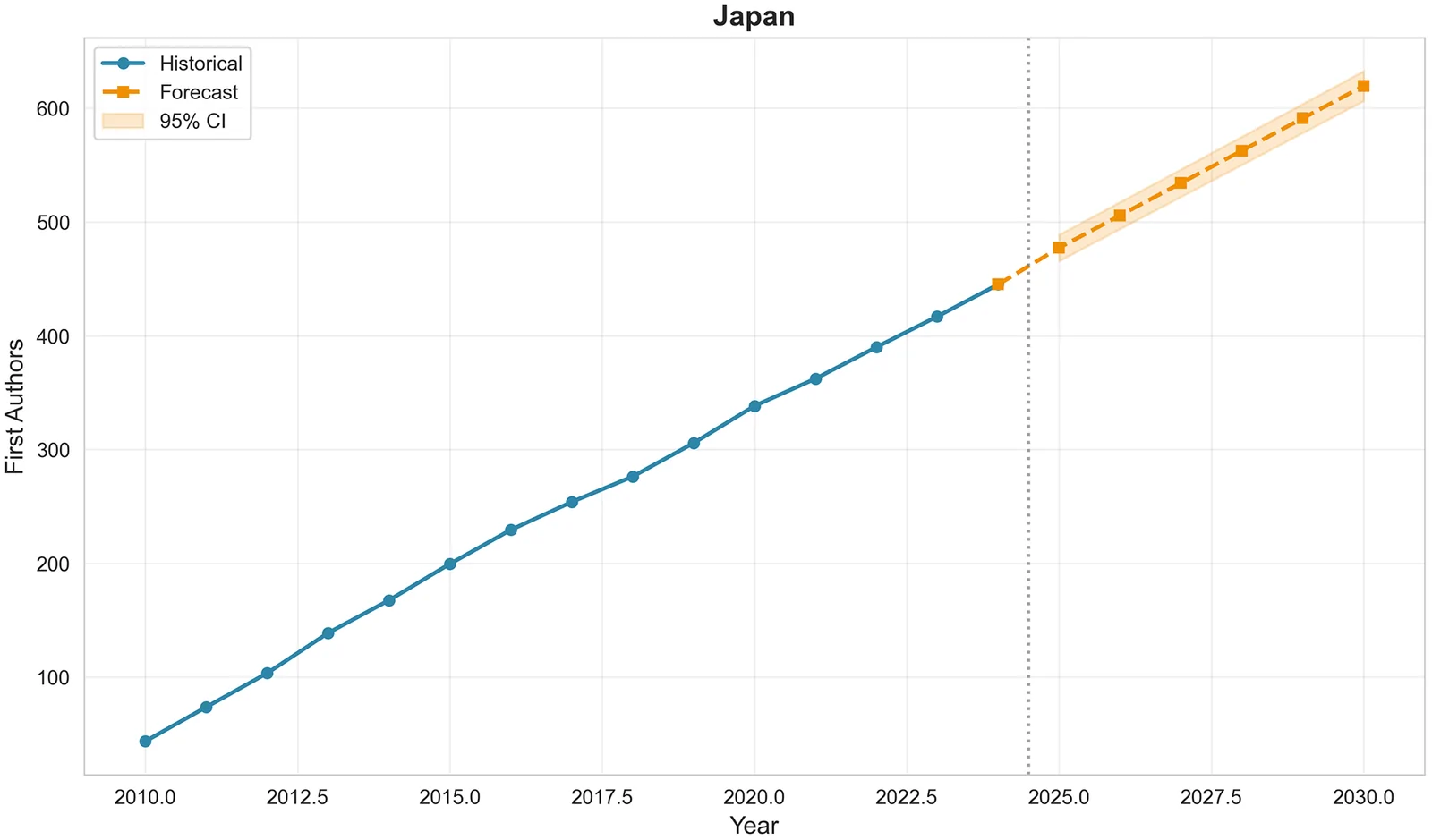
Productivity forecasting for Japan (2010–2030). Notes: Observed annual publications-per-author values for Japan are shown for 2010–2024, with model-based projections shown for 2025–2030. The vertical dashed line marks the transition from observed data to projected estimates. Shaded bands represent 95% confidence intervals.

**Fig 8:**
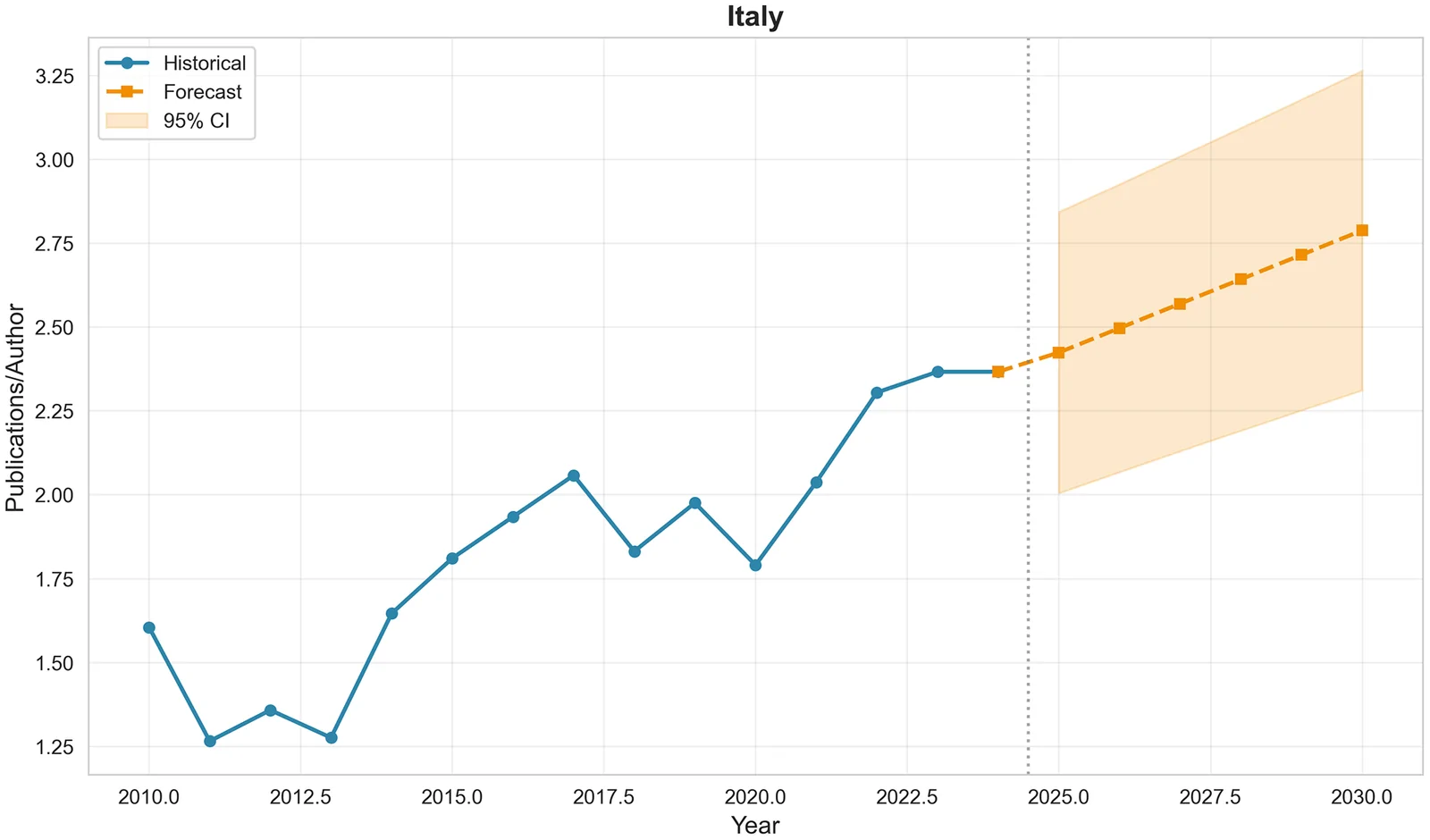
Productivity forecasting for Italy (2010–2030). Notes: Observed annual publications-per-author values for Italy are shown for 2010–2024, with model-based projections shown for 2025–2030. The vertical dashed line marks the transition from observed data to projected estimates. Shaded bands represent 95% confidence intervals.

**Fig 9:**
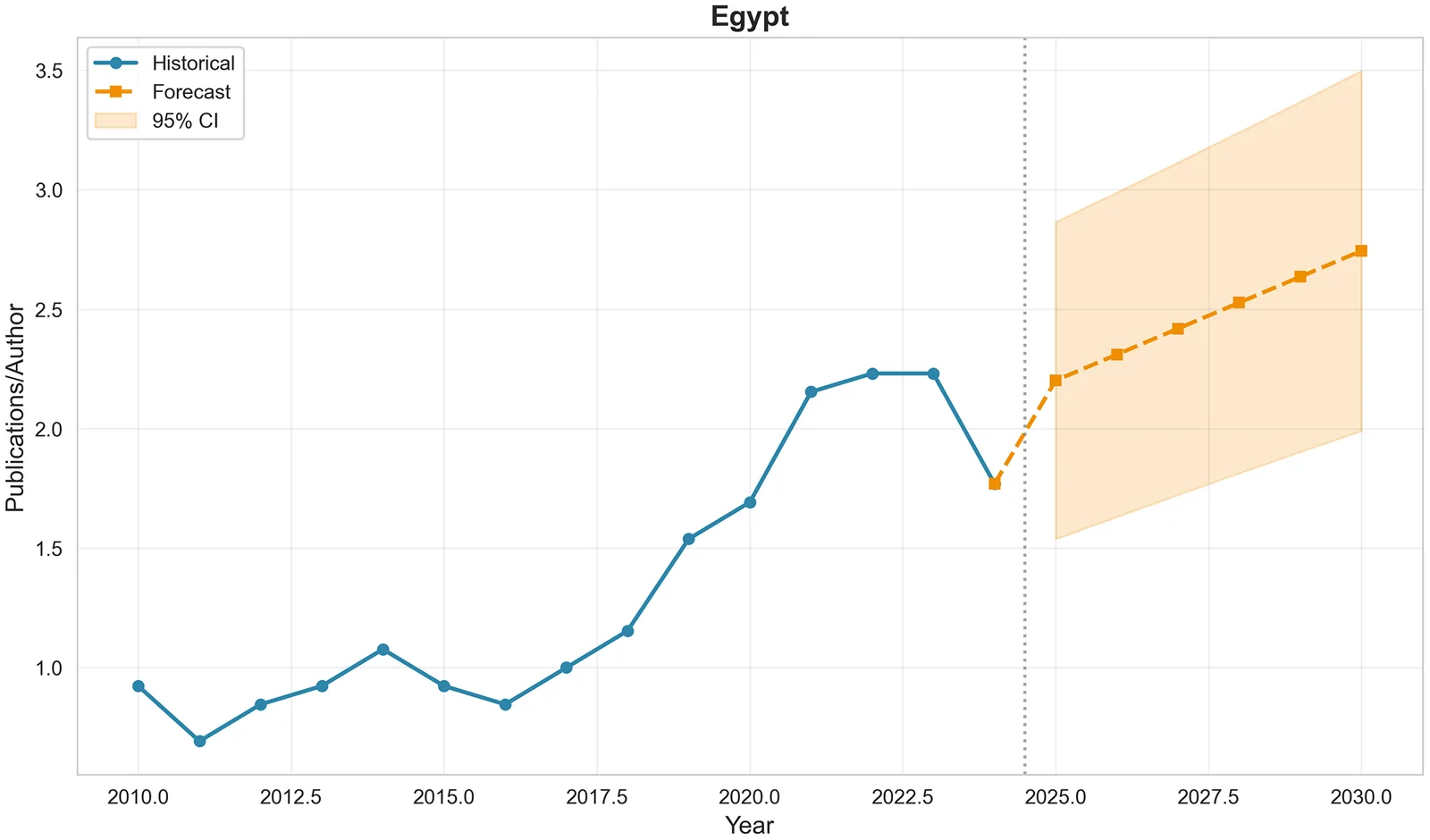
Productivity forecasting for Egypt (2010–2030). Notes:Observed annual publications-per-author values for Egypt are shown for 2010–2024, with model-based projections shown for 2025–2030. The vertical dashed line marks the transition from observed data to projected estimates. Shaded bands represent 95% confidence intervals.

**Fig 10:**
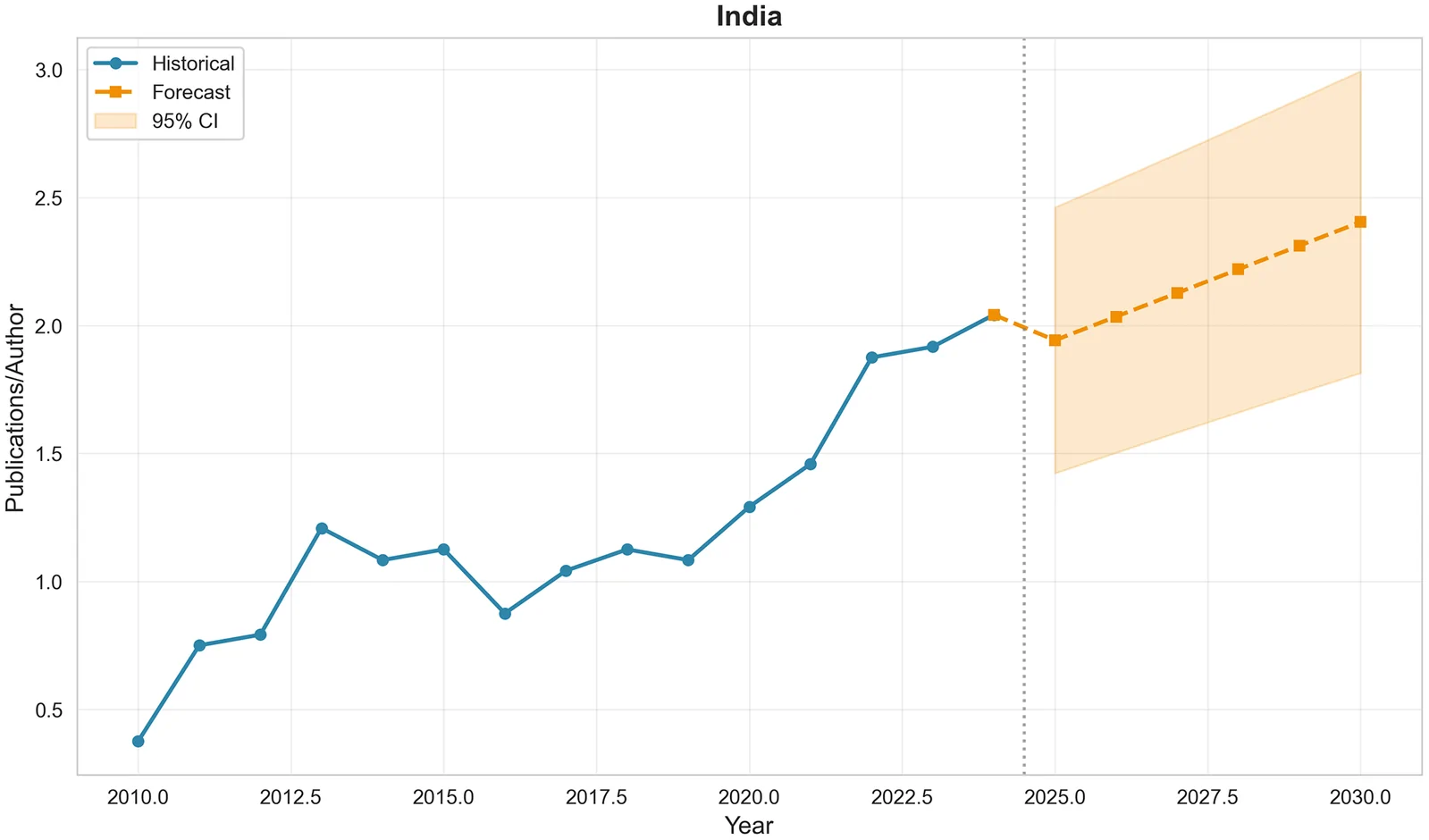
Productivity forecasting for India (2010–2030). Notes: Observed annual publications-per-author values for India are shown for 2010–2024, with model-based projections shown for 2025–2030. The vertical dashed line marks the transition from observed data to projected estimates. Shaded bands represent 95% confidence intervals.

**Fig 11:**
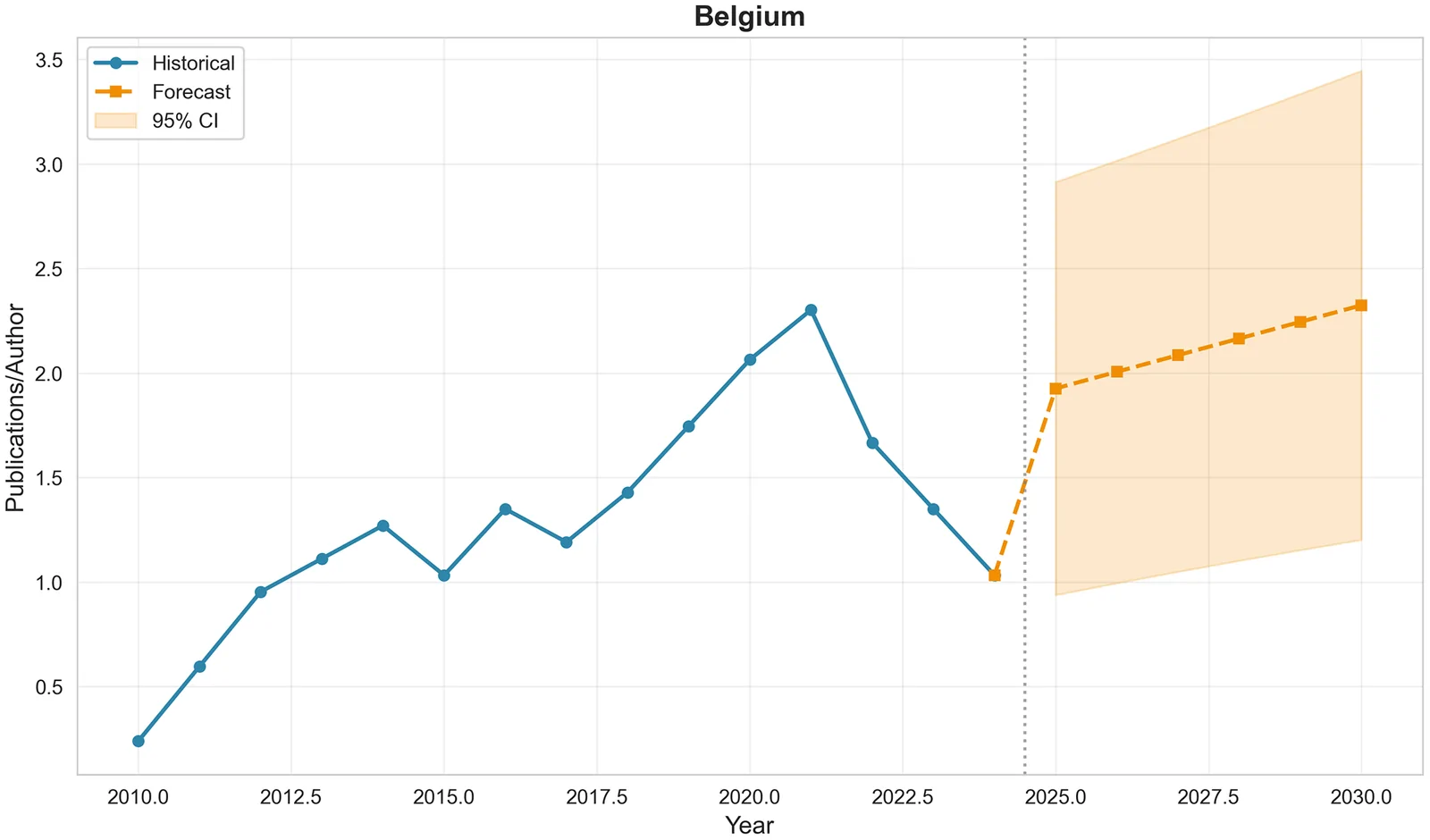
Productivity forecasting for Belgium (2010–2030). Notes: Observed annual publications-per-author values for Belgium are shown for 2010–2024, with model-based projections shown for 2025–2030. The vertical dashed line marks the transition from observed data to projected estimates. Shaded bands represent 95% confidence intervals.

The fitted models showed strong in-sample agreement with observed annual publication counts across income strata (mean *R*^2^ = 0.87). Forecast performance was evaluated using error metrics (R^2^, RMSE, MAE and AIC) during model selection (Table 1), and uncertainty was propagated as 95% confidence intervals in all projections. Projections indicate the cumulative number of included microsurgery publications over 2010–2030 will exceed 14,000, representing a sustained upward trajectory in research activity (Figs 6–11). This growth is expected to be driven primarily by East Asia and Western Europe, which together will account for more than half of global scholarly production by the end of the decade.

Temporal validation supported short-horizon stability of the linear projection model. In rolling-origin validation, one-year-ahead linear forecasts achieved RMSE 65.1, MAE 52.8, and MAPE 6.6% across seven validation origins; pooled across one- to three-year horizons, RMSE was 54.2, MAE 44.4, and MAPE 5.3%. In the fixed temporal holdout analysis, with models trained on 2010–2019 and evaluated on 2020–2024, the linear model achieved RMSE 40.5, MAE 36.2, MAPE 4.0%, and 95% prediction-interval coverage of 100%.

Linear regression was selected as the primary projection model based on temporal validation performance, parsimony, stability, and interpretability rather than in-sample fit alone. Although information criteria did not decisively separate all models, the linear model performed well in rolling-origin and temporal holdout validation, substantially outperformed ARIMA and quadratic regression in the 2020–2024 holdout, and provided a directly interpretable annual growth estimate. Residual diagnostics for the linear model were acceptable for a short annual series: *R*^2^ = 0*.*870, Durbin–Watson 1.78, Ljung–Box *p* = 0*.*218, Shapiro–Wilk *p* = 0*.*316, and Breusch–Pagan *p* = 0*.*425. No diagnostic test indicated statistically significant residual autocorrelation, non-normality, or heteroscedasticity.

**Fig 12:**
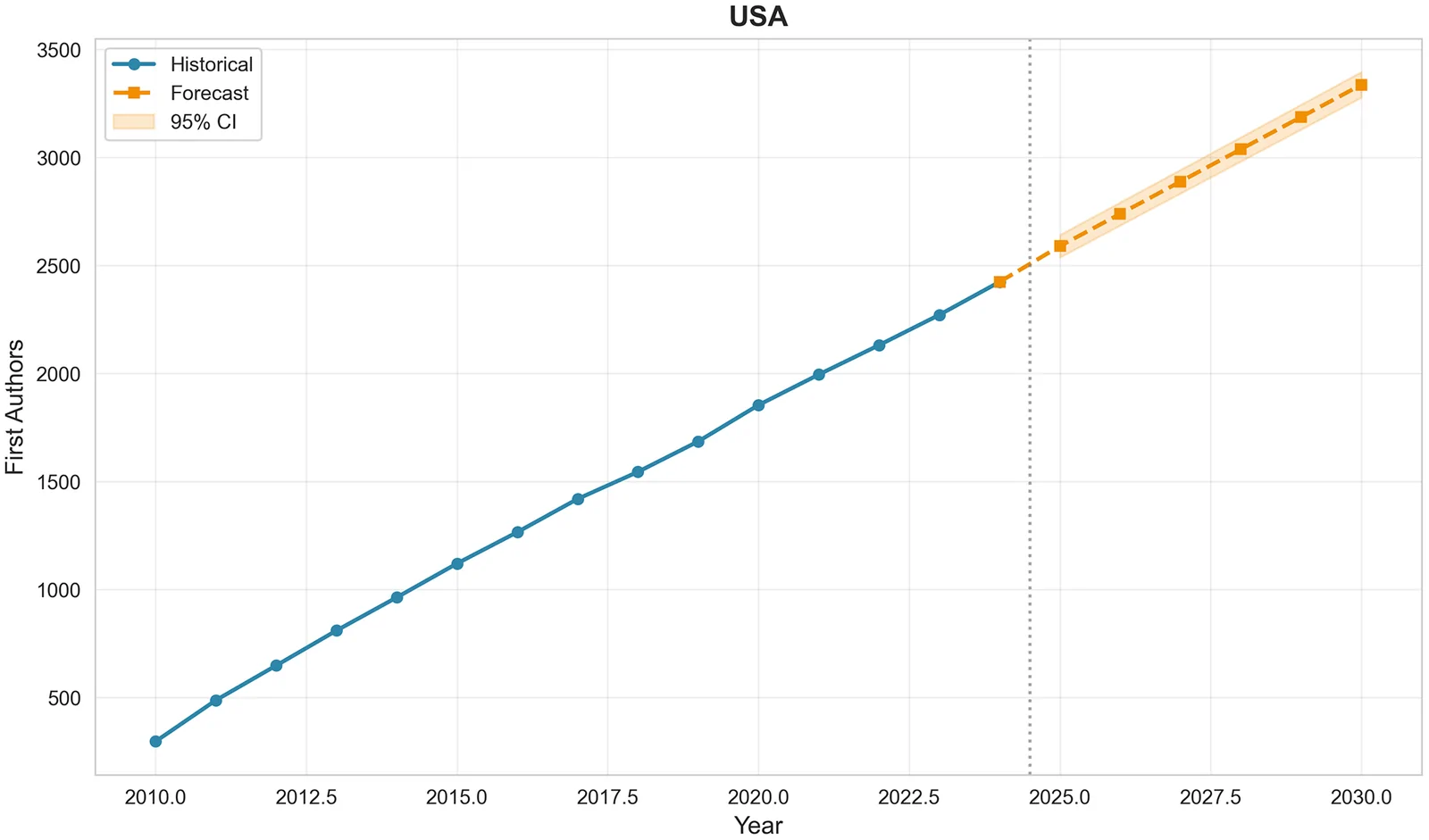
Author growth forecasting for the United States of America (2010–2030). Notes: Observed cumulative first-author counts for the United States are shown for 2010–2024, with model-based projections shown for 2025–2030. The vertical dashed line marks the transition from observed data to projected estimates. Shaded bands represent 95% confidence intervals.

**Fig 13:**
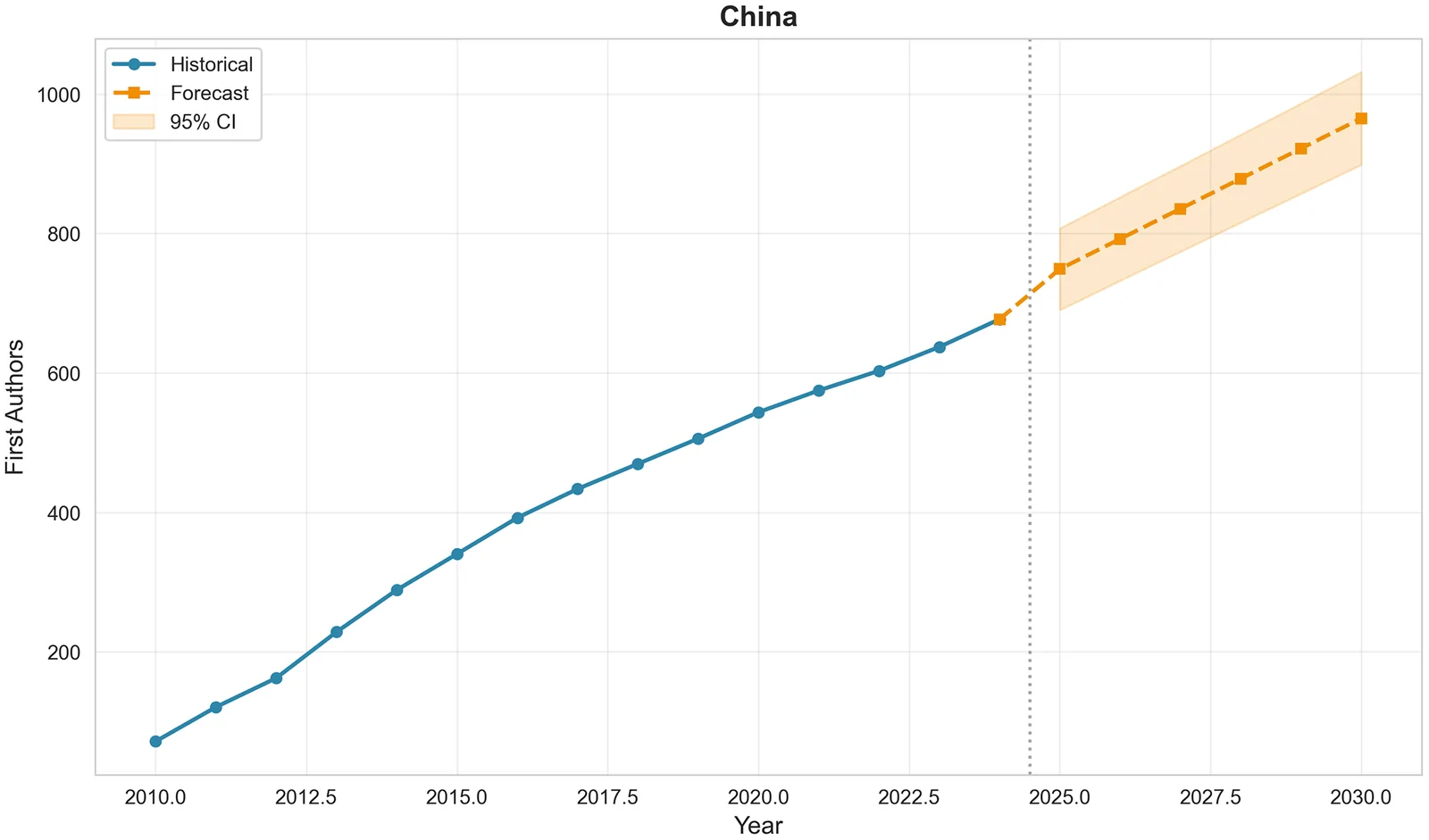
Author growth forecasting for China (2010–2030). Notes: Observed cumulative first-author counts for China are shown for 2010–2024, with model-based projections shown for 2025–2030. The vertical dashed line marks the transition from observed data to projected estimates. Shaded bands represent 95% confidence intervals.

**Fig 14:**
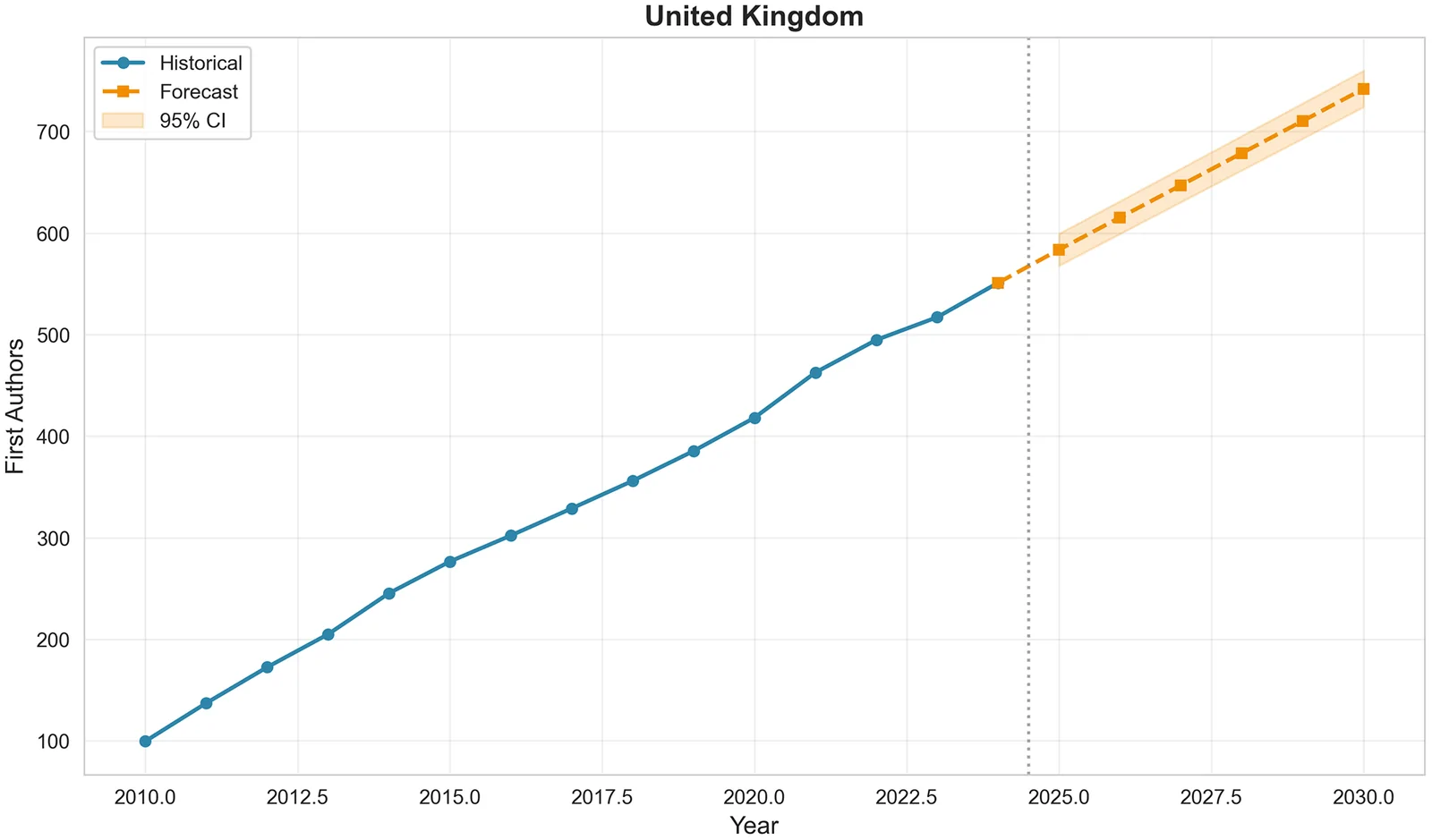
Author growth forecasting for the United Kingdom (2010–2030). Notes: Observed cumulative first-author counts for the United Kingdom are shown for 2010–2024, with model-based projections shown for 2025–2030. The vertical dashed line marks the transition from observed data to projected estimates. Shaded bands represent 95% confidence intervals.

Author growth patterns mirrored these trends. The United States, China, Japan, and the United Kingdom continue to dominate in absolute numbers, while Taiwan and Turkey show accelerating first-author expansion (Figs 12–14 and S3–S5 in S1 Appendix). The projected increase in author participation across upper-middle-income countries suggests a gradual diffusion of research capacity, even as high-income regions maintain their leadership in publication volume.

Forecasting of the Research Intensity Index—defined as publications per first author—revealed distinct national trajectories (Figure S1 in S1 Appendix). Switzerland displayed the steepest rise, with its index projected to exceed 8 by 2030, reflecting exceptional research efficiency within a concentrated author base. Japan, Sinagpore and the United States are expected to sustain moderate, steady increases, while Taiwan is projected to plateau, suggesting a saturation point in its research infrastructure.

Finally, country-specific growth forecasts (Figure S2 in S1 Appendix) illustrate divergent national trajectories. The United States and Japan are predicted to maintain compound growth in both publication output and author expansion. China’s growth curve remains steep, signaling continued large-scale investment in academic capacity, whereas South Korea and Taiwan show a shift toward consolidation rather than expansion. Western European nations, including Italy and Belgium, display accelerating productivity with moderate author growth—suggesting optimization rather than expansion of research systems (Tables S2–S4 in S1 Appendix).

### 4.6 Forecasted thematic growth in microsurgery research

**Fig 15:**
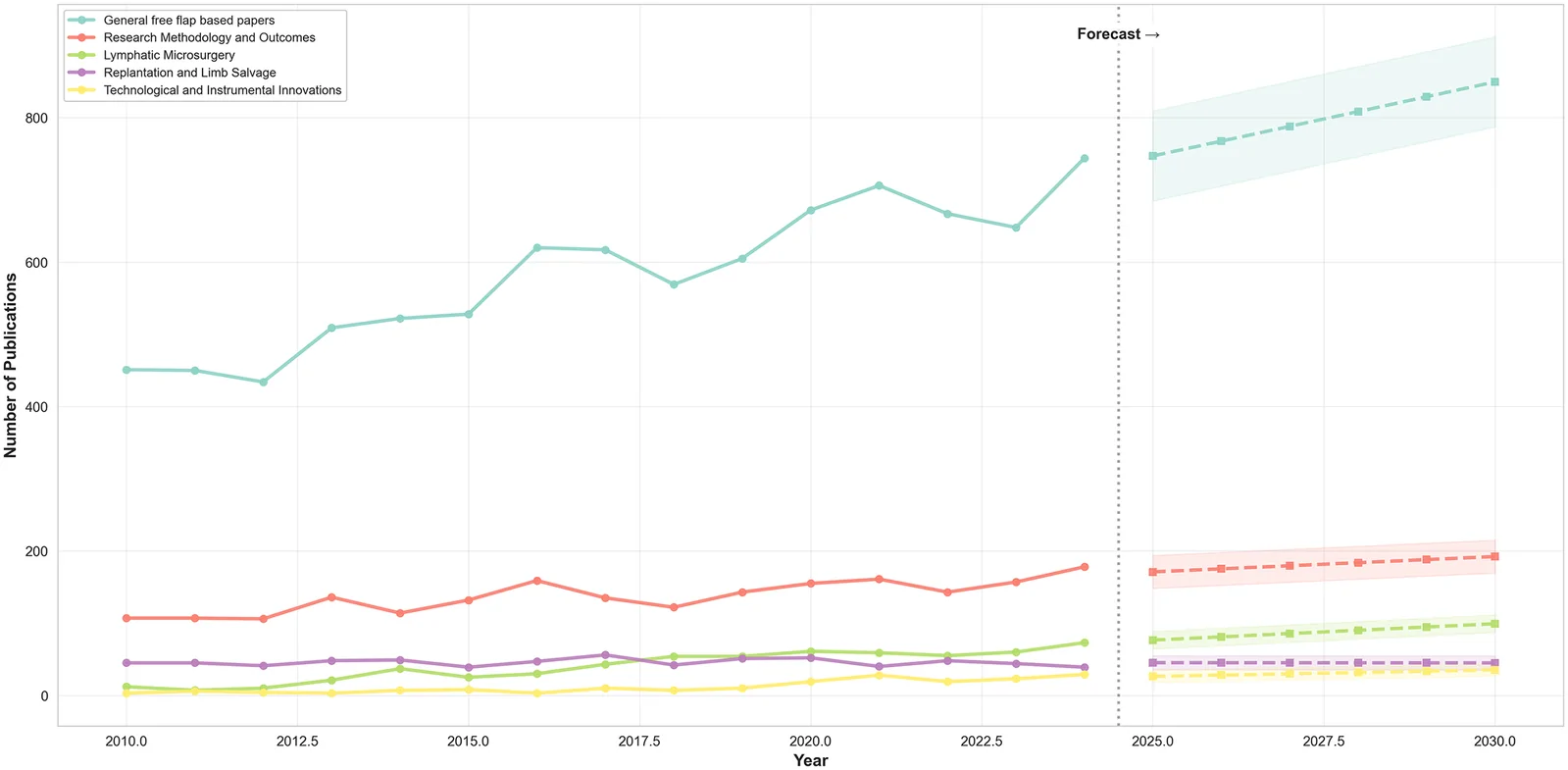
Forecasted growth by microsurgical subtheme (2010–2030). Notes: Solid lines show observed annual publication counts from 2010–2024, and dashed lines show model-based projections for 2025–2030. The vertical dotted line marks the start of the projection period. Shaded bands represent 95% confidence intervals.

The AI-assisted forecasting framework identified heterogeneous growth trajectories across micro-surgical research themes through 2030 (Fig 15 and Table 3). General free flap–based publications are projected to increase from 744 in 2024–850 in 2030 (+14.2%). Research methodology and surgical outcomes studies will expand modestly (+8.0%), while lymphatic microsurgery research demonstrates the highest relative growth (+36.0%). Technological and operative innovations are forecasted to rise by +21.7%, and replantation and limb salvage research remains stable over the projection period (Table 3). Overall, quantitative modeling indicates continued expansion and diversification of global microsurgical research output.

**Table 3:** Projected expansion of major microsurgical research domains through 2030.

Category	2024 Count	2030 Forecast	Growth Rate	% Change
General Free Flap-based Papers	743	850	+20.5/year	+14.2%
Research Methodology and Surgical Outcomes	178	192	+4.2/year	+8.0%
Lymphatic Microsurgery	73	99	+4.6/year	**+36.0%**
Replantation and Limb Salvage	39	45	−0.1/year	+15.2%
Technological and Operative Innovations	29	35	+1.8/year	**+21.7%**

Notes: Forecasted publication counts, annual growth rates, and percentage change are presented for each thematic category of microsurgical research.

## 5. Discussion

This study demonstrates how an AI-assisted bibliometric workflow can support large-scale assessment of microsurgical publication activity. By integrating automated metadata extraction, contextual keyword-based classification, human-reviewed validation, and conventional statistical forecasting, the framework provides a reproducible approach for examining historical and projected publication trends across time, geography, authorship, and research domains. This clinically oriented workflow offers surgeons, clinician-scientists, and interdisciplinary research teams a practical method for summarizing evolving research patterns and identifying areas of increasing scholarly activity within microsurgery [41]. The classification component should be interpreted as a transparent, rule-based contextual keyword approach rather than a trained semantic AI model. Its value lies in providing a reproducible screening and structuring method for a large PubMed-derived literature corpus, with human-reviewed validation used to assess classification performance and characterize misclassification patterns.

The 2025–2030 estimates should be interpreted in the context of the 15-year annual time series from which they were derived. Temporal validation supported short-horizon stability of the selected model within the observed dataset; however, projections beyond 2024 necessarily depend on the assumption that historical publication patterns continue over the forecast period. For this reason, the projected trajectories are best viewed as exploratory, trend-based estimates of scholarly activity rather than definitive forecasts of future scientific output, innovation, research leadership, or clinical impact.

Three major findings emerge. First, global microsurgical research continues to expand but remains geographically asymmetrical. Nearly 70% of publications originate from high-income countries, reflecting well-established academic infrastructures and funding ecosystems. However, upper-middle-income nations—most notably China and India—show the highest proportional ac-celeration, indicating a gradual redistribution of scientific momentum. The model predicts that by 2030, emerging economies will contribute substantially to global microsurgical output, marking a shift toward a more diversified research landscape.

Second, the predictive modeling component establishes a methodological precedent for using AI to forecast long-term trajectories in surgical investigation. By analyzing fifteen annual observations (2010–2024) of publication data, the model identifies inflection points in scholarly productivity—moments where research acceleration or stabilization occurs—providing early indicators of thematic maturity or emerging opportunity. This capability allows data-driven foresight for shaping funding allocation, training priorities, and collaborative frameworks within reconstructive science.

Third, the model’s thematic forecasts reveal distinct trajectories within the field. Lymphatic reconstruction exhibits the steepest projected ascent, reflecting the growing clinical adoption of physiologic and supermicrosurgical techniques [42]. Operative innovation and surgical technology show parallel expansion, driven by imaging, robotics, and precision instrumentation [43]. In contrast, established domains such as replantation and peripheral nerve microsurgery demonstrate plateauing productivity, suggesting domain consolidation rather than decline [44]. Collectively, these trajectories illustrate how AI-based forecasting can delineate emerging and mature fields within the evolving continuum of microsurgical research.

Beyond these empirical findings, this work represents a a reproducible approach for large-scale surgical publication analysis. The dual-AI design couples scale with semantic accuracy—automating large-scale data retrieval while ensuring contextual interpretation [4]; [45]. This architecture enables continuous, high-resolution monitoring of scientific ecosystems without sacrificing interpretive depth. The approach is inherently transferable, allowing similar forecasting applications across other surgical domains, including aesthetic, craniofacial, and regenerative medicine, where identi-fying emergent trends is essential for guiding innovation and equitable development.

The added value of this study should be interpreted in relation to prior surgical scientometric work. Previous microsurgery-related bibliometric analyses have provided important retrospective descriptions of publication productivity, influential articles, authorship patterns, countries, journals, and research themes [16]. However, these studies have primarily mapped where the field has been rather than modeling where it may be moving. In contrast, the present framework integrates automated metadata extraction, contextual microsurgery classification, and comparative forecasting to generate clinically interpretable projections of publication activity across geography and research domains. This distinction is important for surgeons and interdisciplinary research teams because trend-based forecasting may help identify emerging areas of scholarly activity, persistent geographic disparities, and future opportunities for collaboration and academic planning.

The workflow may be adaptable to other surgical and medical domains, but such transferability requires field-specific modification rather than direct reuse of the microsurgery classifier. The journal sampling frame would need to be redefined to capture the relevant specialty literature, and the keyword taxonomy would need to be reconstructed around the procedures, diagnoses, technologies, and terminology of the target field. Thematic domains would also require specialty-specific input to ensure that categories are clinically meaningful and sufficiently frequent for temporal modeling. Finally, the classification process would require renewed human-reviewed validation, including assessment of agreement, sensitivity, specificity, false-positive and false-negative patterns, and performance across journals and years.

This domain-specific adaptation is consistent with our prior applications of related publication-trend workflows in aesthetic surgery and craniofacial surgery [15,46]. In each setting, the analytic structure remained similar, but the journal selection, terminology, thematic grouping, and validation process were tailored to the clinical field under study. Accordingly, the present framework should be interpreted as a transferable workflow structure for AI-assisted bibliometric assessment, not as a universal classifier or forecasting model that can be applied unchanged across specialties. Future work may extend this workflow in several directions. First, transformer-based natural language processing models or large language models could be incorporated to improve semantic classification, particularly for articles using indirect, emerging, or nonstandard terminology. Such models would require careful training, calibration, and human-reviewed validation to ensure that improved recall does not come at the expense of interpretability or specificity. Second, future studies could expand the data source beyond PubMed by incorporating Scopus, Web of Science, Embase, or other bibliographic databases to improve coverage of multidisciplinary, regional, and non-indexed publication venues. Third, integration of citation networks, funding information, institutional affiliations, and collaboration networks could provide a more multidimensional view of research influence, infrastructure, and knowledge diffusion. Finally, external validation in other surgical specialties will be necessary to determine how well the workflow performs across fields with different journals, terminology, publication practices, and thematic structures.

Ultimately, this study highlights how computational intelligence can enhance the way surgical research itself is studied and steered [4,47,48]. Just as AI reshapes diagnostics and intraoperative planning, it can also elucidate the meta-dynamics of scientific evolution [49]. However, realizing this potential requires overcoming the structural and cultural barriers that currently limit AI integration within surgical practice and research systems [49]. Addressing these limitations—through standardized data architectures, interdisciplinary training, and ethical governance—will be essential for translating algorithmic insight into sustainable scientific progress [50]. By forecasting not only the magnitude but also the directionality of scholarly advancement, this framework offers a practical approach for summarizing publication patterns and supporting cautious, data-informed research planning. As surgical collaboration becomes increasingly global, AI-driven foresight may prove fundamental to sustaining innovation that is both inclusive and enduring across reconstruc-tive and microsurgical science.

## 6. Limitations

This study has several limitations. First, although the AI-assisted forecasting framework enables rapid and reproducible analysis, its performance depends on the completeness and consistency of PubMed metadata. Articles with missing abstracts or incomplete affiliation data may have been excluded, introducing minor selection bias. Second, microsurgery-related publications were identified using a predefined keyword taxonomy. While this ensured thematic specificity, emerging or unconventional terminology may have been underrepresented. We mitigated this through iterative synonym refinement and manual validation on stratified samples, but limited false exclusions may persist. Future incorporation of machine learning–based semantic classification could further enhance contextual sensitivity.

Third, journal selection and PubMed indexing impose an important scope constraint. Although the 20 journals were selected *a priori* to represent the principal specialties in which microsurgical techniques are practiced and reported, the sampling frame was not intended to constitute an exhaustive census of all microsurgery-related publications. Articles published in multidisciplinary, oncologic, transplantation, regional, non–PubMed-indexed journals, or conference proceedings may therefore have been missed. To improve transparency and reproducibility, we provide the complete predefined journal list in Table S1, encompassing both core microsurgery and reconstructive surgery journals and adjacent specialty journals. Accordingly, the reported findings and projections should be interpreted as trends within this predefined journal sampling frame rather than as a complete representation of the entire global microsurgery literature.

Fourth, the forecasting models were derived from historical publication trajectories and there-fore provide conditional projections rather than validated predictions of future scientific activity. The annual time series includes only 15 observations, which limits the precision of model selection and uncertainty estimates. Although multiple forecasting approaches were compared, 95% confidence intervals were reported, and temporal holdout and rolling-origin validation were added to assess short-horizon predictive stability, these analyses cannot eliminate the uncertainty inherent in extrapolating publication trends through 2030. The models assume that the underlying temporal structure observed from 2010 to 2024 will remain sufficiently stable over the projection period. They cannot account for future disruptions or structural changes, including shifts in funding priorities, research policy, geopolitical conditions, journal indexing, publication practices, workforce composition, or major technological breakthroughs. Moreover, publication volume should be interpreted as a measure of scholarly activity rather than a direct indicator of scientific quality, innovation, clinical significance, or future leadership. Accordingly, projections through 2030 rep-resent scenario-based estimates under continuation of observed 2010–2024 publication trends and should not be interpreted as deterministic forecasts of the future direction or impact of micro-surgical science. Fifth, first authorship was used as a proxy for national research capacity, which may not fully reflect collaborative or mentorship dynamics underlying innovation [25]. Alternative authorship-based sensitivity analyses remain an important direction for future work.

Country-level estimates were constrained by sparse publication activity in several regions and income groups. Although all countries with verifiable first-author affiliation data were retained in descriptive and income-group analyses, individual national projections for low-output countries may be unstable because of isolated publications, zero-count years, and limited temporal signal. Therefore, country-specific projections should be interpreted as exploratory, particularly for countries with low cumulative output. The absence of a stable country-level trajectory should not be interpreted as an absence of microsurgical research capacity or clinical activity.

This study should also be interpreted as a descriptive and forecasting analysis rather than a causal investigation. The observed relationships between publication output, geography, income classification, authorship patterns, and thematic growth represent associations within the indexed literature and should not be interpreted as causal effects. For example, increases in microsurgery publications from specific countries or thematic domains may coincide with changes in research infrastructure, funding priorities, training capacity, technological adoption, or publication practices, but the present design cannot determine whether any of these factors caused the observed trends. Distinguishing association from causation requires explicit causal assumptions and dedicated causal inference methods, which were outside the scope of this study [51]. Accordingly, all projections should be interpreted as structured estimates based on historical publication patterns rather than causal claims about future scientific productivity, innovation, or leadership.

An additional consideration is that each stage of the workflow may introduce distinct sources of bias. PubMed-only data collection may underrepresent microsurgery-related scholarship published in non-indexed journals, regional journals, conference proceedings, or multidisciplinary outlets not included in the selected journal set. The article classification strategy may also introduce mis-classification bias, particularly for studies using nonstandard terminology, reporting microsurgical techniques indirectly, or spanning multiple reconstructive domains. Finally, the forecasting models assume that historical publication trajectories provide a reasonable basis for short-term projection; they cannot incorporate unpredictable changes in funding priorities, institutional capacity, geopolitical conditions, publication practices, or disruptive technological advances. For these reasons, the projected trends should be interpreted as structured estimates of publication activity under observed historical conditions, rather than definitive predictions of future scientific leadership, innovation, or clinical impact.

Finally, although microsurgery served as the exemplar domain, the framework was intentionally designed to be discipline-agnostic and extensible. We enhanced generalizability through modular data ingestion, standardized normalization, cross-validated model calibration, and temporal robustness testing. External validation across additional surgical fields and integration with citation networks, funding data, and clinical outcomes will further strengthen transportability. These ex-tensions represent the natural evolution of a scalable system rather than limitations of its core design.

## 7. Conclusion

This study presents an AI-assisted bibliometric workflow for characterizing and projecting micro-surgical publication trends. By integrating automated PubMed metadata extraction, rule-based contextual keyword classification, human-reviewed validation, and conventional statistical forecasting, the workflow provides a reproducible approach for assessing publication activity across time, geography, authorship, and thematic domains. In this PubMed-derived sample of 20 microsurgery-relevant journals, annual publication output increased from 2010 to 2024, with exploratory projections suggesting continued growth through 2030 under continuation of observed historical pat-terns. These projections should be interpreted as conditional, trend-based estimates rather than predictive evidence of future scientific activity, innovation, research leadership, or clinical impact. Within these limitations, the workflow may help surgeons, clinician-scientists, and interdisciplinary research teams summarize evolving publication patterns, identify areas of increasing scholarly activity, and support cautious, data-informed research planning in microsurgery.

## Supporting information

S1 Appendix Supplementary tables and figures supporting the analysis, including the included journal list, additional country-level projections, model outputs, and supplementary forecasting results. (PDF)
