# Supplementary material for: AI-assisted forecasting in microsurgery: A dual-component framework for global publication trends
Source: PLoS One. 2026 Aug 28;21(8):e0357186. doi: 10.1371/journal.pone.0357186 (PMC13524238; doi:10.1371/journal.pone.0357186)
Supplement: Supplementary file 1 [file pone.0357186.s001.pdf]

8 Supplementary Appendix

Table S1: Journals Included in the Expanded Microsurgery Dataset

No.	Journal Name
1	Plastic and Reconstructive Surgery (PRS)
2	Plastic and Reconstructive Surgery – Global Open (PRSGO)
3	Journal of Plastic, Reconstructive & Aesthetic Surgery (JPRAS)
4	JPRAS Open
5	Annals of Plastic Surgery
6	Microsurgery
7	Journal of Reconstructive Microsurgery
8	Journal of Hand and Microsurgery
9	Journal of Hand Surgery (American Volume)
10	Journal of Hand Surgery (European Volume)
11	Injury
12	Clinics in Plastic Surgery
13	ANZ Journal of Surgery
14	Facial Plastic Surgery
15	Archives of Orthopaedic and Trauma Surgery
16	Journal of Craniofacial Surgery
17	Otolaryngologic Clinics of North America
18	Current Opinion in Otolaryngology & Head and Neck Surgery
19	Head & Neck
20	Archives of Otolaryngology–Head and Neck Surgery

Notes: This table lists the 20 journals included in the expanded microsurgery dataset. Selection was based on (i) consistent PubMed indexing, (ii) relevance to reconstructive and microsurgical practice, and (iii) stable publication volume across the 2010–2024 study window. The journal set captures both core microsurgery outlets and peripheral specialties contributing to microsurgical research output.

Table S2: Productivity Forecasts

Country	2024 Prod	2030 Pred	Growth/Year	Change	% Change
Switzerland	3.73	3.75	0.1678	0.02	0.5%
Japan	2.70	3.41	0.1077	0.71	26.4%
China	2.37	2.79	0.0758	0.42	17.8%
Egypt	1.77	2.74	0.1085	0.97	55.1%
Korea	1.90	2.36	0.0765	0.46	17.8%
Belgium	1.03	2.32	0.0796	1.29	125.2%
USA	1.95	2.31	0.0907	0.36	18.4%
Spain	1.64	2.14	0.0800	0.50	30.3%
Germany	1.75	2.06	0.0414	0.32	18.1%
South Korea	1.74	1.91	0.0334	0.17	10.0%

Notes: Productivity values represent annual per-capita research output (publications per million population) based on validated microsurgery records from 2010–2024. Forecasts reflect exponential-smoothing models projecting trends through 2030. “Growth/Year” denotes the average annual increase in productivity, while “Change” and “% Change” indicate absolute and proportional shifts from 2024 to 2030. Countries shown are the ten most productive or fastest-growing research ecosystems in the dataset.

Table S3: Author Growth Forecasts

Country	2024 Authors	2030 Pred	Growth/Year	Total Growth	% Growth
USA	2424	3336	149.46	912	37.7%
China	677	964	43.21	287	42.5%
United Kingdom	551	742	31.69	191	34.7%
Japan	445	619	28.45	174	39.2%
Taiwan	288	410	17.67	122	42.5%
Turkey	278	390	15.54	112	40.3%
South Korea	273	387	16.95	114	41.8%
Germany	275	380	16.76	105	38.5%
Australia	252	340	14.73	88	35.0%
Italy	243	337	15.20	94	38.9%

Notes: This table summarizes the projected growth of first authors in the top ten contributing countries from 2024 to 2030. Forecasts are derived from exponential-smoothing models applied to annualized authorship counts (2010–2024). "Growth/Year" reflects the estimated yearly increase in unique first authors, while "Total Growth" and "% Growth" quantify cumulative changes over the forecast horizon. These projections highlight sustained expansion of human capital in aesthetic research, with particularly strong relative increases anticipated in China, Taiwan, South Korea, and Turkey.

Table S4: Research Intensity Forecasts

Country	2024 Index	2030 Pred	Growth/Year	Change	% Change
Switzerland	8.09	8.13	0.364	0.04	0.5%
Taiwan	3.04	3.35	-0.016	0.31	10.3%
USA	2.23	2.64	0.067	0.41	18.4%
Japan	2.05	2.59	0.082	0.54	26.4%
Singapore	1.97	2.23	0.058	0.26	13.2%
Australia	2.23	2.22	0.040	-0.02	-0.8%
Italy	1.85	2.17	0.057	0.33	17.8%
South Korea	1.64	1.80	0.013	0.16	10.0%
Netherlands	1.85	1.72	0.138	-0.13	-7.0%
Belgium	0.75	1.70	0.058	0.94	125.2%

Notes: This table reports forecasted changes in national research intensity, defined as the ratio of annual publications to unique first authors. Predictions are based on exponential-smoothing models applied to 2010–2024 intensity trends. "Growth/Year" reflects the expected yearly change in the intensity index, while "Change" and "% Change" represent the cumulative deviation from baseline by 2030. The projections highlight substantial heterogeneity across countries, with strong relative gains anticipated in Belgium, Japan, and the United States, while a minority of nations show stable or modestly declining trajectories.

Figure S1: Research Intensity Index Forecasting

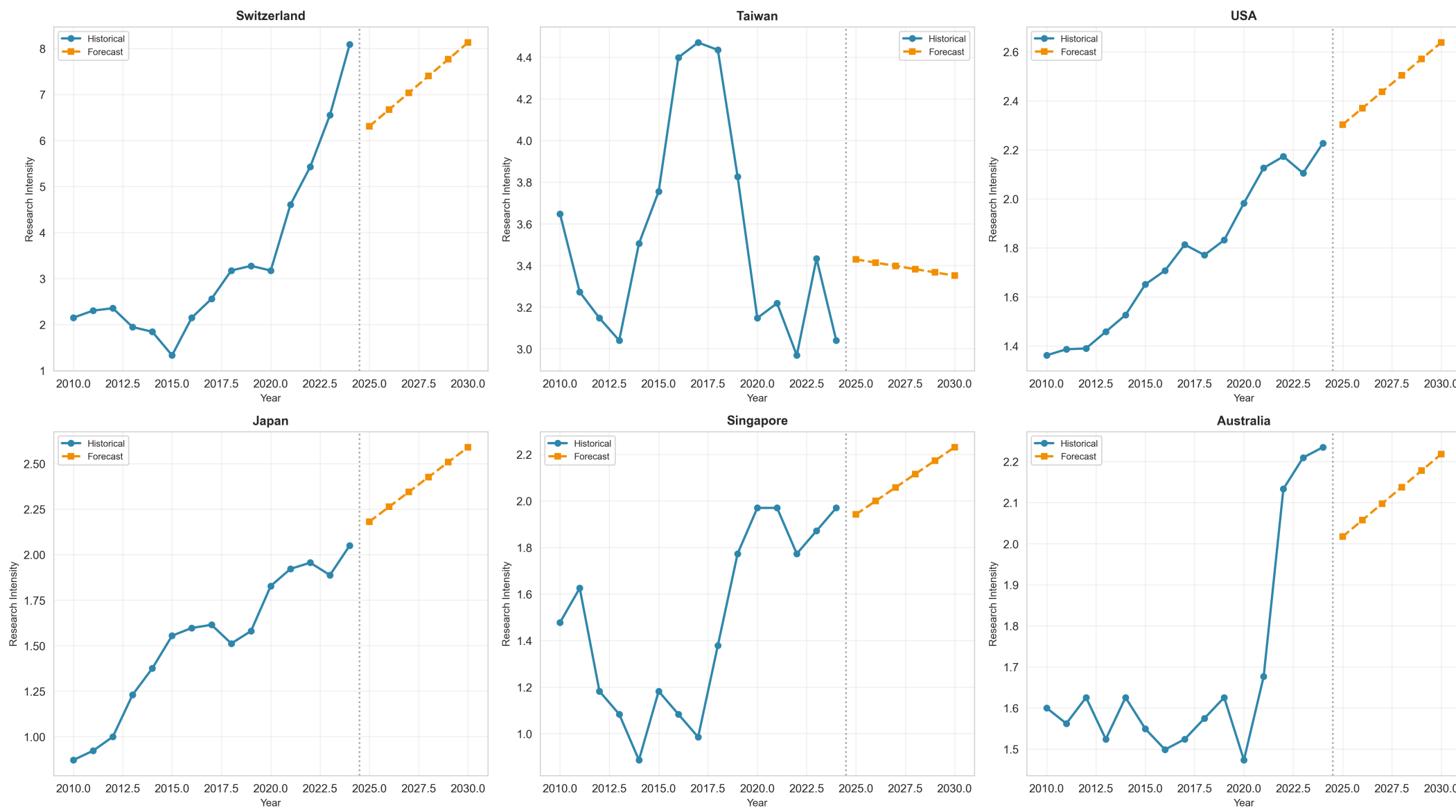

Notes: The Research Intensity Index is a compound metric combining productivity (publications per author) and population-adjusted output (publications per 100,000 population). This index identifies true research hubs—countries where high per-capita research output combines with high per-author productivity to create concentrated research ecosystems.

Figure S2: Top 6 countries: Growth Predictions (2025-2030)

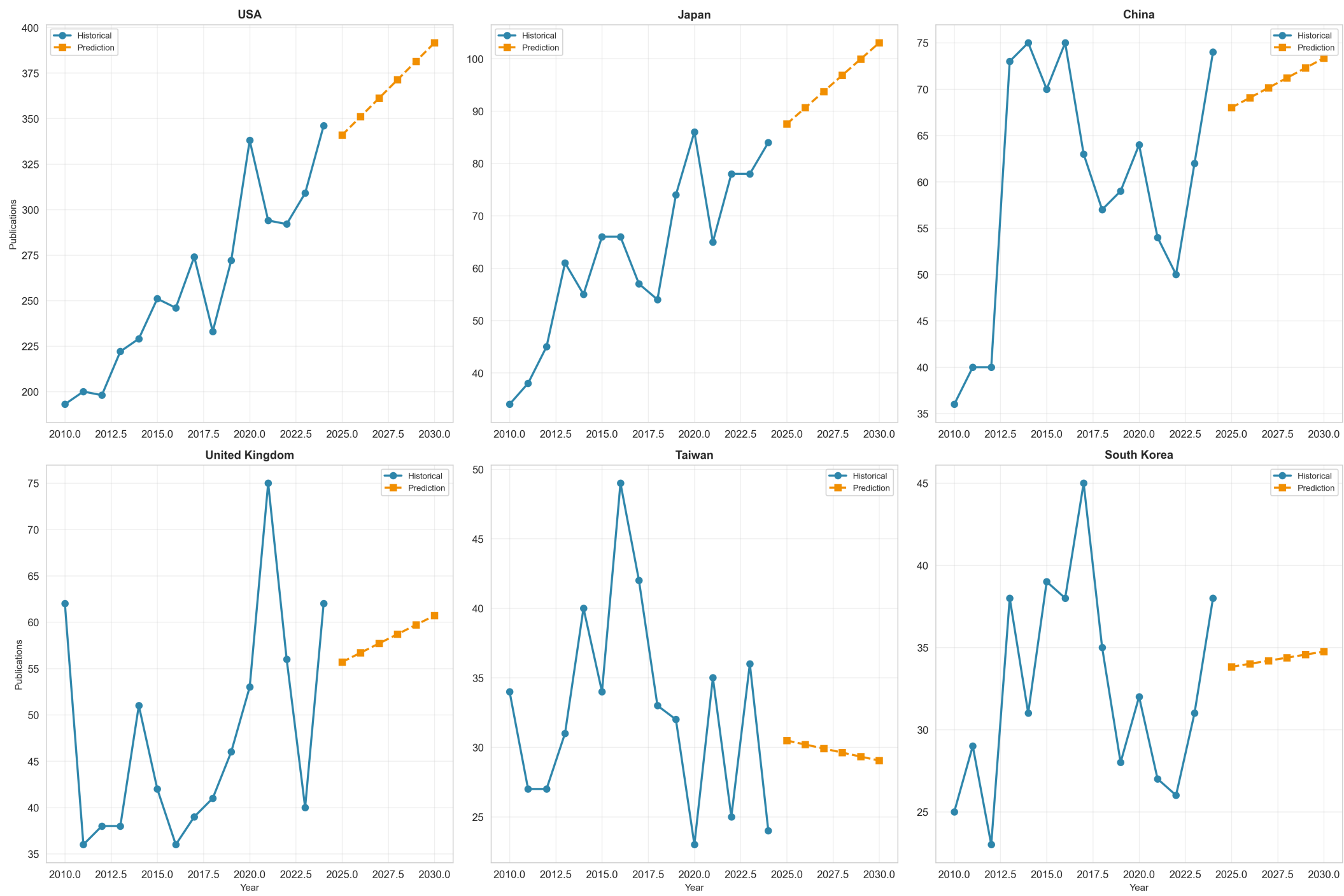

Notes: Individual country projections reveal varying growth trajectories. The USA maintains strong growth (+10.1 publications/year), while established leaders like Japan and China show moderate expansion. Notably, Taiwan shows a slight declining trend (-0.3/year), suggesting potential saturation or shifting research priorities. These country-specific forecasts help identify emerging research hubs and potential shifts in the global microsurgery research landscape.

Figure S3: Author Growth Forecasting for Taiwan (2010–2030)

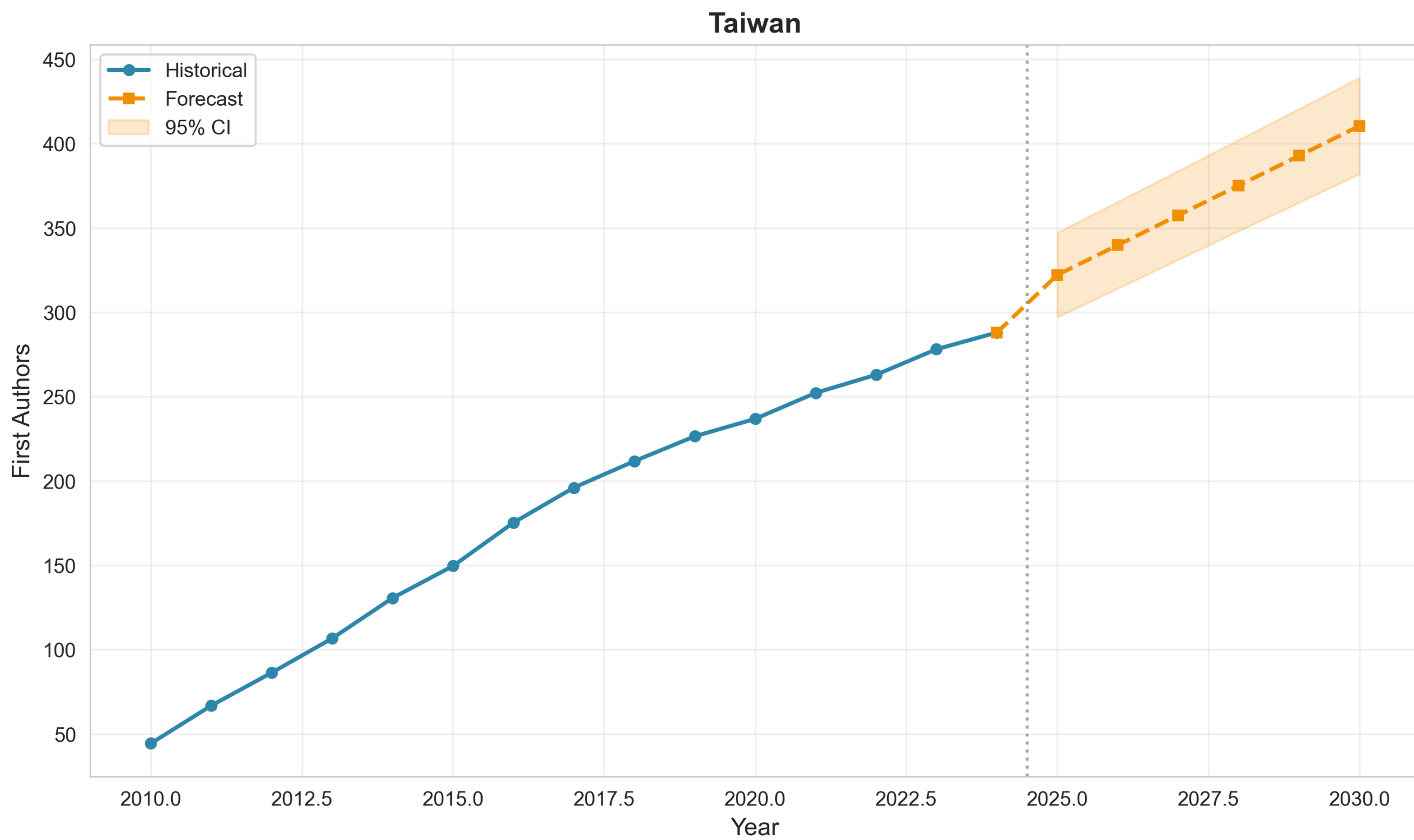

Notes: This visualization illustrates the historical and forecasted trajectory of microsurgery first author accumulation for Taiwan from 2010 through 2030. Estimated cumulative first authors from 2010-2024 (solid blue line with circular markers) represent observed data, while projections from 2025-2030 (orange dashed line) were generated using the Linear model. The vertical dashed line denotes the transition point between observed data and forecasted estimates. The shaded area represents the 95% CI. The model predicts moderate continued growth in Taiwan's microsurgery author base, reflecting a productive research community with steady expansion in the number of contributing researchers.

Figure S4: Author Growth Forecasting for Turkey (2010–2030)

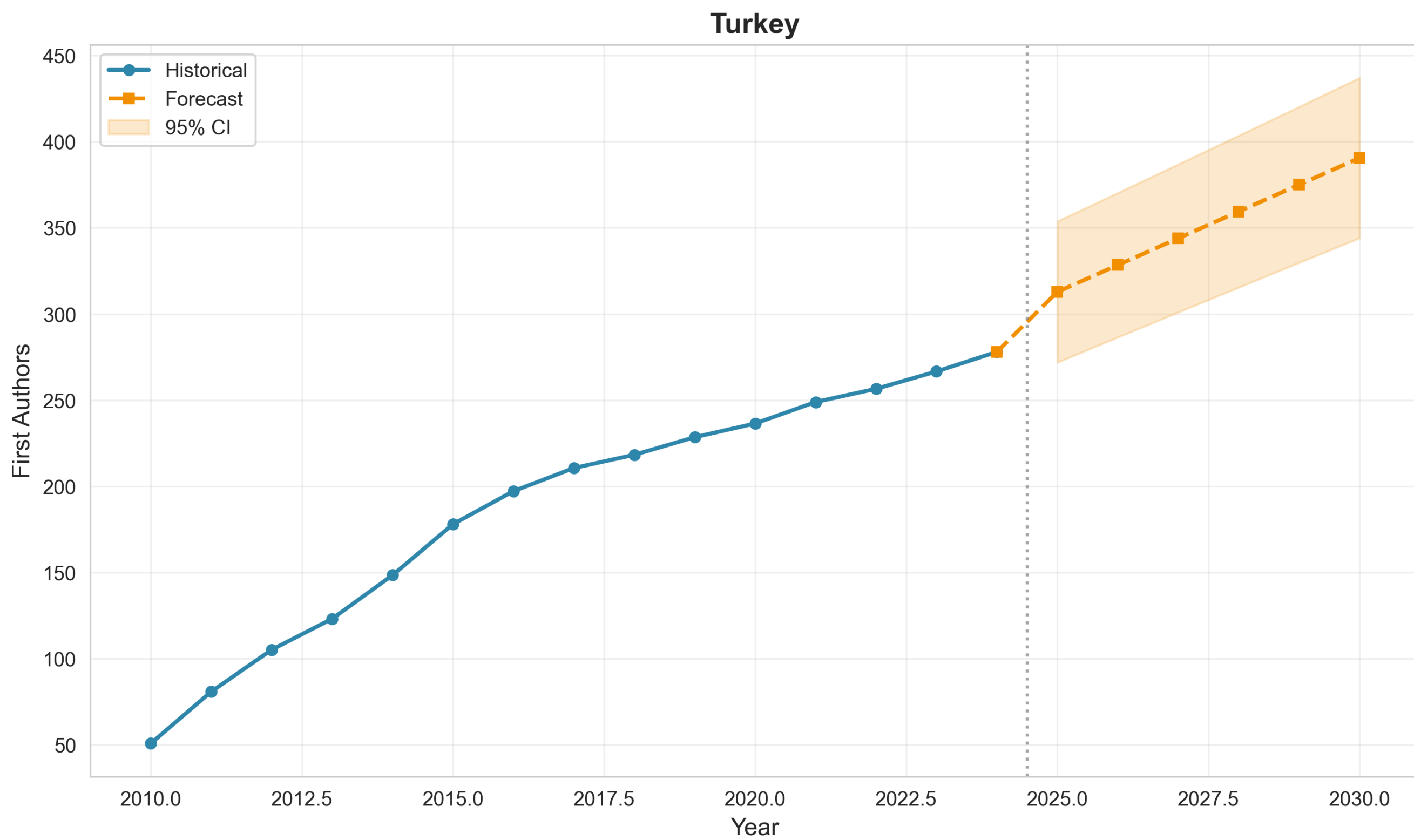

Notes: This visualization illustrates the historical and forecasted trajectory of microsurgery first author accumulation for Turkey from 2010 through 2030. Estimated cumulative first authors from 2010-2024 (solid blue line with circular markers) represent observed data, while projections from 2025-2030 (orange dashed line) were generated using the Linear model. The vertical dashed line denotes the transition point between observed data and forecasted estimates. The shaded area represents the 95% CI. The model predicts moderate growth in Turkey's microsurgery author base, reflecting an emerging research community with increasing participation and a growing number of first-time contributors to the microsurgery literature.

Figure S5: Author Growth Forecasting for Japan (2010–2030)

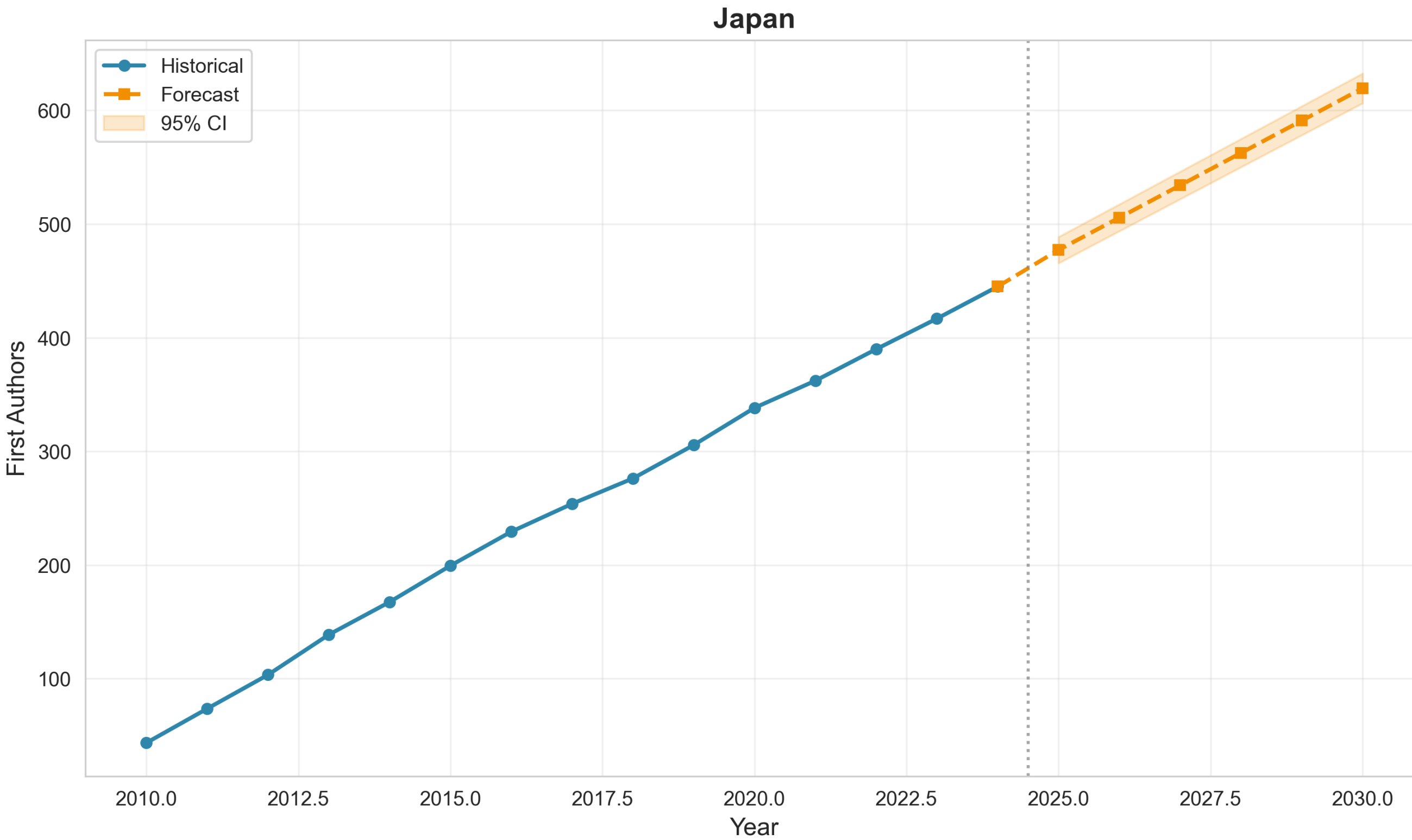

Notes: This visualization illustrates the historical and forecasted trajectory of microsurgery first author accumulation for Japan from 2010 through 2030. Estimated cumulative first authors from 2010-2024 (solid blue line with circular markers) represent observed data, while projections from 2025-2030 (orange dashed line) were generated using the Linear model. The vertical dashed line denotes the transition point between observed data and forecasted estimates. The shaded area represents the 95% CI. The model predicts continued steady growth in Japan's microsurgery author base, reflecting a mature research ecosystem with a consistently growing pool of active researchers contributing to the field.
