# Supplementary material for: AI-assisted forecasting in microsurgery: A dual-component framework for global publication trends
Source: PLoS One. 2026 Aug 28;21(8):e0357186. doi: 10.1371/journal.pone.0357186 (PMC13524238; doi:10.1371/journal.pone.0357186)
Supplement: Supplementary file 3 [file pone.0357186.s003.pdf]

August 8, 2026

Revision of "AI-Assisted Forecasting in Microsurgery: A Dual-Component Framework for Global Publication Trends"

PONE-D-26-26132

Responses to Comments

Thank you very much for the opportunity to revise our paper. Please find below our responses to the reviewers' comments. We reproduce each comment in blue, followed by our response.

Reviewer 1 Comments:

1. The AI novelty remains overstated: Although the authors now describe the framework more transparently, the system is fundamentally a keyword-based contextual classifier combined with conventional statistical forecasting models (Linear Regression, Polynomial Regression, ARIMA, and Holt's Exponential Smoothing). It should be made clearer throughout the manuscript that this is an AI-assisted bibliometric workflow rather than a novel AI methodology. Several statements in the Abstract, Discussion, and Conclusion still overstate the AI contribution.

Reply: We thank the reviewer for this important clarification. We agree that the manuscript still contained wording that could overstate the artificial intelligence contribution. We have revised the Abstract, Discussion, Conclusion, keywords, and Methods terminology to make clear that this study does not present a novel AI architecture, supervised machine-learning classifier, large language model, or autonomous predictive system. Rather, the study presents an AI-assisted bibliometric workflow that combines automated PubMed metadata extraction, keyword-based contextual text classification, human-reviewed validation, and conventional statistical forecasting.

Specifically, we replaced language such as "AI-powered," "AI-based system," "semantic intelligence," "predictive insight," "methodological leap," and "intelligent text classification" with more precise terms such as "AI-assisted bibliometric workflow," "automated literature analytics workflow," "contextual keyword-based classification," and "statistical projection model." We also removed "Machine learning" from the keywords to avoid implying that a machine-learning model was trained. These revisions preserve the clinical and interdisciplinary value of the workflow while accurately distinguishing automated literature processing from methodological innovation in artifi-

cial intelligence.

Revised Abstract Conclusion:

"This AI-assisted bibliometric workflow provides a scalable and reproducible approach for assessing microsurgical publication activity across time, geography, and thematic domains. Rather than offering a novel AI methodology or deterministic prediction model, the workflow combines automated metadata extraction, contextual keyword-based classification, human-reviewed validation, and transparent statistical forecasting. For surgeons, clinician-scientists, and interdisciplinary research teams, this approach may help summarize historical publication patterns, generate conditional projections, and support research planning within the limits of publication-count data."

Revised Discussion text:

"This study demonstrates how an AI-assisted bibliometric workflow can support large-scale assessment of microsurgical publication activity. By integrating automated metadata extraction, contextual keyword-based classification, human-reviewed validation, and conventional statistical forecasting, the framework provides a reproducible approach for examining historical and projected publication trends across time, geography, authorship, and research domains. This clinically oriented workflow offers surgeons, clinician-scientists, and interdisciplinary research teams a practical method for summarizing evolving research patterns and identifying areas of increasing scholarly activity within microsurgery [1]."

Revised Conclusion:

" This study presents an AI-assisted bibliometric workflow for characterizing and projecting microsurgical publication trends. By integrating automated PubMed metadata extraction, contextual keyword-based classification, human-reviewed validation, and conventional statistical forecasting, the workflow enables reproducible assessment of publication activity across time, geography, authorship, and thematic domains. The resulting estimates should be interpreted as conditional projections under observed historical

trends rather than deterministic predictions of future scientific activity, innovation, or leadership. This approach may assist surgeons, clinician-scientists, and interdisciplinary research teams in summarizing research patterns, identifying areas of increasing scholarly activity, and informing future collaborative planning."

Revised Keywords:

" Artificial intelligence-assisted workflow; Text mining; Research forecasting; Publication trends; Microsurgery; Data science."

2. The contextual classifier is still rule-based rather than machine learning-based: The classification approach relies on a predefined keyword taxonomy with manual adjudication instead of a supervised or deep learning classifier. The manuscript should avoid implying semantic AI capabilities beyond what is actually implemented.

Reply: We thank the reviewer for this important clarification. We agree that the classification component is rule-based and should not be described in a way that implies supervised machine learning, deep learning, large language modeling, or autonomous semantic AI capabilities. We have revised the manuscript accordingly.

Throughout the Abstract, Methods, Results, Discussion, and Conclusion, we replaced terms such as "semantic intelligence," "AI classifier," "AI-based classification," and "contextual semantic modeling" with more precise language, including "rule-based contextual keyword classification," "predefined keyword taxonomy," "automated literature-processing workflow," and "human-reviewed adjudication." We also clarified that the classification algorithm applies an 86-term/stem taxonomy to article titles and abstracts using case-insensitive matching and wildcard/stem expansion, with ambiguous records adjudicated according to a title/abstract primary-focus rule.

These revisions make clear that the classifier is not a trained machine-learning model. Rather, the workflow uses automated metadata extraction, rule-based contextual keyword classification, human-reviewed validation, and conventional statistical forecasting to support reproducible assessment of microsurgical publication trends.

Revised Methods text:

> *"Article classification was performed using a rule-based contextual keyword classifier rather than a supervised machine-learning or deep-learning model. The classifier applied a predefined microsurgery taxonomy to article titles and abstracts using case-insensitive matching with wildcard and stem expansion where appropriate. Articles with incidental keyword matches were classified according to primary study focus rather than keyword presence alone, and ambiguous records, records with limited abstracts, and records spanning multiple reconstructive domains were adjudicated using a title/abstract primary-focus rule."*

Revised Discussion text:

> *"The classification component should be interpreted as a transparent, rule-based contextual keyword approach rather than a trained semantic AI model. Its value lies in providing a reproducible screening and structuring method for a large PubMed-derived literature corpus, with human-reviewed validation used to assess classification performance and characterize misclassification patterns."*

3. Forecasting conclusions remain stronger than the available evidence supports: The study is based on only fifteen annual observations (2010–2024). Although additional validation has been included, forecasts through 2030 should continue to be presented cautiously as exploratory projections rather than predictive evidence.

Reply: We thank the reviewer for this important point. We agree that the 2025–2030 estimates should be interpreted cautiously because the forecasting analysis is based on only 15 annual observations. Although we added temporal holdout validation, rolling-origin validation, residual diagnostics, and expanded limitations in the previous revision, we recognize that some wording in the Discussion, and Conclusion may still have implied a stronger predictive interpretation than intended.

We have therefore further revised the manuscript to describe all 2030 estimates as exploratory, conditional projections rather than predictive evidence. Specifically, we replaced deterministic language such as "predicts," "will retain," "will account for," "forecasting reveals," and "predictive insight" with more cautious terms such as "is projected to," "may," "conditional projections," "exploratory estimates," and "under continuation of observed historical trends." We also added explicit

cautionary language to the Discussion and Conclusion stating that the projections are intended to summarize plausible publication trajectories under historical assumptions, not to establish future scientific activity, research leadership, innovation, or impact.

Revised Discussion text:

> *"The 2025–2030 estimates should be interpreted in the context of the 15-year annual time series from which they were derived. Temporal validation supported short-horizon stability of the selected model within the observed dataset; however, projections beyond 2024 necessarily depend on the assumption that historical publication patterns continue over the forecast period. For this reason, the projected trajectories are best viewed as exploratory, trend-based estimates of scholarly activity rather than definitive forecasts of future scientific output, innovation, research leadership, or clinical impact."*

Revised Conclusion text:

> *"The resulting estimates should be interpreted as conditional projections under observed historical trends rather than deterministic predictions of future scientific activity, innovation, or leadership. This approach may assist surgeons, clinician-scientists, and interdisciplinary research teams in summarizing research patterns, identifying areas of increasing scholarly activity, and informing future collaborative planning."*

4. What are the main contributions of your research? Should be mentioned in Intrduction section and Contributions should be novel. the overall structure of the paper should also be defined at the end of introduction section.

Reply: We thank the reviewer for this helpful suggestion. We agree that the Introduction should more explicitly state the main contributions of the study and outline the structure of the manuscript. We have therefore revised the end of the Introduction to include a dedicated contribution paragraph and a brief paper-organization paragraph.

The revised contribution paragraph clarifies that the novelty of the study lies not in the development of a new AI architecture, but in the application of a reproducible AI-assisted bibliometric

workflow to microsurgery. Specifically, we now highlight four main contributions: construction of a large PubMed-derived microsurgery publication dataset, validation of a rule-based contextual keyword classifier against a human-reviewed reference standard, integration of comparative statistical forecasting with temporal validation, and evaluation of publication trends across geographic, economic, authorship, and thematic dimensions. We also added a final paragraph summarizing the organization of the manuscript.

> *"The main contribution of this study is the development and application of a reproducible, AI-assisted bibliometric workflow for microsurgery research assessment. First, we constructed a large PubMed-derived dataset of microsurgery-related publications across 20 journals using automated metadata extraction and rule-based contextual keyword classification. Second, we validated the classification process against a human-reviewed reference standard and characterized classification performance using agreement, Cohen's κ, sensitivity, specificity, predictive values, and false-positive and false-negative rates. Third, we integrated comparative statistical forecasting with temporal validation to generate exploratory projections of microsurgical publication activity through 2030. Fourth, we examined these trends across geographic, economic, authorship, and thematic dimensions, allowing the framework to identify areas of increasing scholarly activity and persistent disparities within the global microsurgery literature. Together, these contributions extend prior retrospective microsurgery bibliometric studies by providing a transparent and clinically interpretable workflow for summarizing historical patterns and generating cautious, trend-based projections."*

> *"The remainder of the paper is organized as follows. The Literature Review and Related Work section summarizes prior work in surgical bibliometrics, automated metadata extraction, text mining, and forecasting-based research assessment. The Methods section describes the journal sampling frame, PubMed metadata extraction, rule-based contextual classification strategy, validation process, thematic-domain construction, authorship-based human-capital definition, and statistical forecasting approach. The Results section presents the dataset construction process, classification performance, temporal publication trends, geographic and income-group distributions, model-validation results, authorship patterns, and projected thematic growth. The Discussion section in-*

terprets these findings in relation to prior microsurgery and surgical scientometric literature, outlines the clinical and research-planning implications of the workflow, and discusses the assumptions and limitations of exploratory publication forecasting. The Conclusion summarizes the main findings and the potential role of AI-assisted bibliometric workflows in supporting future microsurgery research assessment."

5. Many figure legends still contain detailed interpretation and discussion rather than concise figure descriptions. Interpretation should remain in the Results or Discussion sections.

Reply: We thank the reviewer for this helpful comment. We agree that several figure legends contained interpretive language that was more appropriate for the Results or Discussion sections. We have revised the figure legends and notes throughout the manuscript to make them more concise and descriptive. Specifically, we removed interpretive phrases related to research leadership, ecosystem maturity, emerging research hubs, saturation, productivity efficiency, and projected dominance. The revised legends now describe the data shown, the observed and projected time periods, the modeling approach, and the meaning of confidence intervals or visual markers. Interpretive discussion of these findings has been retained only in the Results and Discussion sections.

6. Despite previous comments, the figures and tables continue to be presented collectively after the references rather than being embedded near their first citation. This negatively affects readability and should be corrected according to the journal's formatting guidelines.

Reply: We thank the reviewer for this comment. We agree that placing all figures and tables collectively after the references reduced readability and made it more difficult to follow the Results section. We have corrected this in the revised manuscript.

All main-text figures and tables have now been moved from the end of the manuscript and embedded near their first citation in the relevant sections of the text. Specifically, the workflow figure and dataset construction table are placed near the Methods and early Results sections, the forecasting model comparison table is placed near the model-validation results, and the geographic, authorship, productivity, and thematic figures are positioned adjacent to the corresponding Results subsections. We also revised the figure legends to be concise and descriptive, with interpretation retained in the Results and Discussion sections.

These formatting changes were made to improve readability and align the manuscript with the journal's presentation requirements.

7. The conclusion claims that the framework "redefines" surgical bibliometrics and represents a major methodological advance. These claims should be moderated to better reflect the actual scope of the presented work.

Reply: We thank the reviewer for this helpful comment. We agree that the prior Conclusion contained language that overstated the scope and methodological novelty of the work. In particular, phrases such as “redefines surgical bibliometrics,” “methodological advance,” and “transforming how research ecosystems can be mapped and forecasted” could imply that the study introduces a fundamentally new AI methodology, whereas the manuscript presents an AI-assisted bibliometric workflow using automated metadata extraction, rule-based contextual keyword classification, human-reviewed validation, and conventional statistical forecasting.

We have revised the Conclusion to use more measured language. The revised text now emphasizes the practical and reproducible nature of the workflow, the exploratory nature of the projections, and the clinical relevance of summarizing microsurgical publication trends. We also removed claims suggesting that the framework represents a major methodological advance or predicts future innovation, leadership, or impact.

Revised Conclusion:

> *“This study presents an AI-assisted bibliometric workflow for characterizing and projecting microsurgical publication trends. By integrating automated PubMed metadata extraction, rule-based contextual keyword classification, human-reviewed validation, and conventional statistical forecasting, the workflow provides a reproducible approach for assessing publication activity across time, geography, authorship, and thematic domains. In this PubMed-derived sample of 20 microsurgery-relevant journals, annual publication output increased from 2010 to 2024, with exploratory projections suggesting continued growth through 2030 under continuation of observed historical patterns. These projections should be interpreted as conditional, trend-based estimates rather than predictive evidence of future scientific activity, innovation, research leadership, or clinical impact. Within these limitations, the workflow may help surgeons, clinician-scientists, and in-*

terdisciplinary research teams summarize evolving publication patterns, identify areas of increasing scholarly activity, and support cautious, data-informed research planning in microsurgery."

8. Some statements still employ promotional or subjective language, including terms such as "methodological leap," "redefines surgical bibliometrics," "unprecedented resolution," "strategic instrument," and "dynamic system." These expressions should be replaced with more objective scientific wording that is fully supported by the presented evidence.

Reply: We thank the reviewer for this helpful comment. We agree that several phrases in the manuscript were promotional or subjective and could overstate the contribution of the study. We have revised the manuscript throughout to use more objective scientific wording that better reflects the actual scope of the work.

Specifically, we removed or replaced phrases such as "methodological leap," "redefines surgical bibliometrics," "unprecedented resolution," "strategic instrument," "dynamic system," "AI-driven foresight," and similar language. These terms have been replaced with more measured wording, including "reproducible workflow," "AI-assisted bibliometric workflow," "structured approach," "clinically interpretable method," "publication-trend assessment," and "exploratory projection framework." We also revised the Discussion and Conclusion to emphasize that the study provides a scalable approach for summarizing and projecting publication activity, rather than a novel AI methodology or definitive predictive system.

Examples of revised wording:

The phrase "methodological leap for surgical analytics" was replaced with "a reproducible approach for large-scale surgical publication analysis."

The phrase "redefines surgical bibliometrics" was replaced with "provides an AI-assisted bibliometric workflow for characterizing and projecting microsurgical publication trends."

The phrase "unprecedented resolution" was replaced with "across time, geography, authorship, and thematic domains."

The phrase "strategic instrument for anticipating change and directing growth" was

replaced with "a practical approach for summarizing publication patterns and supporting cautious, data-informed research planning."

The phrase "dynamic system for anticipating where and how surgical innovation will evolve" was replaced with "a structured approach for examining historical and projected publication trends under observed historical assumptions."

Revised Discussion text:

"This study demonstrates how an AI-assisted bibliometric workflow can support large-scale assessment of microsurgical publication activity. By integrating automated metadata extraction, rule-based contextual keyword classification, human-reviewed validation, and conventional statistical forecasting, the framework provides a reproducible approach for examining historical and projected publication trends across time, geography, authorship, and research domains. Applied to microsurgery, this workflow offers a clinically interpretable method for mapping the evolution of scholarly activity and identifying areas of increasing research attention within a complex interdisciplinary surgical field."

Revised Conclusion text:

"This study presents an AI-assisted bibliometric workflow for characterizing and projecting microsurgical publication trends. By integrating automated PubMed metadata extraction, rule-based contextual keyword classification, human-reviewed validation, and conventional statistical forecasting, the workflow provides a reproducible approach for assessing publication activity across time, geography, authorship, and thematic domains. In this PubMed-derived sample of 20 microsurgery-relevant journals, annual publication output increased from 2010 to 2024, with exploratory projections suggesting continued growth through 2030 under continuation of observed historical patterns. These projections should be interpreted as conditional, trend-based estimates rather than predictive evidence of future scientific activity, innovation, research leadership, or clinical impact. Within these limitations, the workflow may help surgeons, clinician-scientists, and interdisciplinary research teams summarize evolving publication patterns, identify areas of increasing scholarly activity, and support cautious, data-informed research planning in microsurgery."

9. The authors claim that the proposed framework is transferable to other surgical specialties. This statement should be qualified by briefly discussing what modifications would be required (e.g., journal selection, keyword taxonomy, validation process) before applying the framework to other medical domains.

Reply: We thank the reviewer for this helpful comment. We agree that the transferability of the workflow should be qualified rather than presented as automatic. In the revised manuscript, we clarify that the workflow is potentially adaptable to other surgical or medical domains, but only after field-specific modification of the journal sampling frame, keyword taxonomy, thematic categories, and validation process.

We also now state that the workflow has already been applied by our group in related publication-trend studies in aesthetic surgery and craniofacial surgery, but we emphasize that each application required domain-specific adaptation. For example, a study of aesthetic surgery requires journals and keywords related to aesthetic procedures, cosmetic surgery, and subspecialty-specific terminology, whereas a craniofacial application requires modification of the sampling frame and taxonomy to capture craniosynostosis, facial trauma, cleft surgery, orthognathic surgery, and related craniofacial topics. Therefore, the framework should be considered a transferable workflow structure, not a plug-and-play classifier or universally valid forecasting model.

Added Discussion text:

> *"The workflow may be adaptable to other surgical and medical domains, but such transferability requires field-specific modification rather than direct reuse of the microsurgery classifier. The journal sampling frame would need to be redefined to capture the relevant specialty literature, and the keyword taxonomy would need to be reconstructed around the procedures, diagnoses, technologies, and terminology of the target field. Thematic domains would also require specialty-specific input to ensure that categories are clinically meaningful and sufficiently frequent for temporal modeling. Finally, the classification process would require renewed human-reviewed validation, including assessment of agreement, sensitivity, specificity, false-positive and false-negative patterns, and performance across journals and years."*

> *"This domain-specific adaptation is consistent with our prior applications of related*

publication-trend workflows in aesthetic surgery and craniofacial surgery [2, 3]. In each setting, the analytic structure remained similar, but the journal selection, terminology, thematic grouping, and validation process were tailored to the clinical field under study. Accordingly, the present framework should be interpreted as a transferable workflow structure for AI-assisted bibliometric assessment, not as a universal classifier or forecasting model that can be applied unchanged across specialties."

10. The manuscript would benefit from one final round of professional English language editing to correct minor grammatical inconsistencies, improve sentence flow, and ensure consistent formatting throughout the manuscript.

Reply: We thank the reviewer for this comment. We have carefully revised the manuscript for grammar, sentence flow, terminology, and formatting consistency. In particular, we standardized the use of terms such as "AI-assisted bibliometric workflow," "rule-based contextual keyword classification," "statistical forecasting," "exploratory projections," and "conditional estimates." We also reviewed the Abstract, Discussion, Conclusion, figure legends, and table notes to improve clarity and ensure that the language remains objective and consistent with the scope of the study.

The manuscript would benefit from a brief "Future Work" paragraph suggesting possible enhancements, such as incorporation of transformer-based NLP models or large language models for semantic classification, inclusion of additional bibliographic databases such as Scopus and Web of Science, integration of citation networks, funding information, and collaboration networks, and external validation in other surgical specialties.

Reply: We thank the reviewer for this helpful suggestion. We agree that a brief discussion of future work would improve the manuscript by clarifying how the workflow could be strengthened in subsequent studies. We have added a new paragraph near the end of the Discussion outlining several potential enhancements. These include incorporation of transformer-based natural language processing models or large language models for more flexible semantic classification, expansion beyond PubMed to additional bibliographic databases such as Scopus and Web of Science, integration of citation networks, funding information, institutional affiliations, and collaboration networks, and external validation across other surgical specialties. We also emphasize that these extensions would require domain-specific validation before implementation.

"Future work may extend this workflow in several directions. First, transformer-based natural language processing models or large language models could be incorporated to improve semantic classification, particularly for articles using indirect, emerging, or nonstandard terminology. Such models would require careful training, calibration, and human-reviewed validation to ensure that improved recall does not come at the expense of interpretability or specificity. Second, future studies could expand the data source beyond PubMed by incorporating Scopus, Web of Science, Embase, or other bibliographic databases to improve coverage of multidisciplinary, regional, and non-indexed publication venues. Third, integration of citation networks, funding information, institutional affiliations, and collaboration networks could provide a more multidimensional view of research influence, infrastructure, and knowledge diffusion. Finally, external validation in other surgical specialties will be necessary to determine how well the workflow performs across fields with different journals, terminology, publication practices, and thematic structures."

Reviewer 2 Comments:

Thank you for your careful revision. The manuscript is much stronger than the previous version, and the revisions have addressed the main concerns raised during the first round of review. The methodological framework is now presented more clearly. In particular, the distinction between the automated literature processing and the statistical forecasting components is easier to follow, and the revised terminology more accurately reflects the scope of the work. The additional methodological detail also improves the transparency of the study and makes the workflow easier to reproduce.

The expanded validation strengthens the manuscript. The temporal validation, residual diagnostics, and more detailed reporting of the classification performance provide a clearer picture of how the framework performs in practice. I also found the discussion of the classification errors useful, as it helps readers understand the limitations of the approach rather than focusing only on its overall performance.

The interpretation of the findings is now more balanced. The manuscript consistently presents the forecasts as projections based on historical trends rather than as predictions of future research activity, and the limitations of the study are acknowledged appropriately. The conclusions are supported by the analyses presented.

Overall, I believe the study is technically sound and meets the publication criteria. The methods are described in sufficient detail to allow reproduction, and the conclusions are supported by the data. I have only a few minor suggestions before publication.

Reply: We thank the reviewer for the thoughtful reassessment of our revised manuscript and for the positive comments regarding the clarity of the methodological framework, expanded validation, classification error analysis, and more balanced interpretation of the findings. We are grateful that the revisions addressed the main concerns raised during the first round of review.

We have carefully addressed the remaining minor comments. Specifically, we reviewed the numbering of all figures, tables, supplementary figures, supplementary tables, and in-text cross-references to ensure consistency throughout the manuscript. We also performed a final proofread to correct remaining typographical, grammatical, and formatting inconsistencies.

Minor Comment 1: Please check the numbering of the figures, tables, and supplementary material to ensure that all cross-references are consistent throughout the manuscript.

Reply: We thank the reviewer for this helpful comment. We have reviewed the numbering of all main-text figures, tables, supplementary figures, supplementary tables, and corresponding in-text citations. Cross-references were corrected where needed to ensure that each figure, table, and supplementary item is cited consistently and in the appropriate order throughout the manuscript.

Minor Comment 2: A final proofread would help eliminate the small number of typographical and grammatical inconsistencies that remain.

Reply: We thank the reviewer for this suggestion. We have completed a final proofread of the manuscript to correct minor typographical, grammatical, punctuation, and formatting inconsistencies. We also reviewed terminology for consistency, particularly the use of "AI-assisted bibliometric workflow," "rule-based contextual keyword classification," "statistical forecasting," "exploratory projections," and "conditional estimates."

Reviewer 3 Comments:

I have reviewed the authors responses and the revised manuscript and believe that the manuscript has been substantially improved and now meets the standards for publication.

Reply: We thank the reviewer for the careful reassessment of our revised manuscript and for the positive evaluation. We are grateful that the revisions addressed the prior concerns and improved the clarity, methodological transparency, and overall presentation of the study.

References

[1] Miltos K Lazarides, Irene-Zacharo Lazaridou, and Nikolaos Papanas. Bibliometric analysis: bridging informatics with science. *The International Journal of Lower Extremity Wounds*, 24(3):515–517, 2025.

[2] Georgios Karamitros, Georgios Bouloukakis, Emmanuel Giannas, Gregory A Lamaris, Wesley P Thayer, Galen Perdikis, William C Lineaweaver, and Patrick Assi. Predicting the future of aesthetic surgery: An artificial intelligence framework for global publication forecasting. *Aesthetic Surgery Journal*, 46(7):773–785, 2026.

[3] Georgios Karamitros, Georgios Bouloukakis, Gregory A. Lamaris, Wesley P. Thayer, Galen Perdikis, Feng Zhang, Richard J. Redett, Robin Yang, Matthew Pontell, and William C. Lineaweaver. Predicting the future of craniofacial surgery: Ai-enabled forecasting of craniofacial surgery research and future clinical priorities. *Journal of Craniofacial Surgery*, 2026. In press.
