# Supplementary material for: AI-assisted forecasting in microsurgery: A dual-component framework for global publication trends
Source: PLoS One. 2026 Aug 28;21(8):e0357186. doi: 10.1371/journal.pone.0357186 (PMC13524238; doi:10.1371/journal.pone.0357186)
Supplement: Supplementary file 4 [file pone.0357186.s002.pdf]

July 17, 2026

Revision of "AI-Assisted Forecasting in Microsurgery: A Dual-Component Framework for Global Publication Trends"

PONE-D-26-26132

Responses to Comments

Thank you very much for the opportunity to revise our paper. Please find below our responses to the reviewers' comments. We reproduce each comment in blue, followed by our response.

Reviewer 1 Comments:

1. The abstract is poorly structured and does not adequately summarize the study. It should clearly describe: the AI/ML models used, the forecasting methods employed, Dataset size, Evaluation metrics, Key quantitative findings and conclusions.

Reply: We thank the reviewer for this helpful comment. We agree that the original abstract did not sufficiently summarize the methodological and quantitative components of the study. We have revised the abstract to provide a clearer structured overview of the AI-assisted workflow, including the automated PubMed extraction process, contextual text-mining classification, forecasting models compared, dataset size, validation approach, evaluation metrics, principal quantitative findings, and clinical/research implications. We also moderated the language to clarify that the study provides bibliometric projections based on observed historical trends rather than deterministic predictions. Importantly, the revised abstract preserves the manuscript's intended focus as an interdisciplinary medical research framework for surgeons and clinician-scientists, rather than converting it into a purely technical machine-learning paper.

Revised manuscript Abstract:

Abstract

Background:

Artificial intelligence (AI)-assisted approaches may allow surgical research trends to be

analyzed at scale and projected over time. However, their use in forecasting the evolution of microsurgical scholarship remains limited. This study developed an AI-assisted bibliometric framework to characterize and project global clinical and experimental microsurgery publication trends.

Methods:

PubMed metadata from 20 microsurgery-relevant surgical journals were extracted for 2010–2024 using an automated Python-based retrieval algorithm. A contextual text-mining classifier using a predefined microsurgery keyword taxonomy was applied to identify relevant publications. Candidate forecasting models included linear regression, quadratic regression, autoregressive integrated moving average, and Holt's exponential smoothing. Model performance was compared using R^2, root mean square error, mean absolute error, and Akaike information criterion. Forecasts were generated through 2030 and reported with 95% confidence intervals.

Results: *The framework processed 90,902 records, of which 83,133 underwent contextual text classification after exclusion of incomplete metadata. A final analytic dataset of 11,561 microsurgery publications with verifiable first-author country attribution was identified. Classification validation using a stratified sample of 4,441 records demonstrated 95.2% agreement with the human-reviewed reference standard (95% CI, 94.5%–95.8%; Cohen's $\kappa = 0.826$). Annual microsurgery publications increased from 611 in 2010 to 973 in 2024, representing a 59.3% increase. Temporal validation supported short-horizon stability of the linear projection model. In fixed temporal holdout testing, the linear model achieved RMSE 40.5, MAE 36.2, MAPE 4.0%, and 95% prediction-interval coverage of 100%. Through 2030, publication activity is projected to increase, with lymphatic microsurgery showing the greatest relative thematic growth (+36.0%), followed by technological and operative innovation (+21.7%).*

Conclusions:

This AI-assisted bibliometric and forecasting framework enables scalable, reproducible assessment of microsurgical research activity across time, geography, and thematic do-

mains. Rather than offering deterministic predictions, the model provides clinically interpretable projections of where microsurgical scholarship is expanding. For surgeons, clinician-scientists, and interdisciplinary research teams, this approach may help identify emerging areas of innovation, guide collaboration, and inform strategic research planning.

2. The Introduction section is too brief and requires substantial expansion. The authors should clearly discuss: Existing research gaps, Motivation for the study, Research objectives/questions, Novel contributions of the proposed framework.

Reply: We thank the reviewer for this important comment. We agree that the original Introduction was too brief and did not sufficiently articulate the conceptual rationale, research gap, study objectives, and novel contribution of the proposed framework. We have substantially revised the **Introduction** to clarify why traditional bibliometric approaches remain largely retrospective, why forecasting scientific output is relevant to microsurgery and reconstructive surgery, and how an AI-assisted workflow may help surgeons and clinician-scientists anticipate emerging areas of research activity.

Importantly, we revised this section while preserving the intended scope of the manuscript. This work is directed toward an interdisciplinary medical and surgical readership, including microsurgeons, reconstructive surgeons, clinician-scientists, and academic research teams. Therefore, the Introduction now provides sufficient methodological context without reframing the manuscript as a purely technical computer science paper. The revised text explicitly states the study's motivation, research questions, and contributions: large-scale automated PubMed extraction, contextual classification of microsurgery-related literature, forecasting of global and thematic publication trends, and clinically interpretable projections of research activity through 2030.

3. A dedicated Literature Review / Related Work section is missing. The manuscript should discuss existing bibliometric forecasting studies, AI-based trend prediction approaches, and related microsurgery bibliometric analyses. The limitations of previous studies and the novelty of the proposed work should be explicitly highlighted.

Reply: We thank the reviewer for this helpful suggestion. We agree that the manuscript would

benefit from a more explicit positioning within the existing literature. We have therefore added a concise *Literature Review and Related Work* section after the Introduction. This new section summarizes prior work in surgical bibliometrics, AI-assisted metadata extraction, text mining, and publication forecasting, while emphasizing that this manuscript is intended for surgeons, clinician-scientists, and interdisciplinary medical teams rather than as a purely technical machine-learning paper. We also clarify that this line of work has already been implemented in the aesthetic surgery literature [1], and that large-scale surgical metadata extraction using web scraping was previously introduced and pioneered by this group in the World Journal of Surgery [2]. The revised section explicitly highlights the limitation of prior studies as largely retrospective and identifies the novelty of the current work as the integration of automated extraction, contextual microsurgery classification, and clinically interpretable forecasting in a single reproducible framework.

Added manuscript text:

Literature Review and Related Work

Bibliometric analysis has become an important method for evaluating the evolution of medical and surgical research. In surgery, prior studies have used bibliometric and web-based metadata extraction approaches to quantify publication output, authorship patterns, international collaboration, research productivity, and disparities in academic representation [2]. In particular, large-scale surgical metadata extraction using web scraping was previously introduced by this group as a scalable method for collecting and structuring PubMed-indexed surgical publications, demonstrating that automated extraction can transform months of manual curation into reproducible datasets generated within hours [2]. This methodological foundation has since been applied to evaluate research productivity, human capital, collaboration, and academic output across surgical disciplines [2, 3, 4, 5, 6].

More recently, this line of work has been extended from retrospective surgical bibliometrics toward forecasting models in the aesthetic surgery literature [1]. These studies illustrate the potential value of using large-scale publication metadata not only to describe what has already occurred, but also to estimate how academic fields may evolve under observed historical trends. However, most existing bibliometric studies remain primarily

descriptive, focusing on publication counts, citations, authorship, journals, institutions, or countries. Similarly, prior microsurgery-related bibliometric analyses have largely mapped historical productivity rather than projecting future geographic or thematic trajectories [7]. As a result, the literature provides useful insight into where microsurgical research has been, but less guidance regarding where the field may be moving.

AI-assisted text mining and natural language processing provide a practical approach for organizing large biomedical corpora while preserving domain-specific meaning [8, 9, 10, 11]. In the present study, these methods are used as an analytic aid for surgeons and interdisciplinary research teams, not as an autonomous clinical or technical AI system. The goal is to improve the scale, reproducibility, and thematic resolution of literature-based research assessment while maintaining clinical interpretability.

The novelty of the present work lies in applying this framework specifically to microsurgery, a field that spans reconstructive, lymphatic, peripheral nerve, traumatic, oncologic, and technology-driven research. By integrating automated PubMed metadata extraction, contextual classification of microsurgery-relevant articles, and comparative forecasting models, this study extends prior retrospective bibliometric work into a clinically interpretable projection framework. This approach is intended to help surgeons, clinician-scientists, and interdisciplinary medical teams identify emerging research domains, recognize persistent geographic disparities, and support strategic planning across reconstructive and allied surgical fields.

4. The manuscript repeatedly refers to a "dual-AI framework"; however, the technical description of the AI components is insufficient. The authors should clearly explain: Which AI/NLP models were used, How article classification was performed, Model training and validation procedures, Why the framework qualifies as an AI-based system.

Reply: We thank the reviewer for this important clarification. We agree that the original manuscript did not define the term "dual-AI framework" with sufficient precision and may have unintentionally overstated the technical nature of the approach. To address this concern, we revised the terminology throughout the manuscript and now describe the method as an *AI-assisted dual-component forecasting framework*. We also revised the title from *"Predicting the Future of Microsurgical Science: An AI Framework for Global Publication Forecasting"* to *"AI-Assisted Forecasting in Microsurgery:*

A Dual-Component Framework for Global Publication Trends". This revised title more accurately reflects the actual design of the study: an AI-enabled literature extraction/classification component combined with a statistical forecasting component.

The first component is an AI-enabled automated metadata extraction/web-scraping pipeline, previously introduced by this group in the surgical literature [2]. The second component combines contextual text-mining classification of microsurgery-relevant publications with comparative forecasting models used to project publication trends through 2030. We have clarified that the article classification step was not a supervised deep-learning model trained de novo, but rather a transparent contextual text-mining classifier based on a predefined microsurgery keyword taxonomy applied to titles and abstracts. We also added methodological detail regarding classification, validation, and model comparison. Forecasting models included linear regression, quadratic regression, autoregressive integrated moving average, and Holt's exponential smoothing, with performance evaluated using R^2, root mean square error, mean absolute error, and Akaike information criterion.

Finally, we have moderated the language throughout the manuscript to clarify that the framework is AI-assisted rather than AI-autonomous. Its AI-enabled contribution lies in scalable automated extraction, structuring, and contextual classification of a large biomedical literature corpus. The forecasting component is intentionally transparent and interpretable, consistent with the purpose of this manuscript as an interdisciplinary medical research study for surgeons, clinician-scientists, and research teams rather than a purely technical machine-learning paper.

Revised manuscript text:

AI-Assisted Dual-Component Framework

In this study, the term "dual-component framework" refers to an AI-assisted literature analytics workflow rather than a fully autonomous deep-learning system. The first component consisted of automated PubMed metadata extraction using a Python-based web-scraping algorithm. This approach has previously been introduced by our group in the surgical literature as a scalable method for extracting and structuring large volumes of PubMed-indexed surgical publication data [2]. The algorithm retrieved article-level metadata, including title, abstract, authorship, journal, publication year, and affiliation fields, and converted these records into a structured analytic dataset. This component

allowed high-throughput data collection across 20 microsurgery-relevant journals from 2010 to 2024.

The second component consisted of contextual text-mining classification and forecasting. Article classification was performed using a predefined microsurgery keyword taxonomy applied to titles and abstracts. The taxonomy included core microsurgical terms and stems such as *microsurg**, *free flap**, *perforator**, *replantation**, and *lymphatic**. Records meeting thematic criteria were classified as microsurgery-relevant, whereas articles without contextual relevance to reconstructive microsurgery, lymphatic surgery, peripheral nerve reconstruction, replantation, or related operative innovation were excluded.

No supervised neural network or large language model was trained de novo for article classification. Instead, the classification strategy was intentionally designed to be transparent, reproducible, and interpretable for a surgical and interdisciplinary medical readership. This distinction is important: the purpose of the framework was not to introduce a novel technical AI architecture, but to apply AI-assisted automation and text-mining methods to a clinically meaningful research question in microsurgical science.

Following classification, validated annual publication counts were analyzed using comparative forecasting models, including linear regression, quadratic regression, autoregressive integrated moving average, and Holt's exponential smoothing [12]. Model performance was evaluated using R^2, root mean square error, mean absolute error, and Akaike information criterion [13]. Linear regression was selected as the primary model because it provided the most favorable balance of fit, parsimony, stability, and interpretability for the 15-year annual dataset. Forecasts were generated through 2030 and reported with 95% confidence intervals.

Thus, the framework is best understood as AI-assisted rather than AI-autonomous. Its AI-enabled contribution lies in the scalable extraction, structuring, and contextual classification of a large biomedical literature corpus, followed by transparent statistical forecasting. This design was selected because the intended users are surgeons, clinician-scientists, and interdisciplinary medical research teams who require reproducible and clinically interpretable projections rather than a black-box computational model.

5. In Section 2.2, the authors state that Table S1 contains the list of included journals. However,

Table S1 contains research intensity forecasting results instead of journal information. This inconsistency should be corrected and all supplementary materials should be carefully verified.

Reply: We thank the reviewer for this careful comment. We rechecked the supplementary materials and confirm that Table S1 is intended to contain the list of included journals. Therefore, the statement in Section 2.2 referring readers to Table S1 for the journal list is correct. However, we recognize that the supplementary table numbering, captions, or ordering may have created confusion during review. To address this, we carefully reviewed the supplementary appendix and corrected the table numbering, titles, captions, and cross-references throughout the manuscript and supplementary materials. Table S1 is now clearly labeled as the list of included journals, while the research intensity forecasting results are assigned to the appropriate subsequent supplementary table.

6. The dataset description should be summarized in a dedicated table, including: Total records retrieved, Number of journals included, Records removed due to missing metadata, Records excluded after classification, Final dataset size, Study period.

Reply: We thank the reviewer for this helpful suggestion. These dataset elements were already presented in Figure 1 and its accompanying legend, which summarizes the flow of included articles from initial retrieval to the final analytic dataset. Specifically, Figure 1 reports the total number of retrieved records, exclusions due to missing metadata, records undergoing text-mining classification, records excluded as non-microsurgical, and the final dataset size. However, we agree that a dedicated tabular summary improves readability and allows readers to identify the dataset structure more quickly. Therefore, we have added a new dataset summary table in the Methods/Results section that includes the requested elements: study period, number of journals, total records retrieved, missing metadata exclusions, classification exclusions, exclusions due to unverifiable first-author country, and final analytic sample.

7. Although source code has been made available, the underlying processed dataset is not accessible. The authors should clarify how independent researchers can reproduce the reported findings and results.

Reply: We thank the reviewer for this important comment. We agree that reproducibility should be clarified more explicitly. In the revised manuscript, we expanded the Data and Code Availability statement to explain how independent researchers can reproduce the workflow. The full source code for data extraction, classification, model comparison, and forecasting has been made publicly available. Because the article-level dataset contains author names, institutional affiliations, country assignments, and article-level metadata extracted and manually adjudicated from PubMed records, we did not include the full processed article-level dataset in the public repository.

To improve reproducibility while preserving responsible data sharing, we have clarified that the workflow can be rerun using the public PubMed metadata source, the included journal list, the search strategy, the keyword taxonomy, and the released analysis code. We also added that the aggregated, de-identified annual counts underlying the forecasting analyses can be made available as supplementary material or upon request, including publication counts by year, country, income group, and thematic category. This allows independent researchers to reproduce the reported forecasting models without requiring access to author-level identifiable metadata.

Revised manuscript text:

Data and Code Availability

The forecasting and analysis code used in this study has been deposited in a public GitHub repository and archived on Zenodo to facilitate transparency and reproducibility. The repository contains a standalone Python implementation of the data-processing, model-comparison, and forecasting workflow, including linear regression, quadratic polynomial regression, autoregressive integrated moving average, and Holt's exponential smoothing. The repository also includes synthetic example data to allow independent testing of the analytical pipeline.

The full article-level processed dataset is not included in the public repository because it contains author names, institutional affiliations, manually adjudicated country assignments, and article-level metadata extracted from PubMed records. However, all source data were derived from publicly accessible PubMed metadata, and the manuscript provides the included journal list, study period, classification taxonomy, exclusion criteria, and analytic workflow required to reproduce the dataset construction process. Indepen-

dent researchers can therefore rerun the extraction and classification pipeline using the public code and PubMed as the source database.

To further support reproducibility of the reported findings, aggregated de-identified data underlying the forecasting analyses are available as supplementary material or upon reasonable request. These include annual publication counts by year, country, World Bank income group, and thematic category. These aggregate data allow reproduction of the model-comparison and forecasting results without requiring public release of article-level author or affiliation metadata.

Archived release: https://doi.org/10.5281/zenodo.20396105

Version: v0.1.0

License: MIT

8. The manuscript lacks an architecture/workflow diagram of the proposed dual-AI framework. A detailed block diagram illustrating data collection, preprocessing, AI-based classification, forecasting models, and output generation should be included. Insufficient methodological detail about the AI framework.

Reply: We thank the reviewer for this helpful suggestion. We agree that a schematic workflow diagram improves methodological transparency. Accordingly, we added a new architecture figure (**Figure 1**) in the main manuscript illustrating the full AI-assisted dual-component workflow, including journal selection and PubMed retrieval, metadata extraction, preprocessing and cleaning, contextual text-mining classification, manual validation of ambiguous records, forecasting model comparison, and output generation. We also expanded the Methods section to describe each step more explicitly. This addition was intended to improve clarity for the manuscript's target readership of surgeons, clinician-scientists, and interdisciplinary medical teams, while preserving the paper's clinically oriented rather than purely technical focus.

9. Several references related to AI, text mining, and forecasting methodologies are outdated. More recent studies (2022–2026) should be incorporated.

Reply: We thank the reviewer for this helpful comment. We agree that the manuscript should better reflect the recent expansion of AI-assisted methods in medicine, surgery, text mining, and

forecasting. We have updated the Introduction, Literature Review/Related Work, and Discussion sections to incorporate more recent literature from 2022–2026 wherever appropriate, including contemporary work on AI in surgery, explainable AI in predictive modeling, AI-assisted surgical research infrastructure, web-based metadata extraction, and AI integration in reconstructive and microsurgical practice.

At the same time, we intentionally avoided converting the manuscript into a technical review of AI model architectures, as this study is directed toward surgeons, clinician-scientists, and interdisciplinary medical research teams rather than a purely computational audience. Accordingly, the revised references were selected to support the clinical and methodological rationale of the study: scalable metadata extraction, contextual literature classification, transparent forecasting, and the use of AI-assisted tools to understand evolving surgical research ecosystems.

10. The manuscript should include a dedicated limitations subsection discussing potential biases associated with PubMed-only data collection, article classification, and forecasting assumptions.

Reply: We thank the reviewer for this important comment. The manuscript already included a dedicated *Limitations* section addressing several of these issues, including dependence on PubMed metadata completeness, exclusion of records with incomplete affiliation data, potential undercapture of unconventional microsurgery terminology, journal-selection constraints, PubMed-indexing limitations, and the assumption-dependent nature of forecasting from historical publication trends. However, we agree that these limitations should be stated even more explicitly in relation to the three specific areas raised by the reviewer: PubMed-only data collection, article classification, and forecasting assumptions.

Accordingly, we revised the *Limitations* section to include an additional paragraph directly addressing these potential sources of bias. We also moderated the interpretation of the projections throughout the manuscript to emphasize that they represent trend-based estimates under observed historical assumptions rather than deterministic predictions.

Added manuscript text:

> *An additional consideration is that each stage of the workflow may introduce distinct sources of bias. PubMed-only data collection may underrepresent microsurgery-related*

scholarship published in non-indexed journals, regional journals, conference proceedings, or multidisciplinary outlets not included in the selected journal set. The article classification strategy may also introduce misclassification bias, particularly for studies using nonstandard terminology, reporting microsurgical techniques indirectly, or spanning multiple reconstructive domains. Finally, the forecasting models assume that historical publication trajectories provide a reasonable basis for short-term projection; they cannot incorporate unpredictable changes in funding priorities, institutional capacity, geopolitical conditions, publication practices, or disruptive technological advances. For these reasons, the projected trends should be interpreted as structured estimates of publication activity under observed historical conditions, rather than definitive predictions of future scientific leadership, innovation, or clinical impact.

11. The manuscript would benefit from professional English language editing to improve readability and clarity.

Reply: We thank the reviewer for this comment. The manuscript was carefully revised for readability, clarity, sentence structure, and consistency of terminology. In particular, we edited sections that could be perceived as overly dense or repetitive, standardized the use of key terms such as "AI-assisted," "forecasting," "projection," and "dual-component framework," and moderated language that may have implied deterministic prediction. These revisions were performed to improve manuscript flow for the intended multidisciplinary readership of surgeons, clinician-scientists, and medical research teams.

12. The authors repeatedly claim the use of a "dual-AI framework," but the actual methodology appears to rely primarily on: PubMed data extraction, Text filtering/classification, Linear Regression, Polynomial Regression, ARIMA, Holt Exponential Smoothing.

Reply: We thank the reviewer for this important clarification. We agree that the original phrasing may have overstated the technical nature of the framework. To address this, we revised the terminology throughout the manuscript and replaced references to a "dual-AI framework" with more precise language, including *"AI-enabled dual-component framework"* and *"AI-assisted forecasting framework."*

The revised wording better reflects the actual methodology. The framework consists of an

AI-enabled automated metadata extraction and contextual text-mining component, followed by transparent statistical forecasting using linear regression, quadratic regression, ARIMA, and Holt's exponential smoothing. We now explicitly state that the forecasting component is not itself an advanced AI model or neural-network architecture. Rather, the AI-enabled contribution lies in scalable automated extraction, structuring, and classification of a large biomedical literature corpus, while the forecasting models were intentionally selected for interpretability and suitability for a clinician-scientist readership.

13. These are mostly conventional statistical and bibliometric techniques. The manuscript should clearly justify why this framework is considered AI-driven rather than a traditional bibliometric forecasting pipeline.

Reply: We thank the reviewer for this important point. We agree that several components of the analysis, particularly the forecasting models, are conventional and intentionally interpretable statistical methods. In response, we revised the manuscript to avoid characterizing the study as a fully "AI-driven" or autonomous machine-learning system. Instead, we now describe the approach as an *AI-enabled dual-component framework* or *AI-assisted forecasting framework*. This revised terminology more accurately reflects the methodology and directly addresses the distinction raised by the reviewer.

The framework is considered AI-enabled because the first component uses automated web-based metadata extraction and contextual text-mining to retrieve, structure, and classify a large biomedical literature corpus at a scale that would not be feasible through manual curation. This approach builds on prior surgical literature from our group demonstrating the use of web scraping and automated metadata extraction as scalable tools for surgical research [2]. The second component applies transparent statistical forecasting models, including linear regression, quadratic regression, ARIMA, and Holt's exponential smoothing, to the validated dataset. We now explicitly state that the forecasting models themselves are not advanced AI algorithms. Rather, the AI-enabled contribution lies in scalable data acquisition, structuring, and contextual classification, while the forecasting stage was kept statistically transparent to preserve interpretability for surgeons, clinician-scientists, and interdisciplinary medical teams.

14. The manuscript does not compare its findings against: Previous microsurgery bibliometric anal-

yses, Existing forecasting studies, Similar scientometric research, Without benchmarking against prior work, it is difficult to assess the added value of the proposed framework.

Reply: We thank the reviewer for this important comment. We agree that the manuscript should more clearly benchmark the present study against prior microsurgery bibliometric analyses, surgical scientometric studies, and existing forecasting work. This issue is now addressed in the newly added Literature Review and Related Work section, which discusses prior surgical metadata-extraction studies, the previous implementation of this line of work in aesthetic surgery forecasting, AI-assisted text mining, and prior microsurgery-related bibliometric analyses.

In the revised manuscript, we clarify that previous microsurgery-related bibliometric studies have largely been retrospective, focusing on historical publication productivity, authorship, journals, institutions, countries, and thematic patterns. To our knowledge, no prior microsurgery-specific study has applied a structured forecasting framework to project future geographic and thematic research trajectories. To further emphasize the added value of the present work, we also added a concise paragraph to the Discussion clarifying that this study extends prior retrospective mapping into a clinically interpretable projection framework for surgeons, clinician-scientists, and interdisciplinary medical teams.

Added Discussion text:

> *The added value of this study should be interpreted in relation to prior surgical scientometric work. Previous microsurgery-related bibliometric analyses have provided important retrospective descriptions of publication productivity, influential articles, authorship patterns, countries, journals, and research themes [7]. However, these studies have primarily mapped where the field has been rather than modeling where it may be moving. In contrast, the present framework integrates automated metadata extraction, contextual microsurgery classification, and comparative forecasting to generate clinically interpretable projections of publication activity across geography and research domains. This distinction is important for surgeons and interdisciplinary research teams because trend-based forecasting may help identify emerging areas of scholarly activity, persistent geographic disparities, and future opportunities for collaboration and academic planning.*

15. The classification framework reports high reproducibility but does not analyze: Misclassified ar-

ticles, False positives, False negatives, Challenging cases. An error analysis section would strengthen confidence in the methodology.

Reply: We thank the reviewer for this valuable suggestion. We agree that reporting overall agreement alone does not fully characterize the performance of the classification framework. To address this, we added a dedicated error analysis subsection describing the main sources of false-positive and false-negative classifications observed during manual validation. We also clarified that the reported value reflects agreement between the automated classifier and investigator consensus, rather than reproducibility in the strict statistical sense.

The revised manuscript now describes the most common challenging cases, including articles using microsurgical terminology in a non-reconstructive context, articles involving free-tissue transfer without explicit microsurgical language, studies spanning multiple reconstructive domains, and articles with incomplete or nonspecific abstracts. Where available, we also added classification performance metrics, including false-positive and false-negative rates, to improve transparency and confidence in the article selection process.

Added manuscript text:

Classification Error Analysis

To further evaluate the performance of the contextual classification framework, misclassified and borderline records identified during manual validation were reviewed qualitatively. The most common false-positive cases involved articles that contained microsurgery-related terms but were not primarily focused on reconstructive microsurgery. Examples included studies using terms such as "microvascular" in vascular, or basic science contexts, as well as articles mentioning flap terminology without reporting a microsurgical reconstructive procedure. These cases illustrate the challenge of distinguishing true microsurgical content from adjacent surgical or biomedical literature using terminology alone.

False-negative cases were less frequent but generally occurred when articles described microsurgical concepts without using the predefined keyword taxonomy in the title or abstract. Examples included studies of free-tissue transfer, lymphatic reconstruction,

perforator-based reconstruction, or peripheral nerve repair that used indirect or non-standard terminology. Additional challenging cases included articles spanning multiple reconstructive domains, abstracts with limited procedural detail, and studies in which the microsurgical component was present but not central to the article's stated objective.

These errors were mitigated through iterative refinement of the keyword taxonomy and manual adjudication of ambiguous records by two investigators. The final validation sample demonstrated high agreement between the automated classification output and investigator consensus. Nevertheless, we recognize that a taxonomy-based contextual classifier may undercapture emerging terminology or overcapture adjacent literature when microsurgery-related terms are used nonspecifically. For this reason, the classification framework should be interpreted as a transparent and reproducible screening method rather than a fully autonomous semantic model. Future versions of the framework may incorporate supervised machine-learning or embedding-based semantic classification to improve recall while preserving specificity.

16. The manuscript frequently uses causal language when only descriptive bibliometric analyses are presented. Statements implying causes of productivity growth should be moderated.

Reply: We thank the reviewer for this important methodological comment. We agree that causal language should be avoided because the present study is based on descriptive bibliometric analyses and trend-based forecasting, not causal inference. We therefore revised the manuscript throughout to moderate language that could imply causality.

In addition, we added a dedicated paragraph to the *Limitations* section explicitly stating that this study reports descriptive associations and projections rather than causal findings. We also cited our prior methodological work on causal inference in medical research, which emphasizes that associations in observational medical studies should not be interpreted as evidence of causation without an appropriate causal framework [14]. These revisions ensure that the interpretation of the findings remains appropriately aligned with the descriptive and forecasting design of the study.

Added Limitations text:

This study should also be interpreted as a descriptive and forecasting analysis rather than a causal investigation. The observed relationships between publication output, geography,

income classification, authorship patterns, and thematic growth represent associations within the indexed literature and should not be interpreted as causal effects. For example, increases in microsurgery publications from specific countries or thematic domains may coincide with changes in research infrastructure, funding priorities, training capacity, technological adoption, or publication practices, but the present design cannot determine whether any of these factors caused the observed trends. Distinguishing association from causation requires explicit causal assumptions and dedicated causal inference methods, which were outside the scope of this study [14]. Accordingly, all projections should be interpreted as structured estimates based on historical publication patterns rather than causal claims about future scientific productivity, innovation, or leadership.

17. Figures and tables are placed collectively at the end of the manuscript, which negatively affects readability and makes it difficult for readers to follow the results and discussion sections. The authors should place all figures and tables within the main text, immediately after or close to their first citation, in accordance with standard scientific writing practices and to improve manuscript flow and comprehension.

Reply: We thank the reviewer for this comment and agree that readability is important. However, the placement of figures and tables was guided by the PLOS ONE submission requirements and the Editorial Manager submission workflow. PLOS ONE requires figures to be prepared and submitted as individual files rather than embedded directly within the main manuscript file, and Editorial Manager compiles the uploaded manuscript and figure files into a single PDF for review. Therefore, the figures were not embedded in-line within the manuscript body in order to remain consistent with journal submission requirements.

To improve readability while remaining compliant with PLOS ONE formatting, we revised the manuscript to ensure that all figures and tables are cited in ascending order at their first relevant appearance in the text. We also placed figure captions and table callouts close to the first in-text citation where appropriate, and we embedded manuscript tables near their first citation when allowed by the journal format. These changes should improve manuscript flow for reviewers and readers while maintaining compliance with the journal's submission system.

18. The manuscript appears to present bibliometric analysis, text filtering, and statistical forecasting under the umbrella of "Artificial Intelligence." However, the actual methodological novelty is

limited, and the AI component is not sufficiently demonstrated or validated.

Reply: We thank the reviewer for this important comment. We agree that the original wording may have overstated the extent to which the framework should be interpreted as a purely artificial intelligence or machine-learning model. To address this concern, we revised the terminology throughout the manuscript and now describe the approach as an *AI-assisted* or *AI-enabled dual-component forecasting framework*, rather than as a fully AI-driven or autonomous system.

We now explicitly clarify that the novelty of the study does not lie in the development of a new neural-network architecture or a black-box machine-learning predictor. Rather, the contribution lies in integrating AI-enabled automated metadata extraction, contextual text-mining classification, and transparent statistical forecasting into a reproducible workflow for microsurgery research assessment. The forecasting models themselves are conventional and interpretable by design, including linear regression, quadratic regression, ARIMA, and Holt's exponential smoothing. This was intentional, because the manuscript is directed toward surgeons, clinician-scientists, and interdisciplinary medical teams rather than a purely technical AI audience.

We also expanded the Methods section to better describe the two components of the framework and added an architecture/workflow figure illustrating the pipeline from PubMed retrieval to output generation. In addition, we strengthened the validation description by clarifying the manual review process, agreement with investigator consensus, and classification error analysis. These changes more accurately frame the manuscript as an AI-enabled medical research workflow rather than a technical AI model-development paper.

19. Several figure captions are excessively long and contain interpretation rather than description. Interpretive discussion should be moved to the Results or Discussion sections.

Reply: We thank the reviewer for this comment. We agree that figure captions should primarily describe the content of each figure rather than duplicate the interpretation presented in the main text. The Results and Discussion sections already contain the substantive interpretation of the findings. Our original intent was to make each figure sufficiently self-contained so that readers could understand the principal message quickly without repeatedly returning to the manuscript text.

To address the reviewer's concern, we revised the figure captions throughout the manuscript to

retain only the information necessary to explain the displayed variables, analytic approach, observed versus projected periods, uncertainty intervals, and relevant abbreviations. Interpretive statements regarding the clinical, geographic, or scientific meaning of the findings were removed from the captions and retained in the Results and Discussion sections. This preserves figure readability and accessibility while reducing redundancy.

20. The rationale for selecting only 20 journals is insufficient. The authors should explain how these journals were identified and justify whether they adequately represent the global microsurgery literature.

Reply: We thank the reviewer for this important comment. We agree that the journal-selection process required greater transparency. In the revised Methods section, we clarified that the 20 journals were selected *a priori* to provide a broad and clinically representative sampling frame of the specialties in which microsurgical techniques are most commonly practiced and reported. These included plastic and reconstructive surgery, hand surgery, orthopedic and trauma surgery, craniofacial surgery, otolaryngology, and vascular surgery.

Journal selection was based on PubMed indexing, continuity of publication during the 2010–2024 study period, regular publication of clinical or experimental microsurgery-related research, availability of standardized metadata, and representation of both core microsurgery journals and adjacent surgical disciplines. The complete journal list is provided in Supplementary Table S1.

We also clarified that the selected journals were intended to provide a reproducible and clinically relevant representation of the microsurgical literature rather than an exhaustive census of every microsurgery-related publication. Microsurgical studies published in general medical, oncologic, transplantation, regional, or non–PubMed-indexed journals may therefore not have been captured. This limitation is now stated explicitly in the manuscript, and the findings are interpreted as trends within the predefined journal sampling frame.

Reviewer 2 Comments:

This manuscript presents a bibliometric analysis of microsurgery publications using automated article retrieval, thematic classification, and forecasting methods. The dataset is substantial, and the topic is relevant to surgical research and research evaluation. The study also addresses an area that has received comparatively limited attention within the microsurgical literature. However, several aspects of the methodology are insufficiently described, making it difficult to fully assess the validity, reproducibility, and interpretation of the principal findings. In particular, the classification framework, forecasting methodology, validation procedures, and data availability require substantial clarification before the manuscript can be considered for publication.

Reply: We thank the reviewer for the thoughtful and constructive assessment of our manuscript. We appreciate the recognition of the study's substantial dataset, clinical relevance, and contribution to an area that remains comparatively underexplored within the microsurgical literature. We agree that the original manuscript required greater methodological transparency.

In response, we have comprehensively revised the manuscript to address each concern raised. Specifically, we expanded the description of the AI-enabled article retrieval and thematic classification workflow, clarified the construction and validation of the classification framework, strengthened the description and interpretation of the forecasting methodology, added further details regarding model evaluation and uncertainty, expanded the error analysis and validation procedures, and revised the Data and Code Availability statement to improve reproducibility. We also moderated the manuscript's terminology and conclusions to distinguish AI-assisted data processing from conventional statistical forecasting and to emphasize that the reported findings represent descriptive associations and trend-based projections rather than causal or deterministic predictions.

Each of the reviewer's specific comments is addressed individually below, with corresponding revisions incorporated throughout the manuscript and supplementary materials.

1. Clarification of the "Dual-AI" Framework The manuscript repeatedly describes the methodology as a "dual-AI framework." However, based on the methods currently presented, the classification procedure appears to be primarily a rule-based keyword filtering approach using terms such as microsurg, free flap, perforator, replantation, and lymphatic. Automated data retrieval and keyword classification do not, by themselves, constitute artificial intelligence or machine learning. If machine

learning, natural language processing, large language models, or other AI methods were used, these methods should be described in sufficient detail, including model architecture, training procedures, validation methods, and implementation details. If no such methods were employed, the authors should reconsider the characterization of the framework as AI-based and instead describe it as an automated bibliometric pipeline with keyword-based classification.

Reply: We thank the reviewer for this important clarification and agree that the original term "dual-AI framework" was insufficiently precise. No supervised machine-learning classifier, large language model, or neural-network architecture was trained de novo in this study. The article-selection procedure used a transparent rule-based text-mining taxonomy applied to publication titles and abstracts, followed by investigator validation and adjudication of ambiguous records.

Accordingly, we revised the terminology throughout the manuscript and no longer characterize the method as a fully AI-driven system. The approach is now described as an *AI-enabled dual-component framework* or *AI-assisted forecasting framework*. The first component consists of automated PubMed metadata extraction and structuring through a Python-based web-scraping workflow previously introduced in the surgical literature [2]. The second component consists of keyword-based contextual classification followed by conventional statistical forecasting using linear regression, quadratic regression, ARIMA, and Holt's exponential smoothing.

We also revised the title from *"Predicting the Future of Microsurgical Science: An AI Framework for Global Publication Forecasting"* to *"AI-Assisted Forecasting in Microsurgery: A Dual-Component Framework for Global Publication Trends."* These changes more accurately distinguish AI-enabled data processing from the transparent statistical forecasting stage and avoid implying the use of an advanced or autonomous machine-learning architecture. The Methods section has also been expanded to describe the classification taxonomy, implementation, validation, and model-comparison procedures in greater detail.

Revised manuscript text:

AI-Assisted Dual-Component Framework

In this study, the term "dual-component framework" refers to an AI-assisted literature analytics workflow rather than a fully autonomous deep-learning system. The first

component consisted of automated PubMed metadata extraction using a Python-based web-scraping algorithm. This approach has previously been introduced by our group in the surgical literature as a scalable method for extracting and structuring large volumes of PubMed-indexed surgical publication data [2]. The algorithm retrieved article-level metadata, including title, abstract, authorship, journal, publication year, and affiliation fields, and converted these records into a structured analytic dataset. This component allowed high-throughput data collection across 20 microsurgery-relevant journals from 2010 to 2024.

The second component consisted of contextual text-mining classification and forecasting. Article classification was performed using a predefined microsurgery keyword taxonomy applied to titles and abstracts. The taxonomy included core microsurgical terms and stems such as microsurg, free flap*, perforator*, replantation*, and lymphatic*. Records meeting thematic criteria were classified as microsurgery-relevant, whereas articles without contextual relevance to reconstructive microsurgery, lymphatic surgery, peripheral nerve reconstruction, replantation, or related operative innovation were excluded.*

No supervised neural network or large language model was trained de novo for article classification. Instead, the classification strategy was intentionally designed to be transparent, reproducible, and interpretable for a surgical and interdisciplinary medical readership. This distinction is important: the purpose of the framework was not to introduce a novel technical AI architecture, but to apply AI-assisted automation and text-mining methods to a clinically meaningful research question in microsurgical science.

Following classification, validated annual publication counts were analyzed using comparative forecasting models, including linear regression, quadratic regression, autoregressive integrated moving average, and Holt's exponential smoothing [12]. Model performance was evaluated using R^2*, root mean square error, mean absolute error, and Akaike information criterion [13]. Linear regression was selected as the primary model because it provided the most favorable balance of fit, parsimony, stability, and interpretability for the 15-year annual dataset. Forecasts were generated through 2030 and reported with 95% confidence intervals.*

Thus, the framework is best understood as AI-assisted rather than AI-autonomous.

Its AI-enabled contribution lies in the scalable extraction, structuring, and contextual classification of a large biomedical literature corpus, followed by transparent statistical forecasting. This design was selected because the intended users are surgeons, clinician-scientists, and interdisciplinary medical research teams who require reproducible and clinically interpretable projections rather than a black-box computational model.

2. Forecasting Methodology and Predictive Validity The forecasting analyses are based on annual publication counts from 2010 through 2024, yielding only fifteen observations for model fitting. This limited time series constrains the reliability of model selection, uncertainty estimation, and long-range extrapolation through 2030. The manuscript compares multiple forecasting approaches using R^2, RMSE, MAE, and AIC and selects linear regression as the preferred model. However, all reported performance metrics appear to be based on the same data used for model fitting. No temporal holdout validation, rolling-origin validation, or other assessment of out-of-sample performance is presented. Consequently, the analysis demonstrates model fit but does not demonstrate forecasting accuracy. The authors should consider providing a temporal validation strategy (for example, fitting models on earlier years and evaluating performance on later years) or explicitly acknowledge that the reported results represent projections under observed historical trends rather than validated forecasts. In addition, the rationale for selecting linear regression over alternative models should be expanded. Although the linear model achieved the lowest AIC, the differences between models appear modest. Residual diagnostics, assessment of autocorrelation, and discussion of model assumptions would strengthen confidence in the chosen approach.

Reply: We thank the reviewer for this important methodological comment. We agree that the original model evaluation relied primarily on in-sample fit metrics and therefore did not sufficiently demonstrate predictive stability on unseen observations. We also agree that the 15-year annual time series limits the certainty of long-range extrapolation. Accordingly, we revised the Methods, Results, and Limitations sections to clarify that the 2025–2030 estimates should be interpreted as short-horizon, trend-based projections under continuation of observed 2010–2024 publication patterns, rather than deterministic predictions of future scientific activity.

To address the concern regarding predictive validity, we added two temporal out-of-sample validation analyses. First, we performed rolling-origin expanding-window validation, in which models were fit on earlier years and evaluated against held-out observations at one-, two-, and three-year forecast horizons. Second, we performed a fixed temporal holdout analysis, training models on 2010–

2019 data and evaluating predictions against observed publication counts from 2020–2024. Out-of-sample performance was summarized using RMSE, MAE, MAPE, and 95% prediction-interval coverage.

These analyses supported short-horizon stability of the linear projection model. In rolling-origin validation, one-year-ahead linear forecasts achieved RMSE 65.1, MAE 52.8, and MAPE 6.6% across seven validation origins. When pooled across one- to three-year horizons, RMSE was 54.2, MAE was 44.4, and MAPE was 5.3%. In the fixed temporal holdout analysis, the linear model achieved RMSE 40.5, MAE 36.2, MAPE 4.0%, and 95% prediction-interval coverage of 100%.

We also expanded the rationale for selecting linear regression. The revised manuscript no longer states that linear regression was selected primarily because of the lowest AIC/AICc. In the manuscript-analysis series, information criteria did not decisively separate all models; ARIMA had the lowest uniform AICc, while linear regression was within 1.33 AICc units. However, ARIMA performed substantially worse in the 2020–2024 temporal holdout than linear regression, with RMSE 111.5, MAE 103.6, and MAPE 11.4%, compared with RMSE 40.5, MAE 36.2, and MAPE 4.0% for linear regression. Quadratic regression also performed worse in holdout testing, with RMSE 170.6 and MAPE 17.5%. Holt's exponential smoothing performed similarly to linear regression, but linear regression was retained as the primary model because it provided comparable temporal validation performance while offering a directly interpretable annual growth estimate and greater parsimony.

Finally, we added residual diagnostics for the selected linear model. Full-sample diagnostics demonstrated $R^2 = 0.870$, Durbin–Watson statistic 1.78, Ljung–Box $p = 0.218$, Shapiro–Wilk $p = 0.316$, and Breusch–Pagan $p = 0.425$. These diagnostics did not identify statistically significant residual autocorrelation, non-normality, or heteroscedasticity, although we acknowledge that the small number of annual observations limits diagnostic power. These additions strengthen the transparency of the forecasting approach while preserving the appropriate interpretation of the estimates as conditional projections rather than validated predictions of future microsurgical research activity.

Added Methods text:

> *Because only 15 annual observations were available, forecasting results were interpreted as short-horizon projections under observed historical trends rather than deterministic predictions. Model validation therefore focused on temporal back-testing within the observed period and transparent reporting of uncertainty and model assumptions. Tempo-*

ral out-of-sample validation was performed using two complementary approaches. First, rolling-origin expanding-window validation was used, in which models were fit on earlier years and then used to forecast one-, two-, and three-year horizons against held-out observations. Second, a fixed temporal holdout analysis trained models on 2010–2019 and evaluated predictions against observed counts from 2020–2024. Out-of-sample performance was summarized using RMSE, MAE, MAPE, and 95% prediction-interval coverage. Residual diagnostics were performed for the selected linear model. Autocorrelation was assessed using Durbin–Watson and Ljung–Box testing [15], residual normality using the Shapiro–Wilk test [16], and heteroscedasticity using the Breusch–Pagan test [17].

Added Results text:

Temporal validation supported short-horizon stability of the linear projection model. In rolling-origin validation, one-year-ahead linear forecasts achieved RMSE 65.1, MAE 52.8, and MAPE 6.6% across seven validation origins; pooled across one- to three-year horizons, RMSE was 54.2, MAE 44.4, and MAPE 5.3%. In the fixed temporal holdout analysis, with models trained on 2010–2019 and evaluated on 2020–2024, the linear model achieved RMSE 40.5, MAE 36.2, MAPE 4.0%, and 95% prediction-interval coverage of 100%.

Linear regression was selected as the primary projection model based on temporal validation performance, parsimony, stability, and interpretability rather than in-sample fit alone. Although information criteria did not decisively separate all models, the linear model performed well in rolling-origin and temporal holdout validation, substantially outperformed ARIMA and quadratic regression in the 2020–2024 holdout, and provided a directly interpretable annual growth estimate.

Residual diagnostics for the linear model were acceptable for a short annual series: $R^2 = 0.870$, *Durbin–Watson 1.78, Ljung–Box* $p = 0.218$, *Shapiro–Wilk* $p = 0.316$, *and Breusch–Pagan* $p = 0.425$. *No diagnostic test indicated statistically significant residual autocorrelation, non-normality, or heteroscedasticity.*

Added Limitations text:

Fourth, the forecasting models were derived from historical publication trajectories and therefore provide conditional projections rather than validated predictions of future scientific activity. The annual time series includes only 15 observations, which limits the precision of model selection and uncertainty estimates. Although multiple forecasting approaches were compared, 95% confidence intervals were reported, and temporal holdout and rolling-origin validation were added to assess short-horizon predictive stability, these analyses cannot eliminate the uncertainty inherent in extrapolating publication trends through 2030. The models assume that the underlying temporal structure observed from 2010 to 2024 will remain sufficiently stable over the projection period. They cannot account for future disruptions or structural changes, including shifts in funding priorities, research policy, geopolitical conditions, journal indexing, publication practices, workforce composition, or major technological breakthroughs. Moreover, publication volume should be interpreted as a measure of scholarly activity rather than a direct indicator of scientific quality, innovation, clinical significance, or future leadership. Accordingly, projections through 2030 represent scenario-based estimates under continuation of observed 2010–2024 publication trends and should not be interpreted as deterministic forecasts of the future direction or impact of microsurgical science.

3. Classification Validation Requires Additional Detail The manuscript reports greater than 98% reproducibility based on manual review of approximately 5% of the dataset. However, the validation process is not described in sufficient detail. The authors should report:(1)The exact validation sample size, (2) The stratification procedure used for sampling, (3) A confusion matrix, (4) Sensitivity and specificity,(5) Positive and negative predictive values, (6) False-positive and false-negative rates, (7) Inter-rater agreement statistics (e.g., Cohen's kappa). In addition, the manuscript appears to use the terms "agreement" and "reproducibility" interchangeably. These concepts are not identical and should be clearly distinguished. If the reported value refers to agreement with manual review, this should be stated explicitly.

Reply: We thank the reviewer for this important methodological comment. We agree that the original validation description was insufficient and that the terms "agreement" and "reproducibility" were used imprecisely. We have revised the manuscript accordingly. The prior statement reporting >98% "reproducibility" has been removed and replaced with a more detailed validation analysis reporting agreement with a human-reviewed reference standard.

In the revised manuscript, classification performance was evaluated using a stratified validation sample of 4,441 records, representing 5.3% of the 83,133 records that underwent relevance classification. Sampling was stratified by classifier label, publication year, and journal to ensure representation of both predicted relevant and predicted non-relevant records across the full study period. The final human-reviewed adjudicated label served as the reference standard.

We now report the full confusion matrix and classifier performance metrics. Against the human-reviewed reference standard, the classifier achieved 95.2% agreement (95% CI, 94.5%–95.8%) and Cohen's κ of 0.826. The confusion matrix included 627 true positives, 112 false positives, 101 false negatives, and 3,601 true negatives. Sensitivity was 86.1%, specificity 97.0%, positive predictive value 84.8%, negative predictive value 97.3%, false-positive rate 3.0%, false-negative rate 13.9%, and F1 score 0.855.

We also clarified the terminology throughout the manuscript. The validation result is now described as agreement with a human-reviewed reference standard, not reproducibility. We reserve the term reproducibility for deterministic re-execution of the automated pipeline using fixed code, rules, and parameters.

Added Methods text:

> *Classification validation used a stratified sample drawn from all records that underwent relevance classification. Sampling was stratified by classifier label, publication year, and journal to represent both predicted relevant and predicted non-relevant records across the full study period. The final human-reviewed adjudicated label served as the reference standard for performance metrics. Classifier performance was summarized using overall agreement, Cohen's κ, sensitivity, specificity, positive predictive value, negative predictive value, false-positive rate, false-negative rate, and F1 score.*

Added Results text:

> *Classification accuracy was evaluated using a stratified validation sample of 4,441 records, representing 5.3% of the 83,133 classified source records. The sample was stratified by classifier label, publication year, and journal. Against the human-reviewed reference standard, the classifier achieved 95.2% agreement (95% CI, 94.5%–95.8%) and Cohen's κ of 0.826. The confusion matrix included 627 true positives, 112 false positives,*

101 false negatives, and 3,601 true negatives. Sensitivity was 86.1%, specificity 97.0%, positive predictive value 84.8%, negative predictive value 97.3%, false-positive rate 3.0%, false-negative rate 13.9%, and F1 score 0.855.

The 213 classifier-reference discordant records included 112 false positives and 101 false negatives. False-positive classifications most commonly reflected adjacent non-microsurgical uses of terms such as "anastomosis" or "microvascular." False-negative classifications generally involved records using indirect or nonstandard terminology for free-flap reconstruction, perforator-flap surgery, lymphatic microsurgery, or replantation. These findings were used to characterize the limitations of the taxonomy-based classifier and to guide interpretation of the final dataset.

4. Human Capital Proxy Requires Further Justification The study uses first author publication counts as a proxy for national research capacity and human capital. Although references are provided to support this approach, first authorship remains an indirect surrogate measure with recognized limitations. Authorship conventions vary across disciplines, countries, and collaborative structures. Multiple first authorship is increasingly common, and first author attribution may not fully capture mentorship, leadership, or collaborative contributions. The limitations associated with this proxy should be discussed more thoroughly. A sensitivity analysis using an alternative authorship metric would further strengthen confidence that the reported findings are not dependent on the specific choice of proxy.

Reply: We thank the reviewer for this thoughtful comment. We agree that first authorship is an indirect proxy for research-producing human capital and does not fully capture the broader collaborative, mentorship, and leadership structures underlying scientific activity. We have therefore expanded the Limitations section to address variation in authorship conventions, co-first authorship, multidisciplinary team structures, and the possibility that first-author counts may incompletely represent national research capacity.

At the same time, the use of first authorship is supported by a substantial body of bibliometric and authorship literature. In biomedical research, first authors typically make the largest contributions to study conception, execution, data analysis, and manuscript preparation and are commonly regarded as the principal drivers of individual scientific projects [18, 19, 20]. First-author counts have also been used in prior global surgical research to operationalize research-producing human

capital and compare national academic capacity [21, 22, 5, 4]. We have strengthened this justification in the revised Methods section.

We carefully considered the requested sensitivity analysis. However, there is no directly interchangeable authorship metric that measures the same construct with comparable validity. Senior authorship more strongly reflects supervision, mentorship, or laboratory leadership; total authorship counts conflate substantive project leadership with participation; corresponding authorship is inconsistently indexed and varies across journals; and co-first authorship is not uniformly captured in PubMed metadata. Substituting one of these measures would therefore not represent a conventional sensitivity analysis of the same exposure, but rather an analysis of a different and ambiguously defined construct. Such an analysis could dilute the intended signal and complicate interpretation rather than test the robustness of the first-authorship proxy.

Accordingly, we retained first authorship as the prespecified and literature-supported measure of research-producing human capital, while explicitly acknowledging that it captures only one dimension of national academic capacity. We have also identified alternative authorship-network analyses as an important direction for future work.

Revised Methods text:

Human Capital in Surgery: Operational Definition

Human capital, broadly defined as the set of individual competencies contributing to knowledge production [23], was operationalized in this study using first authorship of scientific publications. This approach reflects established evidence that first authors typically represent the primary intellectual drivers of a project and are most likely to continue producing future research, thereby serving as a measurable proxy for a country's capacity to generate new surgical knowledge [23, 24, 21, 20, 25].

The rationale for this proxy aligns with longstanding conventions in biomedical publishing, where first authorship is attributed to the contributor with the most substantial scientific input [18]. Empirical analyses further demonstrate that first authors—compared with middle authors—exhibit broader and more diverse contributions across study design, analysis, interpretation, and manuscript preparation, whereas intermediate authors more commonly participate in task-specific roles such as data collection [19].

This interpretation of first authorship as a proxy for national research-producing personnel is widely adopted in global surgical scholarship and has been used extensively to quantify research capacity across countries and subspecialties [21, 22, 26, 27]. In this forecasting framework, first-author counts therefore serve as a standardized and reproducible indicator of human capital in microsurgery.

Importantly, this operational definition was intended to capture active research production rather than the full spectrum of academic leadership, mentorship, or collaboration. Senior authorship, corresponding authorship, total coauthorship, and coauthorship-network position reflect related but conceptually distinct dimensions of academic activity and are not directly interchangeable with first authorship. Senior authorship may more strongly represent supervision or laboratory leadership, while total authorship counts may combine substantive scientific leadership with more limited task-specific participation. Corresponding authorship is also inconsistently indexed across journals and databases, and shared first authorship is not uniformly represented in PubMed metadata.

For these reasons, alternative authorship measures were not treated as conventional sensitivity analyses of the same construct. Substituting senior, corresponding, or total authorship would evaluate different dimensions of academic contribution and could dilute the specific signal of research-producing personnel rather than test the robustness of the first-authorship proxy. Accordingly, first authorship was retained as the prespecified, literature-supported, and reproducible measure of national research-producing human capital, while its limitations are acknowledged explicitly.

5. Data Availability and Reproducibility The public availability of the code repository is commendable and supports reproducibility. However, the analytical dataset used to generate the reported results does not appear to be publicly available. Because the study is based on publicly available PubMed metadata rather than protected human subject data, the rationale for withholding the analytical dataset is unclear. PLOS ONE places strong emphasis on data availability and reproducibility. At a minimum, the authors should consider providing the aggregated annual publication counts by country and thematic category that underlie the forecasting analyses. If sharing the full article level dataset is not possible, the reasons should be clearly explained.

Reply: We thank the reviewer for this important comment. We agree that the original Data and

Code Availability statement required greater clarity regarding the materials available for independent reproduction of the study.

The complete forecasting and analysis code remains publicly available through GitHub and Zenodo. The repository includes a standalone Python implementation of the data-processing, model-comparison, and forecasting workflow, together with synthetic example data that permit independent testing of the analytical pipeline. Because the source records were obtained from publicly accessible PubMed metadata, researchers may reconstruct the study dataset using the specified journal list, study period, classification taxonomy, exclusion criteria, and publicly available code.

The full processed article-level dataset is not deposited in the public repository because it contains author names, institutional affiliations, and manually adjudicated country assignments assembled during the study. However, the processed data underlying the reported analyses, including aggregated annual publication counts by year, country, World Bank income group, and thematic category, are available from the corresponding author upon reasonable request. We have revised the Data and Code Availability statement accordingly.

Revised Data and Code Availability statement:

> *The forecasting and analysis code used in this study has been deposited in a public GitHub repository and archived on Zenodo to facilitate transparency and reproducibility. The repository contains a standalone Python implementation of the data-processing, model-comparison, and forecasting workflow, including linear regression, quadratic polynomial regression, autoregressive integrated moving average, and Holt's exponential smoothing. The repository also includes synthetic example data to allow independent testing of the analytical pipeline.*
>
> *The full article-level processed dataset is not included in the public repository because it contains author names, institutional affiliations, manually adjudicated country assignments, and article-level metadata extracted from PubMed records. However, all source data were derived from publicly accessible PubMed metadata, and the manuscript provides the included journal list, study period, classification taxonomy, exclusion criteria, and analytic workflow required to reproduce the dataset construction process. Independent researchers can therefore rerun the extraction and classification pipeline using the public code and PubMed as the source database.*

To further support reproducibility of the reported findings, aggregated de-identified data underlying the forecasting analyses are available as supplementary material or upon reasonable request. These include annual publication counts by year, country, World Bank income group, and thematic category. These aggregate data allow reproduction of the model-comparison and forecasting results without requiring public release of article-level author or affiliation metadata.

6. Interpretation of Findings and Strength of Conclusions Several statements throughout the manuscript appear stronger than the available evidence supports. Terms such as "predicting the future of microsurgical science," "anticipating innovation," and similar language imply a degree of predictive certainty that exceeds what can be established from publication count projections. Publication volume is a useful indicator of research activity but should not be interpreted as a direct measure of innovation, scientific impact, clinical significance, or future leadership. The discussion and conclusion should be revised to distinguish projections from predictions and to acknowledge more explicitly the limitations inherent in extrapolating historical publication trends.

Reply: We thank the reviewer for this important interpretive comment. We agree that several statements in the original manuscript were stronger than warranted by the underlying data and could be read as implying a level of predictive certainty not supported by trend-based publication modeling.

Accordingly, we revised the manuscript throughout to distinguish more clearly between *projection* and *prediction*. Language such as "predicting the future," "anticipating innovation," "future leadership," and similar deterministic phrasing was softened or replaced with more precise terms, including "projecting publication trends," "estimating future research activity," "identifying areas of increasing scholarly output," and "conditional projections under observed historical patterns." We also clarified that publication volume is an indicator of research activity and productivity, but not a direct measure of scientific quality, innovation, clinical impact, or future leadership.

In addition, we expanded the relevant paragraph in the *Limitations* section to state more explicitly that the forecasts are conditional extrapolations derived from historical publication counts and cannot account for future structural changes, funding priorities, indexing practices, disruptive technologies, geopolitical events, or shifts in publication behavior.

Revised Limitations text:

Fourth, the forecasting models were derived from historical publication trajectories and therefore provide conditional projections rather than validated predictions of future scientific activity. Although multiple forecasting approaches were compared and 95% confidence intervals were reported, the models assume that the underlying temporal structure observed from 2010 to 2024 will remain sufficiently stable over the projection period. They cannot account for future disruptions or structural changes, including shifts in funding priorities, research policy, geopolitical conditions, journal indexing, publication practices, workforce composition, or major technological breakthroughs. Moreover, publication volume should be interpreted as a measure of scholarly activity rather than a direct indicator of scientific quality, innovation, clinical significance, or future leadership. Accordingly, projections through 2030 represent scenario-based estimates under continuation of observed historical trends and should not be interpreted as deterministic forecasts of the future direction or impact of microsurgical science.

7. Journal Selection Process The manuscript states that twenty journals were included but provides limited information regarding how these journals were selected. Greater transparency regarding journal identification, inclusion criteria, and potential exclusions would strengthen the methodological rigor of the study.

Reply: We thank the reviewer for this important comment. We agree that the journal-selection process required greater transparency. As also requested by the previous reviewer, we expanded the Methods section to clarify how the 20 journals were identified and selected, including the predefined eligibility criteria, the range of microsurgery-practicing specialties represented, and the rationale for including both core and adjacent surgical journals. The complete journal list is provided in Supplementary Table S1.

In addition, we added a dedicated paragraph to the *Limitations* section explicitly acknowledging the potential selection bias introduced by the predefined journal set and PubMed-only indexing. This paragraph clarifies that the study was designed to provide a broad, reproducible, and clinically meaningful sampling frame rather than an exhaustive census of all global microsurgery publications.

Added Limitations text:

Third, journal selection and PubMed indexing impose an important scope constraint.

Although the 20 journals were selected a priori to represent the principal specialties in which microsurgical techniques are practiced and reported, the sampling frame was not intended to constitute an exhaustive census of all microsurgery-related publications. Articles published in multidisciplinary, oncologic, transplantation, regional, non–PubMed-indexed journals, or conference proceedings may therefore have been missed. To improve transparency and reproducibility, we provide the complete predefined journal list in Table S1, encompassing both core microsurgery and reconstructive surgery journals and adjacent specialty journals. Accordingly, the reported findings and projections should be interpreted as trends within this predefined journal sampling frame rather than as a complete representation of the entire global microsurgery literature.

8. Construction of Thematic Categories The rationale for consolidating keywords into five thematic domains should be described in greater detail. Potential overlap between categories should also be addressed, particularly where individual articles may reasonably fit more than one thematic area. The manuscript should additionally specify which categories were excluded as peripheral and explain the criteria used for exclusion.

Reply: We thank the reviewer for this helpful comment. We agree that the construction of the thematic domains required greater methodological detail. We therefore expanded the Methods section to explain that the five domains were defined *a priori* by consolidating conceptually related keywords into clinically interpretable categories relevant to microsurgical practice and research.

The domains were selected to capture the major recurring areas of microsurgical scholarship while avoiding excessively narrow categories with sparse annual counts. Articles matching more than one domain were reviewed according to their principal study objective, operative application, and dominant terminology in the title and abstract to minimize double counting and preserve interpretability. Ambiguous records were adjudicated by the clinical investigators.

We also clarified that peripheral categories were excluded when they had limited direct relevance to reconstructive microsurgery, insufficient annual frequency for stable forecasting, substantial conceptual overlap with a broader retained domain, or inconsistent temporal signal across the study period.

Revised manuscript text:

Construction of Thematic Domains

For thematic forecasting, individual keywords and related procedural terms were consolidated a priori into five clinically interpretable domains: (i) general free flap–based research, (ii) replantation and limb salvage, (iii) lymphatic microsurgery, (iv) technological and instrumental innovation, and (v) research methodology and outcomes. These domains were selected to represent the major recurring areas of microsurgical scholarship while maintaining sufficient annual publication counts for temporal modeling.

Conceptually related terms were grouped according to their dominant clinical or research application. Articles matching terms from more than one domain were reviewed on the basis of the principal study objective, operative procedure, and dominant emphasis of the title and abstract. Ambiguous records were adjudicated by the clinical investigators to assign the most appropriate primary thematic category and minimize double counting.

Categories were excluded from thematic forecasting when they were peripheral to the central scope of reconstructive microsurgery, had insufficient annual publication frequency for stable modeling, substantially overlapped with a broader retained domain, or lacked a consistent temporal signal across the study period.

9. Consistency of Terminology The terms "forecast," "forecasting," "projection," and "prediction" are used interchangeably throughout the manuscript. These terms have different methodological implications and should be standardized throughout the paper.

Reply: We thank the reviewer for this comment. We agree that consistent terminology is important, particularly because the study extrapolates historical publication trends rather than providing deterministic predictions of future scientific activity. We therefore reviewed the manuscript throughout and standardized the terminology.

The term *forecasting* is now used to describe the statistical modeling process, whereas *projection* refers to the resulting estimates of publication activity through 2030. The term *prediction* and related deterministic language were removed or replaced except where used in a general methodological context or in the formal name of a statistical concept. This revision is consistent with our broader effort to clarify that the reported estimates are conditional on continuation of the observed historical trends.

10. Treatment of Sparse Country Level Data The manuscript reports country level analyses despite very low publication counts in certain regions and income groups. Additional information should be provided regarding how countries with sparse data were handled in the forecasting analyses and whether any thresholds were applied.

Reply: We thank the reviewer for this important comment. We agree that sparse country-level publication counts can produce unstable national estimates and should be handled transparently. We have clarified the treatment of sparse data in the revised Methods and Limitations sections.

All countries with verifiable first-author affiliation data were retained for descriptive global summaries and World Bank income-group analyses, because excluding low-output countries from these aggregate analyses would further underrepresent regions with limited indexed microsurgical publication activity. However, individual country-level forecast interpretation was restricted to countries with sufficient publication activity to support stable trend visualization and meaningful interpretation. Countries with very sparse or intermittent publication counts were not emphasized as individual national trajectories; instead, they contributed to aggregate income-group and global estimates.

We also clarified that country-specific projections, particularly for low-output countries or regions, should be interpreted cautiously. These estimates are exploratory and may be sensitive to isolated publications, zero-count years, and small changes in annual output. This limitation is now explicitly acknowledged in the manuscript.

Added Methods text:

> *All countries with verifiable first-author affiliation data were retained for descriptive global summaries and World Bank income-group analyses. This approach avoided excluding low-output regions from aggregate estimates of global microsurgical research activity. However, because annual country-level publication counts were sparse for several countries, individual national projections were interpreted only for countries with sufficient publication activity to support stable trend visualization. Countries with very low or intermittent publication counts contributed to aggregate regional and income-group analyses but were not emphasized as stand-alone national forecasting results.*

Added Limitations text:

Country-level estimates were constrained by sparse publication activity in several regions and income groups. Although all countries with verifiable first-author affiliation data were retained in descriptive and income-group analyses, individual national projections for low-output countries may be unstable because of isolated publications, zero-count years, and limited temporal signal. Therefore, country-specific projections should be interpreted as exploratory, particularly for countries with low cumulative output. The absence of a stable country-level trajectory should not be interpreted as an absence of microsurgical research capacity or clinical activity.

Reviewer 3 Comments:

This study presents a dual artificial intelligence framework that integrates automated PubMed metadata extraction with contextual text mining to identify microsurgery-related publications and forecast future research trends. Based on 11,561 validated publications from 20 journals (2010-2024), the authors predict continued growth in global microsurgical research through 2030, with increasing contributions from upper-middle-income countries, particularly in Asia, and rapid expansion of lymphatic microsurgery and technological innovation, while replantation and limb salvage research is expected to plateau. I have following major comments: 1) The forecasting methodology requires substantially stronger validation. Although several forecasting models were compared, the manuscript reports only in-sample goodness-of-fit (R^2, RMSE, MAE, AIC) without true out-of-sample or temporal validation. Since the objective is prediction rather than retrospective fitting, the authors should perform rolling-origin validation, train-test temporal splitting, or back-testing to demonstrate that the selected linear regression model can reliably forecast unseen future data.

Reply: We thank the reviewer for this important methodological comment. This concern was also raised by Reviewer 2, and we have revised the manuscript accordingly. We agree that in-sample goodness-of-fit metrics alone are insufficient to support forecasting performance. Therefore, we added two temporal out-of-sample validation analyses.

First, we performed rolling-origin expanding-window validation, in which models were fit on earlier years and evaluated against held-out observations at one-, two-, and three-year forecast horizons. Second, we performed a fixed temporal holdout analysis, training models on 2010–2019 and evaluating predictions against observed publication counts from 2020–2024. Out-of-sample performance was summarized using RMSE, MAE, MAPE, and 95% prediction-interval coverage.

These analyses supported short-horizon stability of the linear projection model. In rolling-origin validation, one-year-ahead linear forecasts achieved RMSE 65.1, MAE 52.8, and MAPE 6.6% across seven validation origins; pooled across one- to three-year horizons, RMSE was 54.2, MAE 44.4, and MAPE 5.3%. In the fixed temporal holdout analysis, the linear model achieved RMSE 40.5, MAE 36.2, MAPE 4.0%, and 95% prediction-interval coverage of 100%.

We also added residual diagnostics for the selected linear model. Full-sample diagnostics demonstrated $R^2 = 0.870$, Durbin–Watson 1.78, Ljung–Box $p = 0.218$, Shapiro–Wilk $p = 0.316$, and Breusch–Pagan $p = 0.425$, without evidence of statistically significant residual autocorrelation, non-normality, or heteroscedasticity. Finally, we revised the manuscript terminology to emphasize

that the 2025–2030 estimates are trend-based projections under observed historical patterns rather than deterministic predictions of future scientific activity.

2)The study's novelty is somewhat overstated. While the integration of automated bibliometric extraction and AI-assisted classification is technically useful, the forecasting itself relies primarily on conventional linear regression rather than advanced AI or machine learning algorithms. Referring to the framework as an "AI-powered forecasting system" may overstate the methodological innovation, and the manuscript should better distinguish between AI-assisted data processing and statistical forecasting.

Reply: We thank the reviewer for this important comment. We agree that the original terminology may have overstated the role of artificial intelligence in the forecasting component. This concern was also raised by the previous reviewers, and we have revised the manuscript accordingly.

Throughout the revised manuscript, we no longer describe the framework as an "AI-powered forecasting system" or a "dual-AI framework." Instead, we use more precise terminology, including *"AI-enabled dual-component framework"* and *"AI-assisted forecasting framework."* The revised wording explicitly distinguishes between the two methodological components: AI-enabled automated metadata extraction and contextual text classification, followed by conventional statistical forecasting using linear regression, quadratic regression, ARIMA, and Holt's exponential smoothing.

We now state clearly that the forecasting models themselves are not advanced AI or machine-learning algorithms and that the novelty of the study does not lie in developing a new neural-network architecture or black-box predictive model. Rather, the contribution lies in integrating scalable automated literature extraction, contextual microsurgery classification, and transparent statistical forecasting into a reproducible and clinically interpretable workflow for surgeons, clinician-scientists, and interdisciplinary medical research teams.

To reinforce this distinction, we revised the title, Methods, Discussion, Conclusion, and terminology throughout the manuscript. The title was changed from:

> *"Predicting the Future of Microsurgical Science: An AI Framework for Global Publication Forecasting"*

to:

> *"AI-Assisted Forecasting in Microsurgery: A Dual-Component Framework for Global*

Publication Trends."

Revised manuscript text:

> *The framework is described as AI-enabled rather than AI-autonomous. Its AI-enabled component lies in the scalable extraction, structuring, and contextual classification of a large biomedical literature corpus. The subsequent forecasting stage uses conventional statistical models, including linear regression, quadratic regression, autoregressive integrated moving average, and Holt's exponential smoothing. These models were selected for transparency, reproducibility, and interpretability rather than algorithmic complexity. Accordingly, the methodological contribution of this study is the integration of AI-enabled literature processing with clinically interpretable statistical forecasting, not the development of a novel machine-learning architecture.*

3) The article selection strategy may introduce substantial selection bias. Restricting the analysis to only 20 predefined journals excludes a considerable proportion of microsurgical publications published in multidisciplinary, oncology, orthopedic, vascular, transplantation, and general surgery journals. Consequently, the presented forecasts may not accurately represent global microsurgical research activity. The rationale for journal selection should be better justified, and sensitivity analyses including broader journal coverage would strengthen the conclusions.

Reply: We thank the reviewer for this important comment. This concern was also raised by the previous reviewers, and we revised the manuscript accordingly. We expanded the Methods section to provide a clearer rationale for journal selection, including the predefined eligibility criteria, the range of microsurgery-practicing specialties represented, and the inclusion of both core microsurgery journals and adjacent surgical disciplines.

We also explicitly acknowledge that the 20-journal sampling frame was designed to provide a broad, clinically relevant, and reproducible representation of microsurgical scholarship rather than an exhaustive census of all publications in the field. Consequently, relevant articles published in multidisciplinary, oncologic, transplantation, general surgery, regional, or non–PubMed-indexed journals may not have been captured.

A broader-coverage sensitivity analysis would require redefining the study sampling frame and repeating the entire extraction, classification, validation, and forecasting workflow across a

substantially larger and more heterogeneous journal corpus. Such an analysis would therefore constitute a materially different study rather than a conventional sensitivity analysis of the present dataset. We instead strengthened the manuscript by clearly defining the sampling frame, moderating the generalizability of the conclusions, and adding a dedicated statement in the *Limitations* section.

Added Limitations text:

> *Third, journal selection and PubMed indexing impose an important scope constraint. Although the 20 journals were selected a priori to represent the principal specialties in which microsurgical techniques are practiced and reported, the sampling frame was not intended to constitute an exhaustive census of all microsurgery-related publications. Articles published in multidisciplinary, oncologic, transplantation, regional, non–PubMed-indexed journals, or conference proceedings may therefore have been missed. To improve transparency and reproducibility, we provide the complete predefined journal list in Table S1, encompassing both core microsurgery and reconstructive surgery journals and adjacent specialty journals. Accordingly, the reported findings and projections should be interpreted as trends within this predefined journal sampling frame rather than as a complete representation of the entire global microsurgery literature.*

4) The rapid integration of artificial intelligence into medical research has been accompanied by substantial advances in AI-assisted imaging analysis, predictive modeling, and translational applications across multiple clinical disciplines. The authors are encouraged to add the following related reference titled "Advancements in Imaging Technologies and AI Integration for Neurodegenerative Disease Management: A Narrative Review", as it provides a comprehensive overview of AI methodologies and may further strengthen the discussion regarding the broader applications and future potential of AI-driven research frameworks in medicine.

Reply: We thank the reviewer for this suggestion. We have added the proposed reference to the Introduction where the broader applications of AI-assisted methods across medical research and clinical disciplines are considered.

5) The thematic classification methodology lacks sufficient transparency and reproducibility. Although the authors report >98% reproducibility after manual review, important details are missing, including the size and construction of the keyword dictionary, handling of ambiguous articles,

inter-rater agreement statistics (e.g., Cohen's $\varkappa$), classifier performance metrics (precision, recall, F1-score), and examples of misclassified records. Without these details, it is difficult to assess the robustness and reproducibility of the AI classification framework.

Reply: We thank the reviewer for this important methodological comment. This concern was also raised by Reviewer 2, and we have substantially revised the classification Methods and Results sections accordingly. We replaced the previous statement of >98% "reproducibility" with a more precise validation description based on agreement with a human-reviewed reference standard.

In the revised manuscript, we now specify that the keyword taxonomy contained 86 terms/stems across 9 thematic domains: general microsurgery terms, vascular/anastomotic keywords, neural microsurgery, replantation and limb salvage, lymphatic microsurgery, flap-based reconstruction, training/simulation/education, technological and instrumental innovation, and research methodology/outcomes. Terms were applied to titles and abstracts using case-insensitive matching with wildcard/stem expansion where appropriate. Ambiguous articles, records with limited abstracts, and records spanning multiple reconstructive domains were adjudicated using a primary-focus rule based on title and abstract content.

We also added a detailed validation analysis. A stratified validation sample of 4,441 records, corresponding to 5.3% of the 83,133 classified source records, was sampled by classifier label, publication year, and journal. Against the human-reviewed reference standard, the classifier achieved 95.2% agreement (95% CI, 94.5%–95.8%) and Cohen's κ of 0.826. The confusion matrix included 627 true positives, 112 false positives, 101 false negatives, and 3,601 true negatives. Sensitivity was 86.1%, specificity 97.0%, positive predictive value 84.8%, negative predictive value 97.3%, false-positive rate 3.0%, false-negative rate 13.9%, and F1 score 0.855.

Finally, we added a brief misclassification/error analysis. False-positive cases most commonly involved adjacent non-microsurgical uses of terms such as "anastomosis" or "microvascular," whereas false-negative cases generally involved articles using indirect free-flap, perforator-flap, lymphatic, or replantation terminology. These additions improve the transparency and reproducibility of the thematic classification process.

Added Methods text:

> *The keyword taxonomy contained 86 terms/stems across 9 thematic domains: general*

microsurgery terms, vascular/anastomotic keywords, neural microsurgery, replantation and limb salvage, lymphatic microsurgery, flap-based reconstruction, training/simulation/education, technological and instrumental innovation, and research methodology/outcomes. Terms were applied to article titles and abstracts using case-insensitive matching with wild-card/stem expansion where appropriate. Articles with only incidental keyword matches were classified according to primary study focus rather than keyword presence alone. Ambiguous records, records with limited abstracts, and records spanning multiple reconstructive domains were adjudicated using a title/abstract primary-focus rule.

Classification validation used a stratified sample drawn from all records that underwent relevance classification. Sampling was stratified by classifier label, publication year, and journal to represent both predicted relevant and predicted non-relevant records across the full study period. The final human-reviewed adjudicated label served as the reference standard for performance metrics.

Added Results text:

Classification accuracy was evaluated using a stratified validation sample of 4,441 records, representing 5.3% of the 83,133 classified source records. The sample was stratified by classifier label, publication year, and journal. Against the human-reviewed reference standard, the classifier achieved 95.2% agreement (95% CI, 94.5%–95.8%) and Cohen's κ of 0.826. The confusion matrix included 627 true positives, 112 false positives, 101 false negatives, and 3,601 true negatives. Sensitivity was 86.1%, specificity 97.0%, positive predictive value 84.8%, negative predictive value 97.3%, false-positive rate 3.0%, false-negative rate 13.9%, and F1 score 0.855.

The 213 classifier-reference discordant records included 112 false positives and 101 false negatives. False-positive classifications most commonly reflected adjacent non-microsurgical uses of terms such as "anastomosis" or "microvascular." False-negative classifications generally involved records using indirect or nonstandard terminology for free-flap reconstruction, perforator-flap surgery, lymphatic microsurgery, or replantation. These findings were used to characterize the limitations of the taxonomy-based classifier and to guide interpretation of the final dataset.

6) Several conclusions regarding future geographic leadership, research capacity, and thematic evolution appear stronger than the presented evidence supports. The forecasts are based solely on historical publication counts and do not account for major external factors such as funding policies, geopolitical changes, technological breakthroughs, publication practice changes, or disruptions similar to the COVID-19 pandemic. These assumptions should be discussed more explicitly, and the conclusions should be moderated to acknowledge the uncertainty inherent in long-term bibliometric forecasting.

Reply: We thank the reviewer for this important comment. This concern was also raised by the previous reviewers, and we revised the manuscript accordingly. Specifically, we moderated statements regarding future geographic leadership, research capacity, innovation, and thematic evolution and standardized the terminology to emphasize that the reported estimates are conditional projections rather than deterministic predictions.

We also expanded the *Limitations* section to state explicitly that the models are based on historical publication trajectories and cannot account for future changes in funding, research policy, geopolitical conditions, publication practices, workforce composition, journal indexing, or disruptive technological developments. In addition, we clarified that publication volume reflects scholarly activity and should not be interpreted as a direct measure of scientific quality, clinical significance, innovation, or future leadership.

Added Limitations text:

> *Fourth, the forecasting models were derived from historical publication trajectories and therefore provide conditional projections rather than validated predictions of future scientific activity. Although multiple forecasting approaches were compared and 95% confidence intervals were reported, the models assume that the underlying temporal structure observed from 2010 to 2024 will remain sufficiently stable over the projection period. They cannot account for future disruptions or structural changes, including shifts in funding priorities, research policy, geopolitical conditions, journal indexing, publication practices, workforce composition, or major technological breakthroughs. Moreover, publication volume should be interpreted as a measure of scholarly activity rather than a direct indicator of scientific quality, innovation, clinical significance, or future leadership. Accordingly, projections through 2030 represent scenario-based estimates under*

continuation of observed historical trends and should not be interpreted as deterministic forecasts of the future direction or impact of microsurgical science.

References

[1] Georgios Karamitros, Georgios Bouloukakis, Emmanuel Giannas, Gregory A Lamaris, Wesley P Thayer, Galen Perdikis, William C Lineaweaver, and Patrick Assi. Predicting the future of aesthetic surgery: An artificial intelligence framework for global publication forecasting. *Aesthetic Surgery Journal*, 46(7):773–785, 2026.

[2] Sofoklis Goulas and Georgios Karamitros. How to harness the power of web scraping for medical and surgical research: An application in estimating international collaboration. *World journal of surgery*, 48(6):1297–1300, 2024.

[3] Georgios Karamitros and Sofoklis Goulas. Human capital and productivity in surgery research across the globe: a big data analysis using artificial intelligence. *Journal of Surgical Research*, 303:731–743, 2024.

[4] Georgios Karamitros and Sofoklis Goulas. Women representation in plastic surgery across the globe: a cross-sectional study of human capital and research output using artificial intelligence. *Journal of Plastic, Reconstructive & Aesthetic Surgery*, 81:91–93, 2023.

[5] Georgios Karamitros and Sofoklis Goulas. Human capital and productivity in plastic surgery research across nations. *Aesthetic plastic surgery*, 47(4):1644–1657, 2023.

[6] Sofoklis Goulas and Georgios Karamitros. Human capital and productivity in surgery research during a pandemic: an artificial intelligence approach. *Br J Surg.*, 110(9):1218–1220, 2023.

[7] Georgios Karamitros, Georgios Bouloukakis, Gregory A Lamaris, Wesley P Thayer, Galen Perdikis, Feng Zhang, and William C Lineaweaver. Artificial intelligence-driven mapping of global microsurgery research: Dual automation for data and classification. *Journal of Surgical Research*, 322:178–195, 2026.

[8] Christopher Meaney, Rahim Moineddin, Teja Voruganti, Mary Ann O'Brien, Paul Krueger, and Frank Sullivan. Text mining describes the use of statistical and epidemiological methods in published medical research. *Journal of Clinical Epidemiology*, 74:124–132, 2016.

[9] Paul Thompson, Riza Theresa Batista-Navarro, Georgios Kontonatsios, Jacob Carter, Elizabeth Toon, John McNaught, Carsten Timmermann, Michael Worboys, and Sophia Ananiadou. Text mining the history of medicine. *PloS One*, 11(1):e0144717, 2016.

[10] Anne Kao and Steve R Poteet. *Natural language processing and text mining.* Springer Science & Business Media, 2007.

[11] Panos Alexopoulos. *Semantic modeling for data.* O'Reilly Media, 2020.

[12] André Luis Santiago Maia and Francisco de AT de Carvalho. Holt's exponential smoothing and neural network models for forecasting interval-valued time series. *International Journal of Forecasting*, 27(3):740–759, 2011.

[13] Hirotugu Akaike. Akaike's information criterion. In *International encyclopedia of statistical science*, pages 41–42. Springer, 2025.

[14] Georgios Karamitros, Michael P Grant, and Gregory A Lamaris. Associations in medical research can be misleading: A clinician's guide to causal inference. *Journal of Surgical Research*, 310:145–154, 2025.

[15] Bernard Bercu and Frédéric Proïa. A sharp analysis on the asymptotic behaviorof the durbin–watson statistic for the first-order autoregressive process. *ESAIM: Probability and Statistics*, 17:500–530, 2013.

[16] Zofia Hanusz, Joanna Tarasinska, and Wojciech Zielinski. Shapiro–wilk test with known mean. *REVSTAT-statistical Journal*, 14(1):89–100, 2016.

[17] Andreea G Halunga, Chris D Orme, and Takashi Yamagata. A heteroskedasticity robust breusch–pagan test for contemporaneous correlation in dynamic panel data models. *Journal of econometrics*, 198(2):209–230, 2017.

[18] Amy Lapidow and Paige Scudder. Shared first authorship. *J Med Libr Assoc.*, 107(4):618, 2019.

[19] Edilson A Corrêa Jr, Filipi N Silva, Luciano da F Costa, and Diego R Amancio. Patterns of Authors' Contribution in Scientific Manuscripts. *J Informetr.*, 11(2):498–510, 2017.

[20] Rodrigo Costas and María Bordons. Do age and professional rank influence the order of authorship in scientific publications? some evidence from a micro-level perspective. *Scientometrics*, 88(1):145–161, 2011.

[21] Georgios Karamitros and Sofoklis Goulas. Human Capital and Productivity in Plastic Surgery Research Across Nations. *Aesthetic Plast Surg*, pages 1–14, 2022.

[22] Andrew E Liechty, James R Sherpa, Jonathan S Trejo, Mackenzie M French, Cameron J Kneib, Daniel Y Cho, and Jeffrey B Friedrich. Globalization of Plastic and Reconstructive Surgery: A Continent, Country, and State-level Analysis of Publications. *Plast Reconstr Surg Glob Open.*, 8(11), 2020.

[23] Robert J Barro. Human Capital and Growth. *Am Econ Rev.*, 91(2):12–17, 2001.

[24] Eugene Garfield. The history and meaning of the journal impact factor. *JAMA*, 295(1):90–93, 2006.

[25] Jessica D Blum, Anchith Kota, Dillan F Villavisanis, Daniel Y Cho, Jordan W Swanson, Scott P Bartlett, and Jesse A Taylor. The Impact of Senior Author Profile on Publication Level of Evidence in Plastic and Reconstructive Surgery. *Plast Reconstr Surg Glob Open.*, 10(9):e4506, 2022.

[26] Georgios Karamitros and Sofoklis Goulas. Human Capital and Productivity in Plastic Surgery Research During COVID-19: An Artifical Intelligence Approach. *J Plast Reconstr Aesthet Surg*, 2023.

[27] Georgios Karamitros and Sofoklis Goulas. Women Representation in Plastic Surgery Across the Globe: A Cross-sectional Study of Human Capital and Research Output Using Artifical Intelligence. *J Plast Reconstr Aesthet Surg*, 2023.
