# Supplementary material for: AI-assisted forecasting in microsurgery: A dual-component framework for global publication trends
Source: PLoS One. 2026 Aug 28;21(8):e0357186. doi: 10.1371/journal.pone.0357186 (PMC13524238; doi:10.1371/journal.pone.0357186)
Supplement: Peer review history

# Peer review: Decision Letter 0

**DOI:** 10.1371/journal.pone.0357186.r001

**Authors:** Xiaoen Wei

28 Jun 2026

Dear Dr. Karamitros,

Thank you for submitting your manuscript to PLOS ONE. After careful consideration, we feel that it has merit but does not fully meet PLOS ONE’s publication criteria as it currently stands. Therefore, we invite you to submit a revised version of the manuscript that addresses the points raised during the review process.

Please submit your revised manuscript by Aug 27 2026 11:59PM. If you will need more time than this to complete your revisions, please reply to this message or contact the journal office at plosone@plos.org. When you're ready to submit your revision, log on to https://www.editorialmanager.com/pone/ and select the 'Submissions Needing Revision' folder to locate your manuscript file.

- A letter that responds to each point raised by the academic editor and reviewer(s). You should upload this letter as a separate file labeled 'Response to Reviewers'.
- A marked-up copy of your manuscript that highlights changes made to the original version. You should upload this as a separate file labeled 'Revised Manuscript with Track Changes'.
- An unmarked version of your revised paper without tracked changes. You should upload this as a separate file labeled 'Manuscript'.

If you would like to make changes to your financial disclosure, please include your updated statement in your cover letter. Guidelines for resubmitting your figure files are available below the reviewer comments at the end of this letter.

If applicable, we recommend that you deposit your laboratory protocols in protocols.io to enhance the reproducibility of your results. Protocols.io assigns your protocol its own identifier (DOI) so that it can be cited independently in the future. For instructions see: https://journals.plos.org/plosone/s/submission-guidelines#loc-laboratory-protocols. Additionally, PLOS ONE offers an option for publishing peer-reviewed Lab Protocol articles, which describe protocols hosted on protocols.io. Read more information on sharing protocols at https://plos.org/protocols?utm_medium=editorial-email&utm_source=authorletters&utm_campaign=protocols.

As the corresponding author, your ORCID iD is verified in the submission system and will appear in the published article. PLOS supports the use of ORCID, and we encourage all coauthors to register for an ORCID iD and use it as well. Please encourage your coauthors to verify their ORCID iD within the submission system before final acceptance, as unverified ORCID iDs will not appear in the published article. *Only*  the individual author can complete the verification step; PLOS staff *cannot*  verify ORCID iDs on behalf of authors.

We look forward to receiving your revised manuscript.

Kind regards,

Xiaoen Wei

Academic Editor

PLOS One

Journal Requirements:

When submitting your revision, we need you to address these additional requirements.

1. Please ensure that your manuscript meets PLOS ONE's style requirements, including those for file naming. The PLOS ONE style templates can be found at

https://journals.plos.org/plosone/s/file?id=wjVg/PLOSOne_formatting_sample_main_body.pdf and

https://journals.plos.org/plosone/s/file?id=ba62/PLOSOne_formatting_sample_title_authors_affiliations.pdf

2. Please remove your figures from within your manuscript file, leaving only the individual TIFF/EPS image files, uploaded separately. These will be automatically included in the reviewers’ PDF.

3. If the reviewer comments include a recommendation to cite specific previously published works, please review and evaluate these publications to determine whether they are relevant and should be cited. There is no requirement to cite these works unless the editor has indicated otherwise.

[Note: HTML markup is below. Please do not edit.]

Reviewers' comments:

Reviewer's Responses to Questions

**Comments to the Author**

1. Is the manuscript technically sound, and do the data support the conclusions?

Reviewer #1: Partly

Reviewer #2: Partly

Reviewer #3: Partly

**********

2. Has the statistical analysis been performed appropriately and rigorously?

Reviewer #1: No

Reviewer #2: No

Reviewer #3: I Don't Know

**********

3. Have the authors made all data underlying the findings in their manuscript fully available?

The PLOS Data policy

Reviewer #1: Yes

Reviewer #2: No

Reviewer #3: No

**********

4. Is the manuscript presented in an intelligible fashion and written in standard English?

Reviewer #1: Yes

Reviewer #2: Yes

Reviewer #3: Yes

**********

Reviewer #1: 1. The abstract is poorly structured and does not adequately summarize the study. It should clearly describe: the AI/ML models used, the forecasting methods employed, Dataset size, Evaluation metrics, Key quantitative findings and conclusions.

2. The Introduction section is too brief and requires substantial expansion. The authors should clearly discuss: Existing research gaps, Motivation for the study, Research objectives/questions, Novel contributions of the proposed framework.

3. A dedicated Literature Review / Related Work section is missing. The manuscript should discuss existing bibliometric forecasting studies, AI-based trend prediction approaches, and related microsurgery bibliometric analyses. The limitations of previous studies and the novelty of the proposed work should be explicitly highlighted.

4. The manuscript repeatedly refers to a "dual-AI framework"; however, the technical description of the AI components is insufficient. The authors should clearly explain: Which AI/NLP models were used, How article classification was performed, Model training and validation procedures, Why the framework qualifies as an AI-based system.

5. In Section 2.2, the authors state that Table S1 contains the list of included journals. However, Table S1 contains research intensity forecasting results instead of journal information. This inconsistency should be corrected and all supplementary materials should be carefully verified.

6. The dataset description should be summarized in a dedicated table, including: Total records retrieved, Number of journals included, Records removed due to missing metadata, Records excluded after classification, Final dataset size, Study period.

7. Although source code has been made available, the underlying processed dataset is not accessible. The authors should clarify how independent researchers can reproduce the reported findings and results.

8. The manuscript lacks an architecture/workflow diagram of the proposed dual-AI framework. A detailed block diagram illustrating data collection, preprocessing, AI-based classification, forecasting models, and output generation should be included. Insufficient methodological detail about the AI framework.

9. Several references related to AI, text mining, and forecasting methodologies are outdated. More recent studies (2022–2026) should be incorporated.

10. The manuscript should include a dedicated limitations subsection discussing potential biases associated with PubMed-only data collection, article classification, and forecasting assumptions.

11. The manuscript would benefit from professional English language editing to improve readability and clarity.

12. The authors repeatedly claim the use of a "dual-AI framework," but the actual methodology appears to rely primarily on: PubMed data extraction, Text filtering/classification, Linear Regression, Polynomial Regression, ARIMA, Holt Exponential Smoothing.

13. These are mostly conventional statistical and bibliometric techniques. The manuscript should clearly justify why this framework is considered AI-driven rather than a traditional bibliometric forecasting pipeline.

14. The manuscript does not compare its findings against: Previous microsurgery bibliometric analyses, Existing forecasting studies, Similar scientometric research, Without benchmarking against prior work, it is difficult to assess the added value of the proposed framework.

15. The classification framework reports high reproducibility but does not analyze: Misclassified articles, False positives, False negatives, Challenging cases. An error analysis section would strengthen confidence in the methodology.

16. The manuscript frequently uses causal language when only descriptive bibliometric analyses are presented. Statements implying causes of productivity growth should be moderated.

17. Figures and tables are placed collectively at the end of the manuscript, which negatively affects readability and makes it difficult for readers to follow the results and discussion sections. The authors should place all figures and tables within the main text, immediately after or close to their first citation, in accordance with standard scientific writing practices and to improve manuscript flow and comprehension.

18. The manuscript appears to present bibliometric analysis, text filtering, and statistical forecasting under the umbrella of "Artificial Intelligence." However, the actual methodological novelty is limited, and the AI component is not sufficiently demonstrated or validated.

19. Several figure captions are excessively long and contain interpretation rather than description. Interpretive discussion should be moved to the Results or Discussion sections.

20. The rationale for selecting only 20 journals is insufficient. The authors should explain how these journals were identified and justify whether they adequately represent the global microsurgery literature.

Reviewer #2: This manuscript presents a bibliometric analysis of microsurgery publications using automated article retrieval, thematic classification, and forecasting methods. The dataset is substantial, and the topic is relevant to surgical research and research evaluation. The study also addresses an area that has received comparatively limited attention within the microsurgical literature. However, several aspects of the methodology are insufficiently described, making it difficult to fully assess the validity, reproducibility, and interpretation of the principal findings. In particular, the classification framework, forecasting methodology, validation procedures, and data availability require substantial clarification before the manuscript can be considered for publication.

Major Concerns

1. Clarification of the "Dual-AI" Framework

The manuscript repeatedly describes the methodology as a "dual-AI framework." However, based on the methods currently presented, the classification procedure appears to be primarily a rule-based keyword filtering approach using terms such as microsurg, free flap, perforator, replantation, and lymphatic. Automated data retrieval and keyword classification do not, by themselves, constitute artificial intelligence or machine learning.

If machine learning, natural language processing, large language models, or other AI methods were used, these methods should be described in sufficient detail, including model architecture, training procedures, validation methods, and implementation details. If no such methods were employed, the authors should reconsider the characterization of the framework as AI-based and instead describe it as an automated bibliometric pipeline with keyword-based classification.

2. Forecasting Methodology and Predictive Validity

The forecasting analyses are based on annual publication counts from 2010 through 2024, yielding only fifteen observations for model fitting. This limited time series constrains the reliability of model selection, uncertainty estimation, and long-range extrapolation through 2030.

The manuscript compares multiple forecasting approaches using R², RMSE, MAE, and AIC and selects linear regression as the preferred model. However, all reported performance metrics appear to be based on the same data used for model fitting. No temporal holdout validation, rolling-origin validation, or other assessment of out-of-sample performance is presented.

Consequently, the analysis demonstrates model fit but does not demonstrate forecasting accuracy. The authors should consider providing a temporal validation strategy (for example, fitting models on earlier years and evaluating performance on later years) or explicitly acknowledge that the reported results represent projections under observed historical trends rather than validated forecasts.

In addition, the rationale for selecting linear regression over alternative models should be expanded. Although the linear model achieved the lowest AIC, the differences between models appear modest. Residual diagnostics, assessment of autocorrelation, and discussion of model assumptions would strengthen confidence in the chosen approach.

3. Classification Validation Requires Additional Detail

The manuscript reports greater than 98% reproducibility based on manual review of approximately 5% of the dataset. However, the validation process is not described in sufficient detail.

The authors should report:

• The exact validation sample size.

• The stratification procedure used for sampling.

• A confusion matrix.

• Sensitivity and specificity.

• Positive and negative predictive values.

• False-positive and false-negative rates.

• Inter-rater agreement statistics (e.g., Cohen's kappa).

In addition, the manuscript appears to use the terms "agreement" and "reproducibility" interchangeably. These concepts are not identical and should be clearly distinguished. If the reported value refers to agreement with manual review, this should be stated explicitly.

4. Human Capital Proxy Requires Further Justification

The study uses first author publication counts as a proxy for national research capacity and human capital. Although references are provided to support this approach, first authorship remains an indirect surrogate measure with recognized limitations.

Authorship conventions vary across disciplines, countries, and collaborative structures. Multiple first authorship is increasingly common, and first author attribution may not fully capture mentorship, leadership, or collaborative contributions.

The limitations associated with this proxy should be discussed more thoroughly. A sensitivity analysis using an alternative authorship metric would further strengthen confidence that the reported findings are not dependent on the specific choice of proxy.

5. Data Availability and Reproducibility

The public availability of the code repository is commendable and supports reproducibility. However, the analytical dataset used to generate the reported results does not appear to be publicly available.

Because the study is based on publicly available PubMed metadata rather than protected human subject data, the rationale for withholding the analytical dataset is unclear. PLOS ONE places strong emphasis on data availability and reproducibility.

At a minimum, the authors should consider providing the aggregated annual publication counts by country and thematic category that underlie the forecasting analyses. If sharing the full article level dataset is not possible, the reasons should be clearly explained.

6. Interpretation of Findings and Strength of Conclusions

Several statements throughout the manuscript appear stronger than the available evidence supports. Terms such as "predicting the future of microsurgical science," "anticipating innovation," and similar language imply a degree of predictive certainty that exceeds what can be established from publication count projections.

Publication volume is a useful indicator of research activity but should not be interpreted as a direct measure of innovation, scientific impact, clinical significance, or future leadership.

The discussion and conclusion should be revised to distinguish projections from predictions and to acknowledge more explicitly the limitations inherent in extrapolating historical publication trends.

Minor Concerns

7. Journal Selection Process

The manuscript states that twenty journals were included but provides limited information regarding how these journals were selected. Greater transparency regarding journal identification, inclusion criteria, and potential exclusions would strengthen the methodological rigor of the study.

8. Construction of Thematic Categories

The rationale for consolidating keywords into five thematic domains should be described in greater detail. Potential overlap between categories should also be addressed, particularly where individual articles may reasonably fit more than one thematic area.

The manuscript should additionally specify which categories were excluded as peripheral and explain the criteria used for exclusion.

9. Consistency of Terminology

The terms "forecast," "forecasting," "projection," and "prediction" are used interchangeably throughout the manuscript. These terms have different methodological implications and should be standardized throughout the paper.

10. Treatment of Sparse Country Level Data

The manuscript reports country level analyses despite very low publication counts in certain regions and income groups. Additional information should be provided regarding how countries with sparse data were handled in the forecasting analyses and whether any thresholds were applied.

Reviewer #3: This study presents a dual artificial intelligence framework that integrates automated PubMed metadata extraction with contextual text mining to identify microsurgery-related publications and forecast future research trends. Based on 11,561 validated publications from 20 journals (2010-2024), the authors predict continued growth in global microsurgical research through 2030, with increasing contributions from upper-middle-income countries, particularly in Asia, and rapid expansion of lymphatic microsurgery and technological innovation, while replantation and limb salvage research is expected to plateau.

I have following major comments: 1) The forecasting methodology requires substantially stronger validation. Although several forecasting models were compared, the manuscript reports only in-sample goodness-of-fit (R², RMSE, MAE, AIC) without true out-of-sample or temporal validation. Since the objective is prediction rather than retrospective fitting, the authors should perform rolling-origin validation, train-test temporal splitting, or back-testing to demonstrate that the selected linear regression model can reliably forecast unseen future data. 2)The study's novelty is somewhat overstated. While the integration of automated bibliometric extraction and AI-assisted classification is technically useful, the forecasting itself relies primarily on conventional linear regression rather than advanced AI or machine learning algorithms. Referring to the framework as an "AI-powered forecasting system" may overstate the methodological innovation, and the manuscript should better distinguish between AI-assisted data processing and statistical forecasting. 3) The article selection strategy may introduce substantial selection bias. Restricting the analysis to only 20 predefined journals excludes a considerable proportion of microsurgical publications published in multidisciplinary, oncology, orthopedic, vascular, transplantation, and general surgery journals. Consequently, the presented forecasts may not accurately represent global microsurgical research activity. The rationale for journal selection should be better justified, and sensitivity analyses including broader journal coverage would strengthen the conclusions. 4) The rapid integration of artificial intelligence into medical research has been accompanied by substantial advances in AI-assisted imaging analysis, predictive modeling, and translational applications across multiple clinical disciplines. The authors are encouraged to add the following related reference titled “Advancements in Imaging Technologies and AI Integration for Neurodegenerative Disease Management: A Narrative Review”, as it provides a comprehensive overview of AI methodologies and may further strengthen the discussion regarding the broader applications and future potential of AI-driven research frameworks in medicine. 5) The thematic classification methodology lacks sufficient transparency and reproducibility. Although the authors report >98% reproducibility after manual review, important details are missing, including the size and construction of the keyword dictionary, handling of ambiguous articles, inter-rater agreement statistics (e.g., Cohen's κ), classifier performance metrics (precision, recall, F1-score), and examples of misclassified records. Without these details, it is difficult to assess the robustness and reproducibility of the AI classification framework. 6) Several conclusions regarding future geographic leadership, research capacity, and thematic evolution appear stronger than the presented evidence supports. The forecasts are based solely on historical publication counts and do not account for major external factors such as funding policies, geopolitical changes, technological breakthroughs, publication practice changes, or disruptions similar to the COVID-19 pandemic. These assumptions should be discussed more explicitly, and the conclusions should be moderated to acknowledge the uncertainty inherent in long-term bibliometric forecasting.

**********

what does this mean?). If published, this will include your full peer review and any attached files.

If you choose “no”, your identity will remain anonymous but your review may still be made public.

**Do you want your identity to be public for this peer review?**  For information about this choice, including consent withdrawal, please see our Privacy Policy

Reviewer #1: **Yes:**  Muhammad Junaid Asif

Reviewer #2: No

Reviewer #3: No

**********

[NOTE: If reviewer comments were submitted as an attachment file, they will be attached to this email and accessible via the submission site. Please log into your account, locate the manuscript record, and check for the action link "View Attachments". If this link does not appear, there are no attachment files.]

To ensure your figures meet our technical requirements, please review our figure guidelines: https://journals.plos.org/plosone/s/figures

You may also use PLOS’s free figure tool, NAAS, to help you prepare publication quality figures: https://journals.plos.org/plosone/s/figures#loc-tools-for-figure-preparation.

NAAS will assess whether your figures meet our technical requirements by comparing each figure against our figure specifications.

# Author comment: Author response to Decision Letter 1

**DOI:** 10.1371/journal.pone.0357186.r002

17 Jul 2026

We attached a PDF with detailed point-by-poit responses to reviewers.

Attachment Submitted filename: Response to Reviewers.pdf

# Peer review: Decision Letter 1

**DOI:** 10.1371/journal.pone.0357186.r003

**Authors:** Xiaoen Wei

5 Aug 2026

Dear Dr. Karamitros,

Thank you for submitting your manuscript to PLOS ONE. After careful consideration, we feel that it has merit but does not fully meet PLOS ONE’s publication criteria as it currently stands. Therefore, we invite you to submit a revised version of the manuscript that addresses the points raised during the review process.

Please submit your revised manuscript by Sep 04 2026 11:59PM. If you will need more time than this to complete your revisions, please reply to this message or contact the journal office at plosone@plos.org. When you're ready to submit your revision, log on to https://www.editorialmanager.com/pone/ and select the 'Submissions Needing Revision' folder to locate your manuscript file.

- A letter that responds to each point raised by the academic editor and reviewer(s). You should upload this letter as a separate file labeled 'Response to Reviewers'.
- A marked-up copy of your manuscript that highlights changes made to the original version. You should upload this as a separate file labeled 'Revised Manuscript with Track Changes'.
- An unmarked version of your revised paper without tracked changes. You should upload this as a separate file labeled 'Manuscript'.

If you would like to make changes to your financial disclosure, please include your updated statement in your cover letter. Guidelines for resubmitting your figure files are available below the reviewer comments at the end of this letter.

If applicable, we recommend that you deposit your laboratory protocols in protocols.io to enhance the reproducibility of your results. Protocols.io assigns your protocol its own identifier (DOI) so that it can be cited independently in the future. For instructions see: https://journals.plos.org/plosone/s/submission-guidelines#loc-laboratory-protocols. Additionally, PLOS ONE offers an option for publishing peer-reviewed Lab Protocol articles, which describe protocols hosted on protocols.io. Read more information on sharing protocols at https://plos.org/protocols?utm_medium=editorial-email&utm_source=authorletters&utm_campaign=protocols.

As the corresponding author, your ORCID iD is verified in the submission system and will appear in the published article. PLOS supports the use of ORCID, and we encourage all coauthors to register for an ORCID iD and use it as well. Please encourage your coauthors to verify their ORCID iD within the submission system before final acceptance, as unverified ORCID iDs will not appear in the published article. *Only*  the individual author can complete the verification step; PLOS staff *cannot*  verify ORCID iDs on behalf of authors.

We look forward to receiving your revised manuscript.

Kind regards,

Xiaoen Wei

Academic Editor

PLOS One

Journal Requirements:

If the reviewer comments include a recommendation to cite specific previously published works, please review and evaluate these publications to determine whether they are relevant and should be cited. There is no requirement to cite these works unless the editor has indicated otherwise.

Please review your reference list to ensure that it is complete and correct. If you have cited papers that have been retracted, please include the rationale for doing so in the manuscript text, or remove these references and replace them with relevant current references. Any changes to the reference list should be mentioned in the rebuttal letter that accompanies your revised manuscript. If you need to cite a retracted article, indicate the article’s retracted status in the References list and also include a citation and full reference for the retraction notice.

[Note: HTML markup is below. Please do not edit.]

Reviewers' comments:

Reviewer's Responses to Questions

**Comments to the Author**

Reviewer #1: All comments have been addressed

Reviewer #2: All comments have been addressed

Reviewer #3: All comments have been addressed

**********

2. Is the manuscript technically sound, and do the data support the conclusions?

Reviewer #1: Yes

Reviewer #2: Yes

Reviewer #3: Yes

**********

3. Has the statistical analysis been performed appropriately and rigorously?

Reviewer #1: No

Reviewer #2: Yes

Reviewer #3: Yes

**********

4. Have the authors made all data underlying the findings in their manuscript fully available?

The PLOS Data policy

Reviewer #1: Yes

Reviewer #2: Yes

Reviewer #3: Yes

**********

5. Is the manuscript presented in an intelligible fashion and written in standard English?

Reviewer #1: Yes

Reviewer #2: Yes

Reviewer #3: Yes

**********

Reviewer #1: 1. The AI novelty remains overstated: Although the authors now describe the framework more transparently, the system is fundamentally a keyword-based contextual classifier combined with conventional statistical forecasting models (Linear Regression, Polynomial Regression, ARIMA, and Holt's Exponential Smoothing). It should be made clearer throughout the manuscript that this is an AI-assisted bibliometric workflow rather than a novel AI methodology. Several statements in the Abstract, Discussion, and Conclusion still overstate the AI contribution.

2. The contextual classifier is still rule-based rather than machine learning-based: The classification approach relies on a predefined keyword taxonomy with manual adjudication instead of a supervised or deep learning classifier. The manuscript should avoid implying semantic AI capabilities beyond what is actually implemented.

3. Forecasting conclusions remain stronger than the available evidence supports: The study is based on only fifteen annual observations (2010–2024). Although additional validation has been included, forecasts through 2030 should continue to be presented cautiously as exploratory projections rather than predictive evidence.

4. What are the main contributions of your research? Should be mentioned in Intrduction section and Contributions should be novel. the overall structure of the paper should also be defined at the end of introduction section.

5. Many figure legends still contain detailed interpretation and discussion rather than concise figure descriptions. Interpretation should remain in the Results or Discussion sections.

6. Despite previous comments, the figures and tables continue to be presented collectively after the references rather than being embedded near their first citation. This negatively affects readability and should be corrected according to the journal's formatting guidelines.

7. The conclusion claims that the framework "redefines" surgical bibliometrics and represents a major methodological advance. These claims should be moderated to better reflect the actual scope of the presented work.

8. Some statements still employ promotional or subjective language, including terms such as:

"methodological leap"

"redefines surgical bibliometrics"

"unprecedented resolution"

"strategic instrument"

"dynamic system"

These expressions should be replaced with more objective scientific wording that is fully supported by the presented evidence.

9. The authors claim that the proposed framework is transferable to other surgical specialties. This statement should be qualified by briefly discussing what modifications would be required (e.g., journal selection, keyword taxonomy, validation process) before applying the framework to other medical domains.

10. The manuscript would benefit from one final round of professional English language editing to correct minor grammatical inconsistencies, improve sentence flow, and ensure consistent formatting throughout the manuscript.

11. The manuscript would benefit from a brief "Future Work" paragraph suggesting possible enhancements, such as:

Incorporation of transformer-based NLP models or large language models for semantic classification.

Inclusion of additional bibliographic databases (e.g., Scopus, Web of Science).

Integration of citation networks, funding information, and collaboration networks.

External validation in other surgical specialties.

Reviewer #2: Thank you for your careful revision. The manuscript is much stronger than the previous version, and the revisions have addressed the main concerns raised during the first round of review.

The methodological framework is now presented more clearly. In particular, the distinction between the automated literature processing and the statistical forecasting components is easier to follow, and the revised terminology more accurately reflects the scope of the work. The additional methodological detail also improves the transparency of the study and makes the workflow easier to reproduce.

The expanded validation strengthens the manuscript. The temporal validation, residual diagnostics, and more detailed reporting of the classification performance provide a clearer picture of how the framework performs in practice. I also found the discussion of the classification errors useful, as it helps readers understand the limitations of the approach rather than focusing only on its overall performance.

The interpretation of the findings is now more balanced. The manuscript consistently presents the forecasts as projections based on historical trends rather than as predictions of future research activity, and the limitations of the study are acknowledged appropriately. The conclusions are supported by the analyses presented.

Overall, I believe the study is technically sound and meets the publication criteria. The methods are described in sufficient detail to allow reproduction, and the conclusions are supported by the data.

I have only a few minor suggestions before publication.

Minor Comments

1. Please check the numbering of the figures, tables, and supplementary material to ensure that all cross-references are consistent throughout the manuscript.

2. A final proofread would help eliminate the small number of typographical and grammatical inconsistencies that remain.

Reviewer #3: I have reviewed the authors responses and the revised manuscript and believe that the manuscript has been substantially improved and now meets the standards for publication.

**********

what does this mean?). If published, this will include your full peer review and any attached files.

If you choose “no”, your identity will remain anonymous but your review may still be made public.

**Do you want your identity to be public for this peer review?**  For information about this choice, including consent withdrawal, please see our Privacy Policy

Reviewer #1: No

Reviewer #2: No

Reviewer #3: No

**********

[NOTE: If reviewer comments were submitted as an attachment file, they will be attached to this email and accessible via the submission site. Please log into your account, locate the manuscript record, and check for the action link "View Attachments". If this link does not appear, there are no attachment files.]

To ensure your figures meet our technical requirements, please review our figure guidelines: https://journals.plos.org/plosone/s/figures

You may also use PLOS’s free figure tool, NAAS, to help you prepare publication quality figures: https://journals.plos.org/plosone/s/figures#loc-tools-for-figure-preparation.

NAAS will assess whether your figures meet our technical requirements by comparing each figure against our figure specifications.

# Author comment: Author response to Decision Letter 2

**DOI:** 10.1371/journal.pone.0357186.r004

8 Aug 2026

A repsonse to reviewers PDF has been submitted

Attachment Submitted filename: Forecasting microsurgery 2nd responce to reviewers.pdf

# Editor report: Decision Letter 2

**DOI:** 10.1371/journal.pone.0357186.r005

**Authors:** Xiaoen Wei

13 Aug 2026

AI-Assisted Forecasting in Microsurgery: A Dual-Component Framework for Global Publication Trends

PONE-D-26-26132R2

Dear Dr. Karamitros,

We’re pleased to inform you that your manuscript has been judged scientifically suitable for publication and will be formally accepted for publication once it meets all outstanding technical requirements.

Within one week, you’ll receive an e-mail detailing the required amendments. When these have been addressed, you’ll receive a formal acceptance letter and your manuscript will be scheduled for publication.

An invoice will be generated when your article is formally accepted. Please note, if your institution has a publishing partnership with PLOS and your article meets the relevant criteria, all or part of your publication costs will be covered. Please make sure your user information is up-to-date by logging into Editorial Manager at Editorial Manager® and clicking the ‘Update My Information' link at the top of the page. For questions related to billing, please contact billing support.

If your institution or institutions have a press office, please notify them about your upcoming paper to help maximize its impact. If they’ll be preparing press materials, please inform our press team as soon as possible -- no later than 48 hours after receiving the formal acceptance. Your manuscript will remain under strict press embargo until 2 pm Eastern Time on the date of publication. For more information, please contact onepress@plos.org.

Kind regards,

Xiaoen Wei

Academic Editor

PLOS One

Additional Editor Comments (optional):

Reviewers' comments:

# Editor report: Acceptance letter

**DOI:** 10.1371/journal.pone.0357186.r006

**Authors:** Xiaoen Wei

PONE-D-26-26132R2

PLOS One

Dear Dr. Karamitros,

I'm pleased to inform you that your manuscript has been deemed suitable for publication in PLOS One. Congratulations! Your manuscript is now being handed over to our production team.

At this stage, our production department will prepare your paper for publication. This includes ensuring the following:

* All references, tables, and figures are properly cited

* All relevant supporting information is included in the manuscript submission,

* There are no issues that prevent the paper from being properly typeset

You will receive further instructions from the production team, including instructions on how to review your proof when it is ready. Please keep in mind that we are working through a large volume of accepted articles, so please give us a few days to review your paper and let you know the next and final steps.

Lastly, if your institution or institutions have a press office, please let them know about your upcoming paper now to help maximize its impact. If they'll be preparing press materials, please inform our press team within the next 48 hours. Your manuscript will remain under strict press embargo until 2 pm Eastern Time on the date of publication. For more information, please contact onepress@plos.org.

You will receive an invoice from PLOS for your publication fee after your manuscript has reached the completed accept phase. If you receive an email requesting payment before acceptance or for any other service, this may be a phishing scheme. Learn how to identify phishing emails and protect your accounts at https://explore.plos.org/phishing.

If we can help with anything else, please email us at customercare@plos.org.

Thank you for submitting your work to PLOS One and supporting open access.

Kind regards,

PLOS ONE Editorial Office Staff

on behalf of

Dr. Xiaoen Wei

Academic Editor

PLOS One
